# HIF Regulates Multiple Translated Endogenous Retroviruses: Implications for Cancer Immunotherapy

**DOI:** 10.1016/j.cell.2025.01.046

**Published:** 2025-02-28

**Authors:** Qinqin Jiang, David A. Braun, Karl R. Clauser, Vijyendra Ramesh, Nitin H. Shirole, Joseph E. Duke-Cohan, Nancy Nabilsi, Nicholas J. Karmer, Cleo Forman, Isabelle E. Lippincott, Susan Klaeger, Kshiti M. Phulphagar, Vipheaviny Chea, Nawoo Kim, Allison P. Vanasse, Eddy Saad, Teagan Parsons, Melissa Carr-Reynolds, Isabel Carulli, Katarina Pinjusic, Yijia Jiang, Rong Li, Sudeepa Syamala, Suzanna Rachimi, Eva K. Verzani, Jonathan D. Stevens, William J. Lane, Sabrina Y. Camp, Kevin Meli, Melissa B. Pappalardi, Zachary T. Herbert, Xintao Qiu, Paloma Cejas, Henry W. Long, Sachet A. Shukla, Eliezer M. Van Allen, Toni K. Choueiri, L. Stirling Churchman, Jennifer G. Abelin, Cagan Gurer, Gavin MacBeath, Richard W. Childs, Steven A. Carr, Derin B. Keskin, Catherine J. Wu, William G. Kaelin

**Affiliations:** 1 Department of Medical Oncology, Dana-Farber Cancer Institute, Harvard Medical School, Boston, MA 02215, USA; 2 Lank Center for Genitourinary Oncology, Dana-Farber Cancer Institute, Harvard Medical School, Boston, MA 02215, USA; 3 Yale Center of Cellular and Molecular Oncology, Yale School of Medicine, New Haven, CT 06511, USA; 4 Broad Institute of MIT and Harvard, Cambridge, MA 02142, USA; 5 Translational Immunogenomics Laboratory, Dana-Farber Cancer Institute, Boston, MA 02215, USA; 6 TScan Therapeutics, Waltham, MA 02451, USA; 7 Department of Genetics, Blavatnik Institute, Harvard Medical School, Boston, MA 02215, USA; 8 Center for Functional Cancer Epigenetics, Dana-Farber Cancer Institute, Boston, MA 02215, USA; 9 Department of Pathology, Brigham and Women’s Hospital, Boston, MA 02215, USA; 10 Cancer Epigenetics Research Unit, Oncology, GSK, Collegeville, PA 19426, USA; 11 Molecular Biology Core Facilities, Dana-Farber Cancer Institute, Boston, MA 02215, USA; 12 Department of Hematopoietic Biology and Malignancy, The University of Texas MD Anderson Cancer Center, Houston, TX 77030, USA; 13 Department of Medicine, Brigham and Women’s Hospital, Boston, MA 02215, USA; 14 Laboratory of Transplantation Immunotherapy, Cellular and Molecular Therapeutics Branch, National Heart Lung and Blood Institute, National Institutes of Health, Bethesda, MD 20892, USA; 15 Department of Computer Science, Metropolitan College, Boston University, Boston, MA 02215, USA; 16 Section for Bioinformatics, Department of Health Technology, Technical University of Denmark, Lyngby 2800, Denmark; 17 Howard Hughes Medical Institute, Chevy Chase, MD 20815, USA; 18 These authors contributed equally; 19 Lead contact

**Keywords:** HIF, ccRCC, ERV, neoantigen, cancer vaccine, immunotherapy Introduction

## Abstract

Clear cell renal cell carcinoma (ccRCC), despite having a low mutational burden, is considered immunogenic because it occasionally undergoes spontaneous regressions and often responds to immunotherapies. The signature lesion in ccRCC is inactivation of the *VHL* tumor suppressor gene and consequent upregulation of the HIF transcription factor. An earlier case report described a ccRCC patient who was cured by an allogeneic stem cell transplant and later found to have donor-derived T cells that recognized a ccRCC-specific peptide encoded by a HIF-responsive endogenous retrovirus (ERVE-4). We report that ERVE-4 is one of many ERVs that are induced by HIF, translated into HLA-bound peptides in ccRCCs, and capable of generating antigen-specific T cell responses. Moreover, ERV expression can be induced in non-ccRCC tumors with clinical-grade HIF stabilizers. These findings have implications for leveraging ERVs for cancer immunotherapy.

## In Brief

An upregulation of the HIF2 transcription factor, which is a hallmark of the most common form of human kidney cancer, drives the expression of multiple relatively intact endogenous retroviruses, including some that are presented as HLA-bound peptides that can be recognized by autologous T cells after immune checkpoint inhibitors or allogeneic T cells after stem cell transplantation

## Graphical Abstract

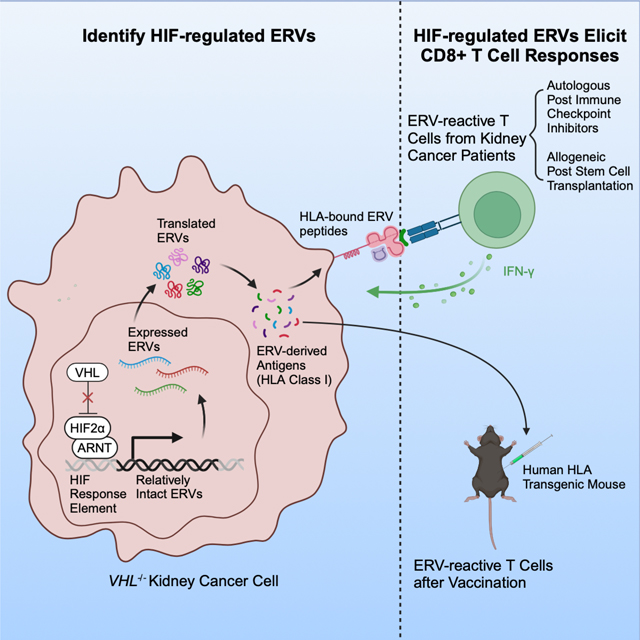

## Introduction

Immune-checkpoint inhibitors (ICIs) that block CTLA4, PD1, or PD-L1 are highly efficacious and potentially curative treatments for multiple cancer types^1–3^. Nonetheless, some tumor types are characteristically unresponsive to ICIs, and their effectiveness is highly variable, even for those tumor types where they are approved.

The response of melanomas, mismatch repair-defective colon cancers, and non-small lung cancers to ICIs is believed to be driven by their high mutational burdens, which is a potentially rich source of neoantigens^3–5^. In contrast, clear cell renal cell carcinomas (ccRCCs), the most common form of kidney cancer, also frequently respond to ICIs despite having relatively few non-synonymous mutations relative to other immunogenic tumor types^6,7^. ccRCCs do have many insertion and deletion (INDEL) mutations^8^, but INDEL burden does not predict ICI responsiveness in this setting^9–11^.

However, the response of ccRCC to ICIs was unsurprising because ccRCC has historically been considered an “immunogenic” tumor. Although rare, ccRCCs sometimes undergo spontaneous remissions^12^. High dose interleukin-2 is active against ccRCC and occasionally produced durable remissions, but has been largely abandoned due to its toxicity and the availability of alternative therapies^13–15^. Among solid tumors, ccRCCs have particularly high levels of infiltrating effector T cells^16^. In contrast to other tumor types, however, increased T cell infiltration is an adverse prognostic biomarker in ccRCC^17^ and an inconsistent predictor of ICI response^10^.

Inactivation of the pVHL tumor suppressor protein is the initiating event in most ccRCCs^18^. pVHL forms a ubiquitin ligase that targets the alpha subunits of the heterodimeric HIF transcription factor for degradation^18^. pVHL only recognizes HIFα after HIFα has been prolyl hydroxylated by oxygen-sensitive EglN dioxygenases^19^. Failure to degrade HIF2α, drives the growth of ccRCC^20^. An allosteric HIF2α inhibitor, belzutifan, was recently approved for treating ccRCC^21,22^. Conversely, EglN inhibitors that stabilize HIF2α are used to treat anemia^18^.

Endogenous retroviruses (ERVs) and remnants thereof [including solo long terminal repeats (LTRs)] occupy up to 8 percent of the human genome^23,24^, but are often viewed as vestigial due to rearrangements and other mutations. It is clear, however, that some ERVs can be transcribed and translated^16,25–27^. ERV expression has been documented in many cancers, possibly reflecting, at least in part, their characteristic global DNA hypomethylation because DNA methylation usually represses ERV transcription^25,28–32^. Several studies showed that ccRCCs express high levels of ERVs relative to other solid tumors and that ERV expression correlates with their likelihood of responding to ICIs^16,33–36^.

Over two decades ago, Richard Childs and coworkers at the NIH treated 74 advanced ccRCC patients with allogeneic stem cell transplants (allo-SCTs) as potential sources of immunoreactive T cells^37,38^. Almost half the patients had measurable responses, including 11.7% who had complete responses (CRs)^38^. In one complete responder, they identified circulating donor-derived T cells that reacted with an HLA-A*11-bound peptide (ATFLGSLTWK) derived from hERV-E (now called ERVE-4) on the patient’s tumor cells and that specifically killed those tumor cells *ex vivo* and a subset of HLA-A*11+ ccRCC lines^38^. ERVE-4 expression was detectable in ccRCCs, but not in normal tissues or other cancers^38,39^. They subsequently showed that ERVE-4 was regulated by HIF2, potentially explaining its upregulation in ccRCC^39,40^. We asked if ERVE-4 is unique in this regard, or a prototype of other potentially immunogenic HIF2responsive ERVs in ccRCC.

## Results

### Regulation of ERV3.2 and ERV4700 by pVHL and HIF2

Vargiu and coworkers annotated 3173 ERVs in the human genome that are relatively intact, and hence potentially transcribed and translated, including ERVE-4, ERV3.2, and ERV4700 (ERVE-4 and ERV3.2 are ERV2256 and ERV2637, respectively, using the Vargiu nomenclature)^41^. Increased expression of these ERVs correlates with the likelihood of ccRCCs responding to ICIs in some studies, and peptides derived from ERVE-4 and ERV4700 can elicit T cell responses^33–36,38^.

**Figure 1.**
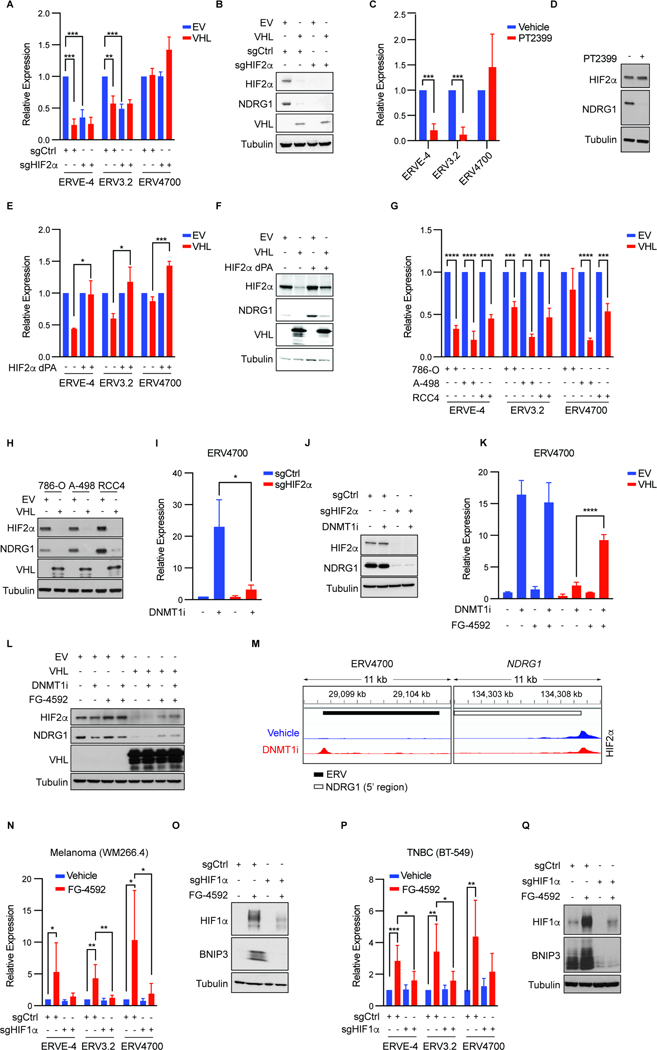
Regulation of ERV3.2 and ERV4700 by pVHL and HIF2 (A, C, E, G) ERV RNA levels in 786-O cells (A, C, E) or the indicated ccRCC cell lines (G) that were stably infected to express pVHL (A, E, G) or treated for 72 hours with 2 μM PT2399 (PT) (C) where indicated in red. The cells first underwent CRISPR-based gene editing with a HIF2α sgRNA (sgHIF2α) or a control sgRNA (sgCtrl) (A) or were stably infected to express the HIF2α dPA (P405A, P531A) variant (E) where indicated. (N=3) (B, D, F, H) Immunoblots of cells as in (A, C, E, G). (I and M) ERV4700 RNA levels (I) (N=3) and anti-HIF2α ChIP-seq of the ERV4700 locus (M) in 786-O cells treated with 0.5 μM of the GSK3685032 (DNMT1i) or vehicle (Veh) for 9 days. In (I) the cells were first edited with a sgHIF2α or a sgCtrl guide. In (M) *NDRG1* locus was included as a control. See also Figure S2G. (J) Immunoblots of cells as in (I). (K and L) ERV4700 RNA levels (K) (N=3) and immunoblot analysis (L) of 786-O(VHL) or 786-O(EV) cells treated with 0.5 μM GSK3685032 (DNMT1i), 100 μM FG-4592,or both for 9 days. (N, P) ERV RNA levels of WM266–4 melanoma cells (N) and BT-549 TNBC cells (P) treated with 50 μM FG-4592 or Veh for 9 days. The cells were first edited with a HIF1α sgRNA or a sgCtr guide. (N=6) For all RT-qPCR panels, ERV RNA levels were normalized to *ACTB* RNA levels and then to the first condition listed. (O and Q) Immunoblots of cells of (N and P) on day 3.

To ask if ERV3.2 and ERV4700 are, like ERVE-4, regulated by HIF2, we performed quantitative Real Time-PCR (RT-qPCR) assays on 786-O *VHL*^*−/−*^ ccRCC cells. Due to the sequence similarities of many ERVs, we carefully designed RT-qPCR primers that would measure specific ERVs. ERV3.2 RNA levels, like ERVE-4 levels, were suppressed in 786-O cells by lentiviral reintroduction of pVHL, CRISPR/Cas9-mediated elimination of HIF2α, or treatment with the allosteric HIF2α inhibitor PT2399^42−44^ (Figures 1A–1D). The HIF2-responsive mRNA product NDRG1 served as a control. Moreover, suppression of ERV3.2 and ERVE-4 RNA levels by pVHL was reversed by lentiviral expression of a constitutively stable version of HIF2α (HIF2α dPA)^45,46^ (Figures 1E and 1F). Therefore ERV3.2, like ERVE-4, is HIF2-responsive.

Since ERV4700 RNA levels were not repressed by either pVHL or PT2399 in 786-O cells (Figures 1A and 1C), we interrogated additional *VHL*^*−/−*^ ccRCC cell lines. Notably, ERV4700 RNA levels were suppressed after reintroduction of pVHL into A-498 cells and RCC4 cells, suggesting that epigenetic differences between the three cells lines affect the regulation of ERV4700 by HIF2 (Figures 1G and 1H).

### Pharmacological Induction of HIF-responsive ERVs

Consistent with this hypothesis, short-term treatment of 786-O cells with the DNA methyltransferase 1 (DNMT1) inhibitor GSK3685032^47,48^ dramatically induced ERV4700 RNA levels (Figures 1I and 1J). This induction was largely ablated by CRISPR/Cas9-mediated elimination of HIF2α and by reintroduction of pVHL (Figures 1I–1L). The effect of pVHL reintroduction, however, was largely mitigated by the EglN inhibitor FG-4592^18^, which prevents the recognition of HIF1α and HIF2α by pVHL, thereby increasing HIF stability and activity (Figures 1K and 1L). 786-O cells express exclusively HIF2α^49^. The induction of ERV4700 by GSK3685032 coincided with the emergence of a neighboring HIF2α-binding peak in chromatin immunoprecipitation sequencing (ChIP-seq) experiments (Figure 1M). Therefore, HIF2 regulates, directly or indirectly, ERV3.2 and ERV4700, although the latter is impacted by DNA methylation.

This knowledge predicted that these ERVs could be pharmacologically induced in non-kidney, *VHL*^*+/+*^, cancers using FG-4592. Indeed, FG-4592 increased ERVE-4, ERV3.2, and ERV4700 expression in BT-549 breast cancer cells, WM266–4 melanoma cells, and MGG152 glioblastoma cells (Figures 1N–1Q and S1A). The HIF1-responsive *BNIP3* mRNA was used as a control because these three cell lines, in contrast ccRCC lines, primarily express HIF1α instead of HIF2α (Figure S1B). ERV induction by FG-4592 in WM266–4 and BT-549 cells was ablated after HIF1α inactivation using CRISPR/Cas9 (Figures 1N–1Q). We failed to inactivate HIF1α in MGG152 cells.

SETDB1 histone H3K9 methyltransferase represses some ERVs^50,51^. CRISPR-mediated elimination of SETDB1 further increased ERVE-4 and ERV4700 levels, but not ERV3.2 levels, in 786-O cells (Figures S1C and S1D). In contrast, eliminating SETDB1 did not induce these three ERVs in WM266–4 cells irrespective of FG-4592 (Figures S1E and S1F). This suggests that repression of ERVs by SETDB1 is both ERV- and cell context-dependent.

### Human HIF2-responsive ERVs in RCC cell lines

**Figure 2.**
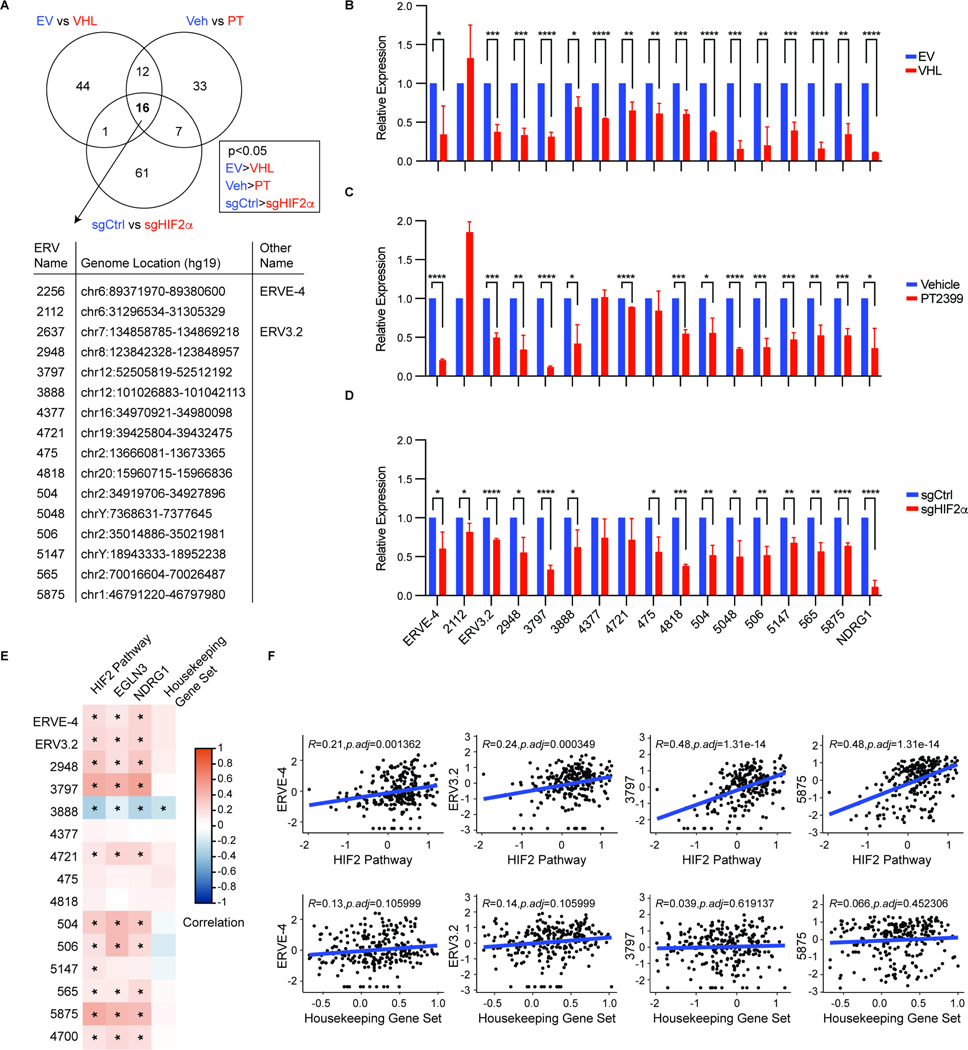
Identification of HIF2-responsive ERVs by RNA-seq (A-D) Venn Diagram (A) and RT-qPCR validation (B-D) of HIF2-responsive ERVs in 786-O(VHL) or 786-O(EV) cells, treated with 2 μM PT or Veh for 72 hours, or edited with a sgHIF2α or a sgCtrl guide. *NDRG1* mRNA levels served as controls. ERV RNA levels were normalized to *ACTB* RNA levels and then to the EV, Veh or sgCtrl cells. (E) Correlation matrix (Pearson’s R) between the expression of 15 ERVs and the expression of a validated HIF2 signature^43^, the HIF2-responsive genes *EGLN3* and *NDRG1*, and a 42-gene housekeeping gene signature^92^, based on RNA-seq data of 250 advanced ccRCCs from the CheckMate 025 trial^10^. (F) Examples of four ERVs from (E). Shown are Pearson’s correlations between ERV expression levels and expression of a HIF2 pathway signature (upper) and a housekeeping gene set signature (lower).

To look for human HIF2-responsive ERVs more systematically, we generated RNA sequencing (RNA-seq) data from 786-O cells that were either 1) infected with a lentivirus expressing *VHL* [hereafter 786-O(VHL) cells] or the empty vector [hereafter 786-O(EV) cells], 2) subjected to CRISPR/Cas9-based editing with a HIF2α sgRNA or control sgRNA or 3) treated with PT2399 or vehicle, thus enabling 3 pairwise comparisons of 786-O cells in which HIF2α activity was low (shown in blue) or high (shown in red) (Figure 2A). We then examined the RNA abundance of the 3173 relatively intact ERVs annotated by Vargiu and coworkers^41^. Sixteen ERVs, including ERVE-4 and ERV3.2, scored as HIF2α-dependent in all three comparisons, while an additional 20 scored in two of three comparisons (Figure 2A and Table S1). All ERVs that scored in all three comparisons retested positively in at least one, and usually all three, of the pairwise comparisons in RT-qPCR assays (Figures 2B–2D). In addition, 16/20 of the ERVs that scored in 2 of 3 comparisons validated in at least one of three comparisons by RT-qPCR (Figures S1G–S1I).

Next we queried RNA-seq data from 250 ccRCC patients treated on the nivolumab vs everolimus CheckMate 025 trial^10^ in order to validate the association of HIF activity and the expression of HIF-regulated ERVs in a large cohort of ccRCC patients. Expression data were available for 14 of the 16 ERVs identified in the 786-O cells, in addition to ERV4700. The expression of 11 of the 15 ERVs detected, including ERV4700, correlated with a previously validated HIF2 signature^43^ and, in 10/11 cases, with individual HIF2 target genes such as *EglN3* and *NDRG1* (Figures 2E, 2F and Table S1). Therefore many of the HIF2-responsive ERVs we detected in 786-O cells are likely to be activated by HIF2α in ccRCC cells *in vivo* (see also below).

RNA-seq experiments analogous to those performed with the 786-O cells revealed 9 ERVs in A-498 *VHL*^−/−^ ccRCC cells that were HIF2-responsive in all three isogenic comparisons, including ERV4700 (Figures S1J–S1L and Table S1). Six of the 9 ERVs were also identified in the 786-O cells, including ERVE-4 and ERV3.2 (Figure S1M). Many of these ERVs were responsive to PT2399 in other *VHL*^*−/−*^ ccRCC lines, with greater responsiveness in the HIF2-dependent lines OS-RC-2 and THUR4TKB compared to the HIF2-independent lines 769-P and Caki-2^52^ (Figures S1N–S1Q).

### Human ERVs that are Directly Regulated by HIF2 in RCC cell lines

**Figure 3.**
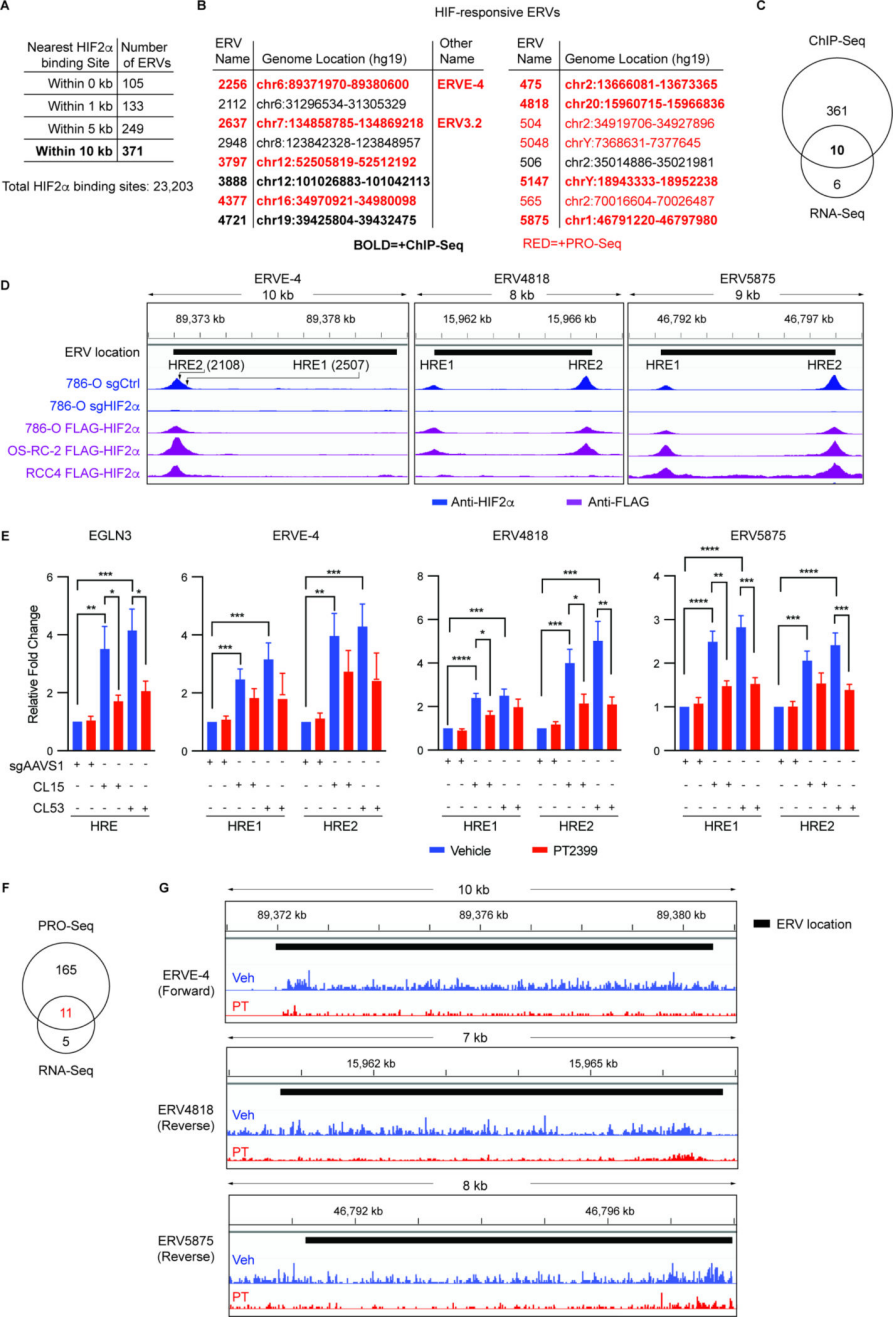
Identification of ERVs that are Directly Regulated by HIF2 (A) Number of ERVs with neighboring HIF2α binding sites. (B) HIF2-responsive ERVs identified by RNA-seq from Figure 2A. ERVs in bold are located within 10 kb of a HIF2α binding site based on anti-HIF2α ChIP-seq. ERVs in red are direct HIF2α targets based on PRO-seq. (C) Venn Diagram showing overlap of 16 HIF2-responsive ERVs (B) and ERVs located within 10 kb of a HIF2α binding site. (D) Representative Anti-HIF2α ChIP-seq tracks (blue) in 786-O cells that underwent CRISPR-based gene editing with a HIF2α sgRNA or a control sgRNA (sgCtrl) and representative Anti-FLAG ChIP-seq tracks (purple) in 786-O, OS-RC-2 and RCC4 cells in which a FLAG-HA epitope tag coding sequence was inserted at the 5’ end of the endogenous *HIF2*α open reading frame by CRISPR-HDR (FLAG-HIF2α). See also Figure S2A. (E) Anti-FLAG ChIP-qPCR assays using two different clones (CL15 and CL53) of 786-O FLAG-HIF2α cells compared to parental cells that were infected with a sgAAVS1 control guide. Cells were treated with 2 μM PT or Veh for 72 hours prior to ChIP-qPCR with primers specific for the putative HREs for the indicated ERVs. *EglN3* served as a control. Data were normalized to the untreated sgAAVS1 cells. (F) Venn Diagram showing overlap of 16 HIF2-responsive ERVs (B) and ERVs scored as HIF2α direct targets by PRO-seq. (G) Representative nascent RNA tracks in OS-RC-2 cells that were treated with 2 μM PT2399 or vehicle for 2 hours.

Genome-wide ChIP-seq experiments using an anti-HIF2α antibody and isogenic 786-O cells in which HIF2α was or was not eliminated using CRISPR/Cas9 identified over 20,000 high confidence HIF2α binding sites (Table S2). One hundred and five ERVs contained a superimposed HIF2α-binding site, while 371 ERVs had a HIF2α-binding site within 10 kb (Figure 3A). Ten of the 16 HIF2-responsive ERVs identified by RNA-seq had at least one HIF2α binding site within 10 kb, including ERVE-4 and ERV3.2 (Figures 3B and 3C).

To further enhance detection of HIF2α-binding sites, we also did ChIP-seq using an anti-FLAG antibody and ccRCC cells (786-O, OS-RC-2, and RCC4) in which CRISPR-based homologous recombination was used to append a FLAG-HA epitope tag to the N-terminus of HIF2α expressed from the endogenous *HIF2*α locus. For ERVE-4, a putative HIF2α-binding site reported by Childs and coworkers was on the “shoulder” of the ChIP-seq signal, suggesting the presence of 2 closely spaced HIF2α-binding sites^40^ (Figures 3D and S2A). Both binding sites appeared to be functional based on ChIP-qPCR assays using two 786-O FLAG-HA-HIF2α subclones (CL15 and CL53) that were treated or not with PT2399 (Figures 3E and S2B). 786-O cells edited with a negative control sgRNA (sgAAVS1)^53^ served as controls.

Among the 10 HIF2-responsive ERVs with neighboring HIF2α-binding sites were ERV4818 and ERV5875, both of which were bracketed by HIF2α-binding sites that validated in ChIP-qPCR assays (Figures 3D, 3E and S2A). In total, we authenticated at least one neighboring HIF2α-binding site for 7 of the 10 HIF2α-responsive ERVs that scored by ChIP-seq, including ERV3.2, by ChIP-qPCR (Figures 3E and S2C). Moreover, genome-wide ChIP-seq revealed that ERV4700 was one of the 28 ERVs that were induced by the DNMT1 inhibitor GSK3685032 in a HIF2-dependent manner in 786-O cells, including additional ERVs with HIF2 binding sites within 10 kb (Figures S2D–S2G and Table S1).

HIF2 can, like many enhancer-binding transcription factors, act at considerable distances when distance is measured in nucleotides. Therefore, the arbitrary 10 kb cutoff we used likely underestimates the number of ERVs that are directly regulated by HIF2α. Conversely, a HIF2α-binding site very close to a given gene need not be transcriptionally relevant. We therefore also performed precision nuclear run-on sequencing (PRO-seq) to identify areas of active transcription, as determined by recruitment of active RNA polymerases, following perturbation of HIF2 signaling^54–56^. In preliminary experiments we obtained higher quality PRO-seq reads in OS-RC-2 cells, which are highly HIF2-dependent, compared to 786-O cells, which are moderately HIF2-dependent. We therefore used the OS-RC-2 PRO-seq data, which indicated that 11 of 16 ERVs induced by HIF2α at the RNA level in 786-O cells were directly regulated by HIF2α based on elimination of their nascent transcripts within 2 hours of treatment with PT2399, including 8 ERVs within 10 kb of a HIF2α-binding site (Figures 3F, 3G, S1N, S3 and Table S2).

### Human HIF2-responsive ERVs are translated in RCC cell lines

**Figure 4.**
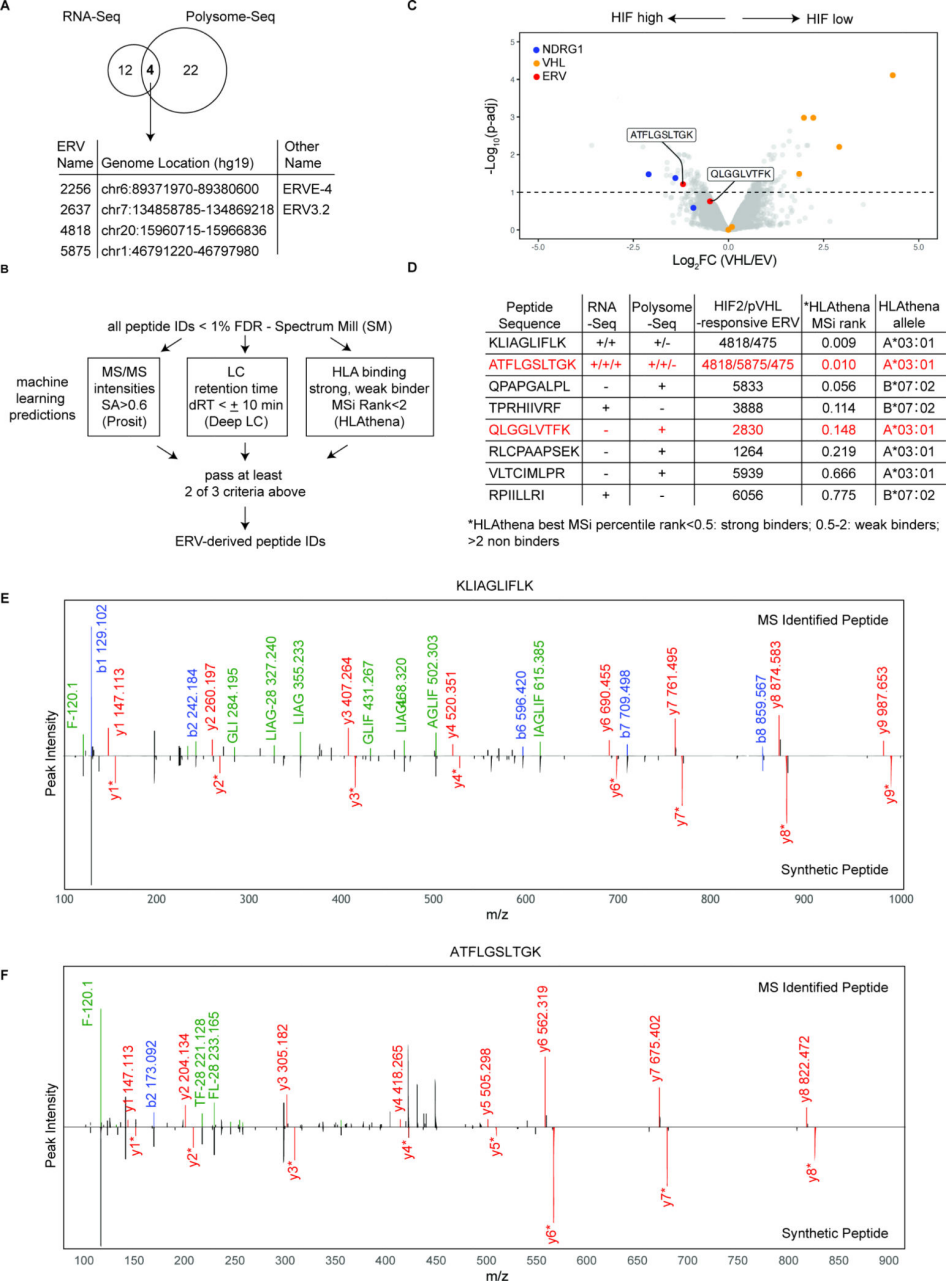
Identification of HLA-bound ERV-derived Peptides (A) Venn Diagram showing overlap of 16 HIF2-responsive ERVs with 26 pVHL-responsive translated ERVs based on Polysome-seq of 786-O(VHL) or 786-O(EV) cells. (B) Filtering approach applied to LC-MS/MS peptide identification for ERV-derived peptides. SA(spectral angle); LC(liquid chromatography); dRT(delta retention time). (C) HLA-bound peptides derived from the human proteome or from the 57 ERVs that scored in two of the three pairwise 786-O cell comparisons (high HIF2 versus low HIF2) and/or scored as pVHL-responsive by Polysome-seq. The volcano plot depicts the relative amount of each peptide as determined by anti-HLA immunoprecipitation of 786-O(VHL) or 786-O(EV) cells treated with 100 ng/ml IFNγ for 72 h, followed by TMT labeling and MS. Note the enrichment of pVHL-derived peptides in the 786-O(VHL) cells relative to the 786-O(EV) cells (right side of volcano plot) and enrichment of peptides from the HIF-responsive gene product NDRG1 in the 786-O(EV) cells (left side of the volcano plot). (D) ERV-derived peptides identified in (C). Note that some peptides could be encoded by more than one homologous ERV from among the 57 ERVs interrogated in (C). The ERVs scored as HIF2-responsive by RNA-seq and/or pVHL-responsive by Polysome-seq as indicated by the “+”. (C and D) Shown in red are two potentially pVHL-responsive ERV-derived peptides. (E and F) MS/MS spectra comparing HLA-bound peptides determined to be KLIAGLIFLK (E) and ATFLGSLTGK (F) compared to the corresponding synthetic peptides bearing a heavy lysine (Lys8).

We next performed Polysome-seq^57,58^ to identify additional HIF2-responsive ERVs that might, like ERVE-4 and ERV4700, be translated (Table S3)^33,38,39^. Four of the 16 ERVs (ERVE-4, ERV3.2, ERV4818 and ERV5875) that scored as HIF2-responsive in 786-O cells in all three isogenic comparisons associated with polysomes (indicative of active translation) in 786-O cells in a pVHL-suppressible manner (Figures 4A, S4A and S4B). An additional polysome-associated and pVHL-suppressible ERV (ERV3895) had scored and validated as HIF2-responsive in two of the three RNA-seq isogenic comparisons (Figures S1G–S1I) and we confirmed its association with polysomes was pVHL-dependent by RT-qPCR (Figures S4A and S4B). Similar studies suggested that 3 of the 9 HIF2-responsive ERVs in A-498 cells were translated: ERVE-4, ERV2651, and, as expected, ERV4700 (Figures S4C, S4D and Table S3).

### ERV-derived HLA-bound peptides in human RCC cell lines

To determine if any of the HIF2-responsive ERVs we identified were processed and presented by HLA class I molecules on cancer cells, we analyzed immunoprecipitated HLA complexes from 786-O(VHL) and 786-O(EV) cells by liquid chromatography-tandem mass spectrometry (LC-MS/MS) and searched the data for ERV-derived peptides (STAR Methods). The cells were treated with interferon gamma (IFNγ) to increase HLA abundance and potentially the sensitivity of our assays (Figures S4E–S4G). We liberalized the ERV search space to include all the ERVs that scored in at least two of the three pairwise 786-O cell comparisons (high HIF2 versus low HIF2) or scored in the Polysome-seq data as pVHL-responsive, or both, for a total of 57 ERVs (Table S4). We eliminated peptides that mapped to the canonical reference proteome (in addition to one or more ERVs) and retained, but annotated, peptides that mapped to both non-coding sources and ERVs. We also listed all possible ERVs for peptides that could be produced by multiple ERVs (Table S4). For additional rigor we applied scoring filters based on scores from machine learning tools for prediction of MS/MS ion intensity, LC retention time and HLA binding (Figure 4B).

In total, we detected 8 HLA-bound, ERV-derived, peptides using isobaric mass tag labeling with TMT and label free MS, including 3 peptides that were recovered using both methods (Figures 4C, 4D and Table S4). Among the peptides predicted to be strong binders to 786-O HLAs was the peptide KLIAGLIFLK, which could be derived from HIF2-responsive ERV4818 or ERV475 because of their high degree of sequence homology; and the peptide ATFLGSLTGK, which could be derived from ERV5875, ERV4818 or ERV475. We confirmed the sequence identities of these two peptides using synthetic peptide standards (Figures 4E and 4F). Notably, the ATFLGSLTGK peptide differed by only at a single amino acid compared to the peptide previously identified by Childs and coworkers (ATFLGSLTWK)^38^, which we did not detect. Although it was discovered in the context of HLA-A*11-expressing cells (HLAthena MSi rank:0.035), the ATFLGSLTWK peptide is also predicted to bind to HLA-A*03:01 (HLAthena MSi rank:0.030), which is present on 786-O cells. Our failure to detect this peptide could therefore reflect the limitations of the current algorithms for predicting HLA Class I binding or an IP-MS false-negative.

Analysis of the 12,763 HLA-bound peptides observed in the TMT LC-MS/MS of 786-O cells, including both mRNA- and ERV-derived peptides, identified upregulated pVHL-derived peptides in the 786-O(VHL) cells and upregulated peptides from the HIF2-responsive gene product NDRG1 in the 786-O(EV) cells (Figure 4C). The ERV-derived peptide ATFLGSLTGK was, similar to the NDRG1-derived peptides, among the most highly upregulated HLA-bound peptides in the 786-O(EV) cells (Figure 4C). Surprisingly, many of the other ERV-derived peptides, including the KLIAGLIFLK peptide, were not pVHL-responsive based on our MS studies. For KLIAGLIFLK, this likely reflects the fact that it can be encoded by many other ERVs, including the highly expressed, but pVHL-unresponsive, ERV6169, due to sequence homology (Figures S4H–S4K).

A-498 cells express HLA-A*02:01, HLA-B*08:01 and HLA-C*07:01. We identified 4 HLA-bound, ERV-derived peptides using label-free LC-MS/MS of anti-HLA immunoprecipitates from isogenic A-498(VHL) and A-498(EV) cells (Figures S4E, S4L, S4M and Table S4). Our search focused on ERVs that scored in at least two of the three pairwise A-498 cell comparisons (high HIF2 versus low HIF2) or scored in our A-498 Polysome-seq as being pVHL-responsive, or both, for a total of 31 possible ERVs (Table S4).

### HIF2-responsive ERVs are largely derived from cancer cells

**Figure 5.**
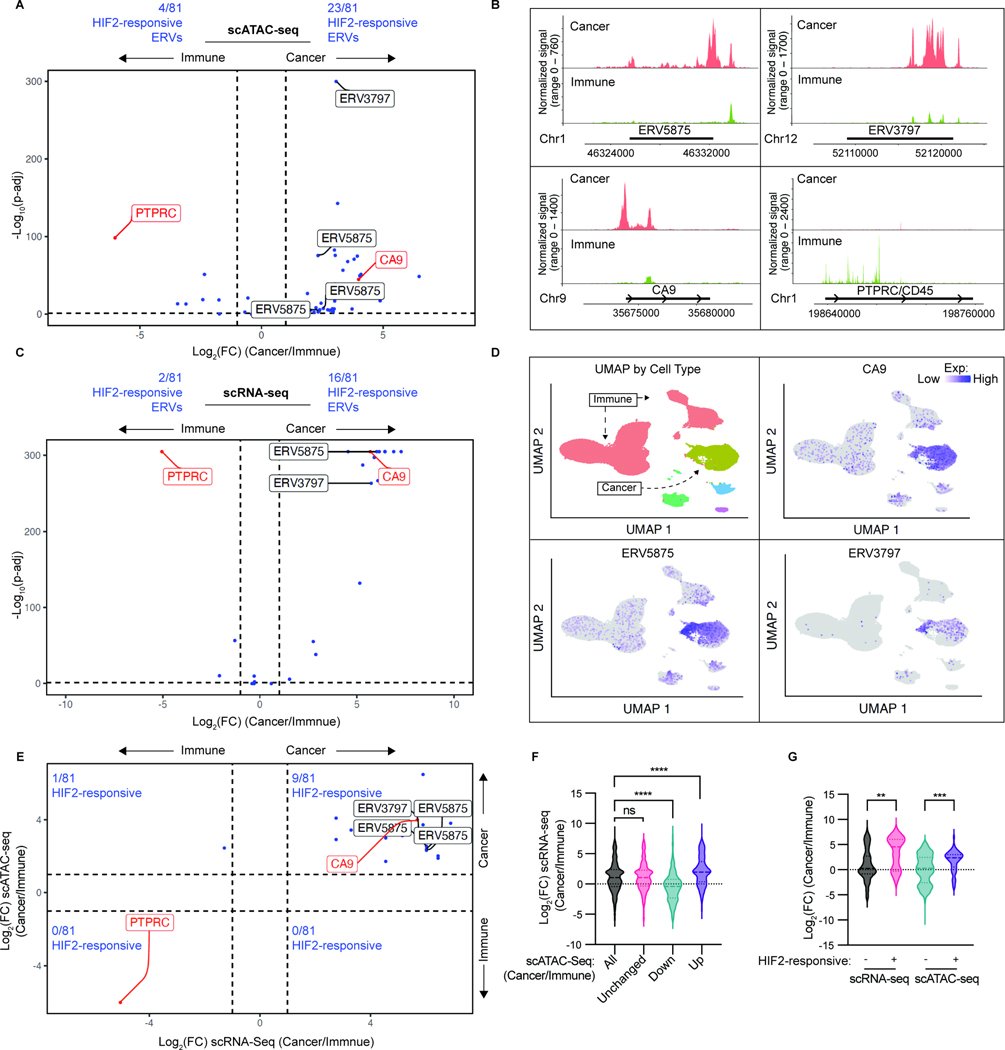
Human HIF2-responsive ERVs are Largely Derived from Cancer Cells (A and C) Volcano plots describing differential chromatin accessibility (A) and differential expression (C) derived from scATAC-seq data and scRNA-seq data from 16 ccRCCs^60^ for the 81 HIF2-responsive ERVs nominated by our cell line analyses. The cancer cell-specific *CA9* and immune cell-specific *PTPRC* genes (highlighted in red), served as controls. The vertical dashed lines indicate |Log(2)FC| of 1 and p-adj of 0.05. (B) Coverage plot showing single-cell ATAC-seq tracks for the indicated genes (± 3 kb). (D) UMAP of scRNA-seq data by cell type, followed by feature plots for ERV5875 and ERV3797. (E) Correlation of ERV expression with ERV chromatin accessibility based on the data in (A) and (C). The vertical and horizontal dashed lines indicate |Log(2)FC| of 1 and p-adj of 0.05. (F-G) Violin plots of all ERV expression by scATAC-seq peaks defined as ‘All’, ‘Unchanged’, ‘Down’ or ‘Up’ (cancer vs immune) (F) or all ERV expression and chromatin accessibility by HIF2-responsiveness (G).

ERVs can also be expressed in immune cells and a recent study suggested that this explained the correlation between ERV expression in ccRCCs and their responsiveness to ICIs^59^. Our cell line data strongly suggests that ccRCC cells themselves can express certain ERVs. To ask whether the HIF2-responsive ERVs we identified were expressed by cancer cells in human ccRCCs, we analyzed publicly available single-cell ATAC-seq (scATAC-seq) and single-cell RNA-seq (scRNA-seq) data from 16 ccRCCs^60^. We focused on 81 non-redundant ERVs that scored in two of the three pairwise cell comparisons (high HIF2 versus low HIF2) and/or scored as translated and pVHL-responsive by Polysome-seq in either 786-O cells or A-498 cells. Twenty-three of the 81 HIF-responsive ERVs displayed greater chromatin accessibility in the cancer cells relative to the infiltrating immune cells, compared to 4/81 that showed the opposite pattern (Figures 5A, 5B, S5A, S5B and Table S5). One ERV (ERV6084) was excluded as there were two adjacent peaks showing opposite patterns. Four of the 81 showed peaks with comparable accessibility across the two cell compartments while the remaining 49 did not have detectable scATAC-seq peaks. Promoter associated peaks at the ccRCC marker *CA9* and immune cell marker *PTPRC* served as controls.

Similarly, 16 of the 81 HIF-responsive ERVs were more highly expressed in cancer cells relative to the infiltrating immune cells, compared to 2/81 that showed the opposite pattern (Figures 5C, 5D and Table S5). Five of the 81 showed comparable expression in the two cell compartments while the remaining 58 were undetectable. As expected, increased chromatin accessibility of the ERVs was generally associated with increased ERV expression (Figure 5E). The expression of individual ERVs was somewhat heterogeneous between tumors, presumably due to intertumoral genetic and epigenetic differences (Figure S5C). For comparison, we also investigated all 3173 ERVs in the Vargiu database^41^. ERV chromatin accessibility generally correlated positively with ERV expression, as expected, in this broader dataset (Figure 5F). Notably, the 81 evaluable HIF-responsive ERVs were significantly more accessible and more highly expressed in cancer cells relative to immune cells, while no such differential was seen for the remaining evaluable ERVs (Figure 5G). Finally, we examined the expression of the 81 ERVs using RNA-seq performed on CA9-purified cancer cells derived from 8 ccRCCs, including one primary and metastasis pair from the same patient. The expression of individual ERVs, such as ERV4700, varied between tumors. Nonetheless, all 81 ERVs were detected in at least one of the CA9-enriched tumor samples (Figure S5D). We conclude that many of the HIF2-responsive ERVs we identified are more highly expressed in cancer cells than by infiltrating immune cells.

### ERV-derived HLA-bound peptides in human kidney tumors

**Figure 6.**
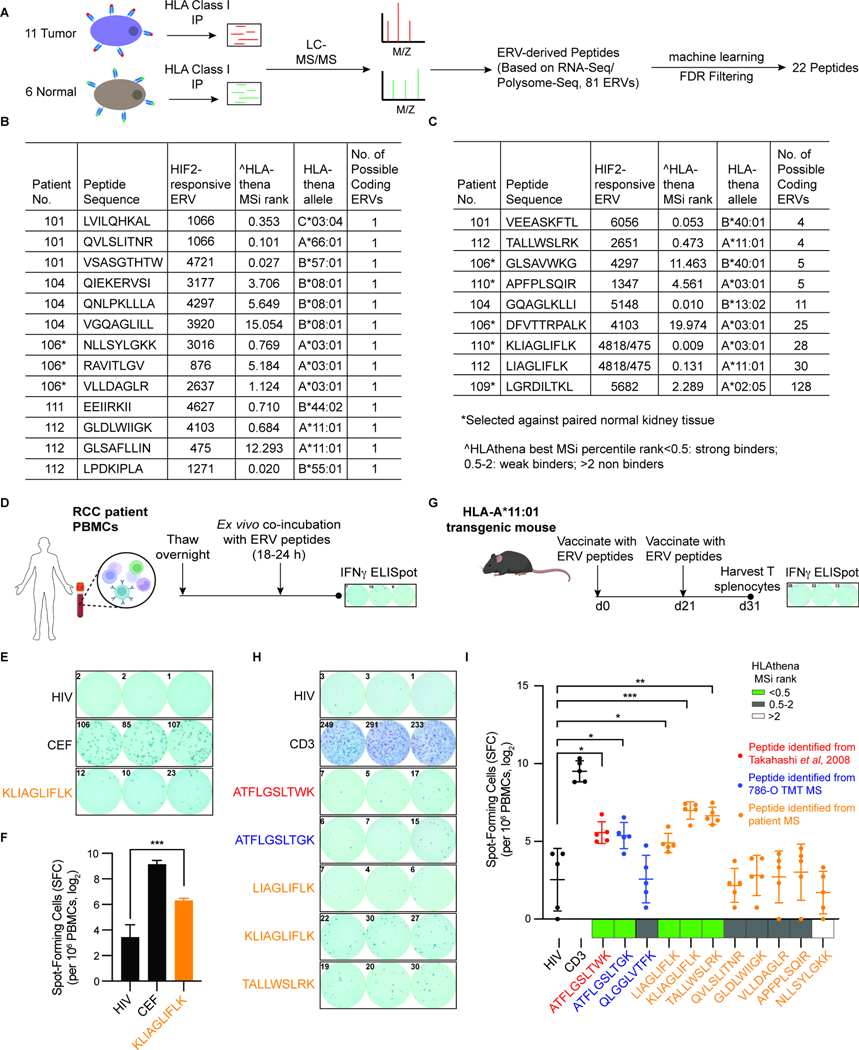
HLA-Bound and Immunogenic ERV-derived Peptides are Present in Human Kidney Cancer Samples (A) Workflow for identifying ERV-derived peptides in ccRCCs. (B and C) 22 ERV-derived peptides identified from 11 ccRCC patients. * indicates peptides that were tumor-specific based on analysis of available paired normal kidney tissue. “HIF2-responsive ERV” column indicates potential source ERV(s) for each peptide from the 81 ERVs identified in 786-O and A-498 cells. “No. of Possible Coding ERVs” column indicates number of potential source ERVs for the peptides. Peptides that uniquely mapped to one HIF2-responsive ERV are shown in (B). (D and G) Schema for quantifying IFNγ secreting T cells derived from PBMCs (D) and splenocytes (G) by ELISpot assay. (E and F) ELISpot images (E) and quantification (spot count) (F) of IFNγ secreting PBMCs derived from ccRCC patient 110 incubated with the indicated peptides. An HIV peptide and a pool of viral peptides (CMV, EBV and Influenza) (CEF peptides) served as negative and positive controls respectively. (N=3) (H and I) Five HLA-A*11:01 transgenic mice were immunized with a pool of 11 peptides as in (G). Ten days post second vaccination, splenocytes were harvested and incubated with individual peptides *ex vivo* and monitored by ELISpot assays. A HIV peptide and CD3 served as negative and positive controls respectively. Representative ELISpot images (H) and spot counts (I) are shown. (N=5)

To search for ERV-derived HLA-bound peptides in primary human ccRCCs, we performed anti-HLA LC-MS/MS analyses on tumor specimens obtained from 11 late stage (stage 3 or 4) ccRCC patients with various HLA allelotypes, including 6 with paired normal kidney tissue. From the ~4000 HLA-bound peptides identified per sample, we focused on peptides that could be encoded by the 81 non-redundant, potentially HIF2-responsive, ERVs described above (Figure 6A and Table S4). After applying subset-specific FDR filtering to the ERV-derived peptide spectrum matches so that distributions for each score metric improved to meet or exceed the aggregate distributions, we obtained an initial list of 60 ERV-derived peptides across all 11 patients (STAR Methods, Table S6). We excluded peptides that were also detected in any of the normal tissue samples, which left 37 tumor-specific peptides. We then eliminated one redundant peptide and two cysteine-containing peptides, the latter because of technical challenges related to validating such peptides in biological systems. Twelve peptides were eliminated because they failed at least two of the filters depicted in Figure 4B. In total, we identified 22 non-redundant ERV-derived peptides in the 11 tumors. ERV-derived peptides represented <0.1% of all peptides detected in the immunopeptidomes (Figures 6B, 6C and Table S6).

HLAthena predicted 13 of the 22 as HLA binders (i.e. HLAthena Msi rank < 2). For 8 of the 22, matched normal tissue was available, allowing us to conclude that they were tumor-specific. Thirteen of the 22 peptides could be unambiguously derived from a single source ERV, while the remaining 9 could be derived from 2–128 different ERVs because of the high degree of sequence homology between related ERVs (Figures 6B, 6C and Table S6).

Of the 22 ERV-derived peptides tested, only one (KLIAGLIFLK) stimulated peripheral blood T-cells from the corresponding patient(s) in IFNγ ELISpot assays (Figures 6D–6F). T cell reactivity was not detected for the sole patient (out of 11) for whom tumor-infiltrating lymphocytes (TILs) were available (4 peptides tested in total), possibly reflecting the hypofunctional state of terminally exhausted CD8+ T cells characteristic of late-stage RCC^61^.

To ask whether any of the 22 ERV-derived peptides were intrinsically immunogenic, we used them to vaccinate naïve immunocompetent mice that were engineered to express a human HLA-complex. Such humanized mice are available for 6 HLA allelotypes. Based on their predicted binding scores, 7 of the 22 peptides were predicted to bind (i.e. HLAthena Msi rank < 2) to HLA-A*11:01 (7 peptides total) compared to 0 or 1 peptide for the other available alleles. The two HIF2-responsive, ERV-derived peptides we identified in 786-O cells (ATFLGSLTGK and QLGGLVTFK) and the peptide identified by Childs and coworkers (ATFLGSLTWK) were also predicted to bind to HLA-A*11:01 (Table S6).

We thus immunized humanized HLA-A*11:01 mice twice, three weeks apart, with a mix of these 10 peptides, and as a comparator, 1 peptide predicted to bind poorly to HLA-A*11:01 (HLAthena Msi rank > 2) (Table S6). Three ERV-derived peptides, predicted as the best HLA-A*11:01 binders (including the KLIAGLIFLK peptide), evoked T splenocyte responses. These responses were comparable to the responses seen against the Childs’ peptide and the 786-O ERV-derived peptide with the best predicted HLA-A*11:01 binding (Figures 6G–6I and Table S6). This latter peptide was not identified in our tumor HLA MS dataset using our automated filters, but was detected on manual inspection of the data for ccRCC patient 110 (Figure S5E). Twenty-two is thus a conservative estimate of the number of HIF-responsive, ERV-derived peptides in the 11 tumors. As expected, the peptide (NLLSYLGKK) predicted to bind poorly to HLA-A*11 did not elicit a response (Figure 6I and Table S6). Therefore, multiple ERV-derived peptides on human ccRCCs are potentially immunogenic.

**Figure 7.**
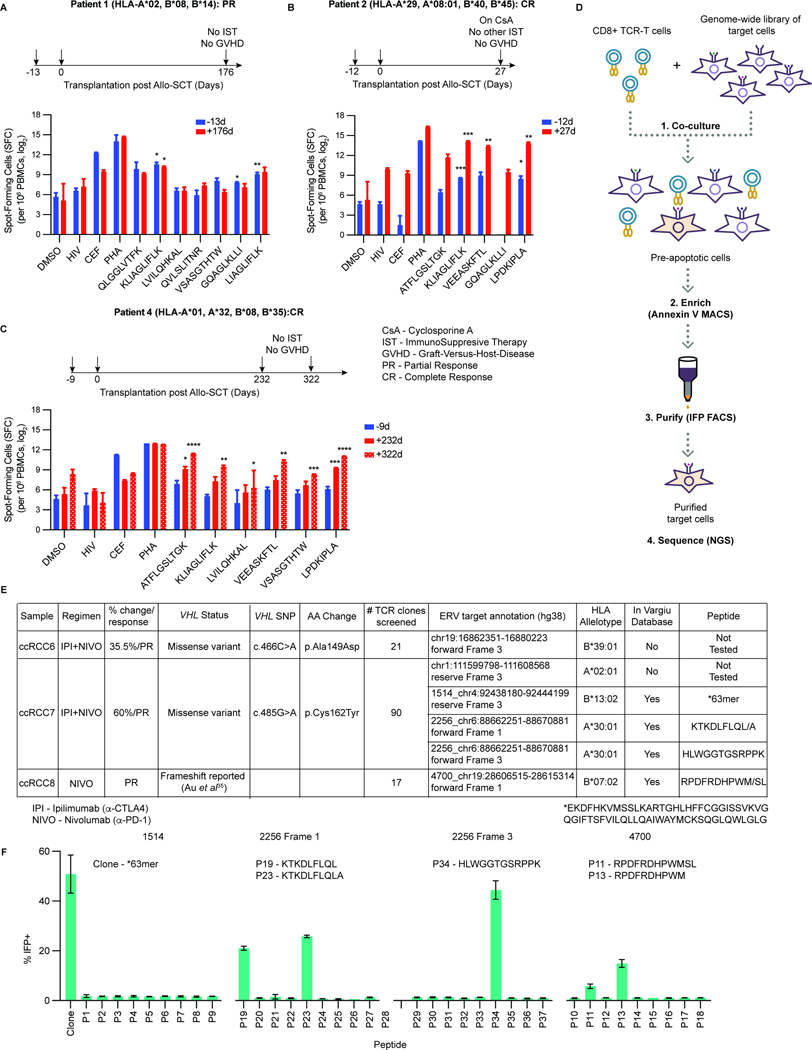
ERV-derived Peptides Recognized by T cells from Human ccRCC Patients (A-C) ELISpot quantification of IFNγ secreting PBMCs derived from ccRCC Allo-SCT patients after stimulation with the indicated ERV-derived peptides. DMSO and an HIV peptide served as negative controls. CEF peptides and PHA served as positive controls. (N=3) Significance was determined by comparing the response to indicated peptides and to the HIV peptide for the corresponding patient. IST(immunosuppressive therapy); GVHD(graft-versus-host-disease); CsA(Cyclosporine A). (D) Schema for T-Scan antigen discovery screen, adapted from Ferretti *et al*^93^. MACS(magnetic activated cell sorting); IFP(infrared fluorescent protein); FACS (fluorescence activated cell sorting); NGS(next generation sequencing). (E) Patient samples with ERV-reactive TILs. (F) Epitope mapping of ERV antigens. Reporter activity (% IFP) of HEK293T reporter cells that were either pulsed with the indicated peptides or transduced with the target protein fragment clone (for 1514 only; 63AA) and co-cultured with the relevant TCR. Sequences for scoring peptides are shown.

Finally, we also obtained archival cryopreserved peripheral blood mononuclear cells (PBMCs) from 6 kidney cancer patients who responded [2 CR and 4 partial response (PR)] to allo-SCT performed ~20 years ago^37,38^ (Figure S6A). We tested samples obtained before and after allo-SCT for their responsiveness in ELISpot assays to the 22 HLA-bound ERV-derived peptides we identified in the 11 kidney tumors (Figure 6B), the 2 HLA-bound ERV-derived peptides we identified in the 786-O cells, the original ERVE-4-derived peptide (ATFLGSLTWK) described by Childs and coworkers^38^, and control peptides. We selected multiple peptides per patient that were predicted to be HLA-binders (HLAthena Msi rank <0.5 or MHC allele rank <0.5) based on their inferred HLA allelotype. The number of viable cells after thawing, and their responsiveness to positive control peptides and to phytohemagglutinin (PHA), were highly variable, potentially confounding formal intrapatient and interpatient comparisons. We excluded one patient’s PBMC samples due to their poor response to the positive controls. In 3 of the remaining 5 patients, we detected reactivity against multiple ERV-derived peptides pre- and post-transplant (Figures 7A–7C and S6B–S6F). In one PR patient we observed reactivity against 3 out of 7 ERV-derived peptides, even before allo-SCT, suggestive of a host immune response (Figures 7A and S6B). In the two CR patients, the number of ERV-derived peptides recognized by the PBMCs increased after allo-SCT, consistent with an enhanced immune response (baseline range: 0–2 peptides; post-transplant range: 3–6 peptides) (Figures 7B, 7C, S6C and S6D). The apparent increase in the number of ERV-derived peptides recognized by the T cells, as well as the apparent increase in ELISpot positive cells per peptide, in the two CR patients is provocative, but requires further study for the reasons stated above.

In a complementary set of experiments, we used the T-Scan platform^62^ to identify potential epitopes recognized by TILs from 10 ccRCC patients who responded to ICIs (Figure 7D). No ERV-derived peptides evoked a response for the TILs from tumors with wild-type (3 patients) or indeterminate (3 patients) *VHL* status (Figure 7E). In contrast, multiple ERV-derived peptides were identified as potentially immunogenic for 3 of the 4 *VHL* mutant tumors, including 2 peptides derived from ERVE-4 and 1 peptide from ERV4700 (Figures 7E, 7F and Table S7). One peptide was derived from ERV1514, which did not originally score as HIF2-responsive in 786-O and A-498 cells. Prompted by this, we discovered that ERV1514 expression was, however, downregulated by PT2399 in 4/10 ccRCC lines (Figure S7). These observations strengthen the conclusion that HIF-responsive, ERV-derived peptides displayed on the surface of ccRCC cells can be immunogenic.

## Discussion

We discovered that HIF transcriptionally regulates a subset of relatively intact proviral ERVs that extends beyond ERVE-4, including ERVs previously linked to the likelihood of ccRCCs responding to ICIs and ERVs that can, like ERVE-4, give rise to HLA-bound peptides that can be effectively recognized by CD8+ T cells. Moreover, we showed that ERV expression can be pharmacologically induced with a clinical grade HIF stabilizer or repressed by a clinical grade HIF2 inhibitor. Our findings might facilitate the discovery of, and therapeutic exploitation of, additional ERV-derived HLA-bound peptides in tumors in the future, as described below.

Our findings are consistent with earlier reports that hypoxia mimetics such as Cobalt Chloride and Dimethyoxylglycine can induce the expression of certain ERVs^63–65^. Hypoxia and hypoxia-mimetics can, however, affect transcription in HIF-independent ways, such as by inhibiting histone demethylases^66,67^. In these prior studies, the specific role of HIF was not addressed or, in the case of HTLV-1 activation by hypoxia, was irrelevant^63^.

Inactivation of the ccRCC tumor suppressor gene *PBRM1* has been found in some, but not all, studies to predict for response to ICIs^10,68,69^. Consistent with our findings, *PBRM1 loss*, which amplifies HIF transcriptional activity in ccRCC^70,71^, increases ERV expression^72^. Therefore *PBRM1* loss might increase the activity of ICIs by increasing ERV expression.

Transcriptional activation of potentially HIF-responsive ERVs likely requires a permissive epigenetic environment, as illustrated by our finding that activation of ERV4700 by HIF2 in 786-O cells required treatment with a DNA methyltransferase inhibitor, in contrast to A-498 cells. More broadly, the fact that DNA is globally hypomethylated in cancer versus normal tissue^28,73^ might render cancer cells more permissive for HIF-induced ERV expression and create a therapeutic window for therapies based on differential ERV expression. ccRCCs might be particularly prone to ERV expression because chronic HIF activation drives the expression of TET enzymes, which promote DNA demethylation, and JmjC enzymes, which promote histone demethylation and a reciprocal increase in histone acetylation^67,74–76^. Notably, decreased histone methylation and increased histone acetylation has been linked to increased ERV expression in gliomas bearing oncohistone mutations^31^.

In addition to activating adaptive immune responses through presentation of ERV-derived peptides on HLA class I molecules or the direct expression of ERV components on the cell surface^26,27,29,33,38,39,77–81^, the expression of ERVs can support innate immune activation, acting through viral mimicry to enhance interferon signaling^82,83^. We have not observed consistent effects of pVHL and HIF on interferon signaling in our isogenic ccRCC cells, although earlier studies found that HIF could induce viral mimicry in ccRCC cells treated with DNA methyltransferase inhibitors^35,84^. The work of Childs and coworkers proves that an ERV-derived peptide can elicit a robust antitumor immune response, as did studies that identified an ERV-derived peptide as the basis for antitumor immunity in the CT26 syngeneic colorectal model in mice and an HERV-K-MEL-derived peptide as the basis for T cell recognition of a human melanoma^78,85^. A recent study linked the response of lung cancers to ICI to the development of anti-ERV antibodies, implying that B cell responses against ERV-derived peptides also contribute to anticancer immunity^86^.

HIF stabilizers might enhance ERV expression and antitumor immunity, while HIF2 antagonists might have the opposite effect (at least for tumors that express HIF2). A caveat is that our knowledge of the effects of HIF on the immune system is incomplete. Moreover, HIF regulates many tumor-intrinsic genes that could be either anti-immunogenic or pro-immunogenic, making the net outcome of HIF on tumor-intrinsic immunogenicity difficult to predict^87^.

Siebenthall and coworkers identified a kidney-specific HIF-binding site for *POU5F1* (encoding *Oct4*) that was encoded by the LTR elements of a subfamily of ERVs^88^. Using publicly available ChIP-seq data, they found that 178 out of 2000 HIF-bound DNA hypersensitivity sites overlapped an LTR element, especially of the ERV1 and ERVK types. They also identified numerous transcriptionally active LTRs, especially for the ERV1 class, and hypothesized that this represented a form of exaptation wherein LTRs are used to drive endogenous genes, with *POU5F1* being but one example. Similarly, LTRs can drive cancer promoting genes, a process referred to as oncoexaptation^25^. Our study suggests that some ERVs are alternatively, or in addition, producing ERV-derived transcripts that might contribute to the immunogenicity of ccRCC and be harnessed therapeutically.

The relationship between ERV expression and immunogenicity is likely to be nuanced, potentially influenced by which specific ERVs are transcribed and translated, by which cells (cancer vs normal) are expressing them, as well as by the patient’s HLA allelotypes. In this regard, our analysis of multiple ccRCC lines and tumors suggests we have not yet reached saturation with respect to potential HIF-responsive ERVs, and we have only explored a limited number of HLA allelotypes for ERV-derived peptides. This could be addressed studying additional *VHL*^*−/−*^ ccRCC lines and tumors, as well as by performing HLA-MS on ccRCC lines engineered to express common HLA variants. These efforts could be complemented by unbiased searches for ERV-derived epitopes and reactive TCRs from PBMCs or TILs from ccRCC patients responsive to immunotherapy. While this work was in progress, Kobayashi and coworkers showed that one of the 5 ERVs we identified as potentially HIF2-responsive and translated, ERV3895, was highly expressed in 3 ccRCCs and gave rise to an immunogenic peptide bound to HLA-A*24, which is an allelotype we did not interrogate^81^. We envision an eventual “look up” table for likely HLA-bound ERV-derived peptides for all common HLA allelotypes. This information could be useful for designing active immunotherapies, such as ERV-targeting therapeutic cancer vaccines, and adoptive TCR-based immunotherapies, such as those based on autologous or allogeneic T cells expressing heterologous ERV-reactive TCRs or on soluble TCR mimetics such as ImmTACs and TCR mimetic antibodies^89,90^.

### Limitations of Study

It is difficult to determine the source of an ERV-derived peptide when it can be derived from multiple genomic loci. Some of this ambiguity might be eliminated through further technical advances in ERV RNA-seq and Ribo-seq. Given the exquisite sensitivity of TCRs for some TCR epitopes^91^, it is possible that we failed to detect some potentially immunogenic ERV-derived peptides because of the current sensitivity limit of MS, which is likely to improve over time.

Many HIF2-responsive ERVs were undetectable by scRNA-seq. This might be a byproduct of the standard 10× Genomics 3’end pipeline, which specifically captures only the 3’ end of polyadenylated transcripts (and not full transcripts or non-polyadenylated ERV transcripts), further limiting sensitivity. Further, there is substantial technical dropout of transcripts in droplet-based scRNA-seq because of the very low quantities of RNA within an individual cell.

We had access to a limited number of PBMC samples from ccRCC patients who responded to allo-SCT and a limited number of TIL samples from ccRCC patients who responded to ICIs. For the former, we also encountered challenges associated with the use of frozen, archival samples. Finally, it is difficult to predict, *a priori*, how partitioning of ERV-reactive T cells between the peripheral blood and tumor will change in response to immunotherapy over time. Clearly additional studies, including additional samples and additional control peptides, will be needed to determine whether peripheral blood T cells capable of recognizing ERV-derived peptides can be used as predictive biomarkers, response biomarkers, or both, for various forms of cancer immunotherapy and whether such peptides can be harnessed for immunotherapy.

### Resource availability

#### Lead contact

Requests for information and resources should be directed to the lead contact, William G Kaelin Jr. (William_Kaelin@dfci.harvard.edu).

#### Materials availability

One plasmid generated in this study has been deposited to Addgene. All unique/stable cell lines generated in this study are available from the lead contact.

#### Data and code availability

- The high-throughput sequencing data have been deposited to NCBI GEO and accession numbers are listed in the key resources table. The original mass spectra for RCC tumor samples 101, 102, 104, and 105 were deposited in the public proteomics repository MassIVE (http://massive.ucsd.edu) and are accessible at ftp://massive.ucsd.edu/v01/MSV000087743/. The original mass spectra for samples newly reported here, as well as peptide spectrum matches, and protein sequence databases used for searches of all samples described here were deposited at MassIVE (https://massive.ucsd.edu) and are accessible at ftp://MSV000096406@massive.ucsd.edu with username: MSV000096406 and password: HIFresponsiveERV. This dataset is publicaly avaialbe as of the date of publication. T-Scan screening data and amino acid sequences for the peptidome library are available upon request. T cell receptor sequences for the purposes of therapeutic development are available through license agreement.
- All the custom scripts used to process the PRO-seq data described in the manuscript are available on the AdelmanLab GitHub (https://github.com/AdelmanLab/NIH_scripts; https://zenodo.org/records/5519915). New code for proteomics data analysis motivated by this project was written to 1) revise the SM sequence database utilities for ERV-specific aspects of 6-frame translation of nucleotide to protein sequence to create the sequence databases used for searches; 2) revise the SM Subset-specific FDR filtering module with ERV-specific features. This new code is included in SM v8.02 and is available free-of-charge at proteomics.broadinstitute.org. Parts of code from Yu *et al*^94^ were adapted into our analysis for scATAC-seq and scRNA-seq datasets, which is accessible at https://github.com/kaelinlabdfci/HIF2_ERVs_in_ccRCC.

## STAR Methods

### Experimental model and study participant details

#### Cell lines

786-O, A-498, 769-P, Caki-2, A-704, HEK293T, and BT-549 cells were originally obtained from the American Type Culture Collection. RCC4 cells were provided by Dr. Peter Ratcliffe (University of Oxford and Francis Crick Institute). OS-RC-2 and TUHR4TKB and TUHR14TKB cells were obtained from RIKEN Bioresource Research Center Cell Bank. UM-RC-2 were provided by Bert Zbar and Marston Linehan (National Cancer Institute). WM266–4 cells were provided by Dr. Rizwan Haq (Dana-Farber Cancer Institute) and Dr. David E. Fisher (Massachusetts General Hospital). MGG152 cells^95^ were provided by Dr. Samuel McBrayer (UT Southwestern Medical Center). All the cells were used in accord with the terms which they were obtained. 786-O, A-498, 769-P, RCC4, HEK293T and TUHR4TKB cells were cultured in DMEM (Gibco 11995073). Caki-2 cells were cultured in McCoy’s 5A (Corning 10050CV). BT-549, WM266–4, OS-RC-2 and TUHR14TKB cells were cultured in RPMI-1640 (Gibco 11875119). All media were supplemented with 10% FBS (GeminiBio 100–106) and 1% penicillin-streptomycin (Gibco 15140122). A-704 was cultured in EMEM (Corning MT10010CM) supplemented with 2 mM glutamine (Gibco 25030081), 1× non-essential amino acids (VWR 45000–700), 1 mM sodium pyruvate (Gibco 11360070), 15% FBS and 1% penicillin-streptomycin. MGG152 was cultured in Neurobasal Medium (Gibco 21103049) supplemented with 3 mM glutamine, 1× B-27 supplement (Gibco 17504044), 0.25× N-2 supplement (Gibco 17502048), 20 ng/mL human recombinant EGF (STEMCELL Technologies 78006.1), 20 ng/mL bFGF (STEMCELL Technologies 78003.1), 2 μg/mL heparin solution (STEMCELL Technologies 07890), 1% penicillin-streptomycin, and 125 ng/mL Amphotericin B (Thermo Fisher Scientific SV3007801). Cells were routinely tested for mycoplasma using MycoAlert Mycoplasma Detection Kit (Lonza LT07–318). Cells were maintained at 37°C in an atmosphere containing 5% CO_2_.

#### Animals

6–8 week old, female HLA-A*11:01 transgenic mice were purchased from Taconic [Taconic CB6F1-Tg(HLA-A*1101/H2-Kb)A11.01]. The mice carry a transgene consisting of fragment of the human HLA-A*11:01 gene and mouse H2-K^b^ gene. Animals were maintained in the animal facility at Dana-Farber Cancer Institute in compliance with the Institutional Animal Care and Use Committee.

#### RCC patients enrolled in NeoVax trial

Human RCC and adjacent non-malignant kidney biospecimens were collected as part of a clinical trial (NCT02950766, NeoVax) of personalized neoantigen vaccination as adjuvant therapy for high-risk, resected RCC. The clinical information about each patient is listed as follows:

**Table:** 

ID	Sex	Age at Nephrectomy	Stage
16097_101	Male	58	III
16097_102	Female	57.3	III
16097_104	Female	57.6	IV NED
16097_105	Male	65.6	III
16097_106	Male	72.9	III
16097_107	Male	75.8	III
16097_108	Male	66.4	IV NED
16097_109	Male	50.5	III
16097_110	Male	65.3	III
16097_111	Male	65	III
16097_112	Female	55.6	III

#### RCC patients enrolled in allo-SCT study

PBMCs were collected as part of a clinical trial, from human RCC patients, who underwent allo-SCT at National Heart, Lung, and Blood Institute (NHLBI)^37,38^. The clinical information about each patient is listed as follows:

**Table:** 

ID	Sex	Age at Transplant	Tumor Stage	Sex Matched Donor
NHLBI-RCC-39	Female	46	Metastatic	Yes
NHLBI-RCC-1	Male	50	Metastatic	Yes
NHLBI-RCC-69	Male	52	Metastatic	No
NHLBI-RCC-62	Female	57	Metastatic	Yes
NHLBI-RCC-63	Male	60	Metastatic	No
NHLBI-RCC-71	Male	57	Metastatic	Yes

#### RCC patients enrolled in T-scan screening

The study was conducted in accordance with the Declaration of Helsinki (1996), approved by the Washington University School of Medicine Institutional Review Board (IRB) HRPO 201411135. To be eligible for the study, patients were required to have a known or suspected diagnosis of ccRCC, to have received treatment with ICI therapy, and to have undergone surgical resection of residual tumors following a partial response by RECIST 1.1 or irRECIST criteria of at least one of the resected lesions.

Seven eligible patients were identified by the participating site through direct contact. Case report forms did not contain identifying information. Samples were de-identified at the participating site with an anonymous code assigned to each sample. Anonymized dissociated tumor tissue samples from surgical resections were sent to TScan Therapeutics laboratories with limited demographic and clinical data. Demographics included age and sex. Clinical data included dates of diagnosis, pre- and post-treatment imaging CT scans and the prescribed treatment regimen prior to nephrectomy. Samples ccRCC1-ccRCC6 were all derived from post-treatment nephrectomies. Sample ccRCC7 comprised 5 dissociated tissue samples derived from a pre-treatment nephrectomy as well as post-treatment resections from 2 metastatic lesions in lung and vagina. Sample ccRCC7 was the only sample for which pre-treatment tissue was obtained. For three samples (ccRCC8-ccRCC10), single-cell TCR data were obtained from published data sets^59,96^ (patient ADR013 was relabeled as ccRCC8). The clinical information about each patient is listed as follows:

**Table:** 

ID	Sex	Age	Stage	Regimen Prior to Nephrectomy	Percent change/response
TScan-ccRCC1	Male	66	IV	IPI+NIVO	39%/PR
TScan-ccRCC2	Male	75	IV	IPI+NIVO	37.5%/PR
TScan-ccRCC3	Male	56	IV	AXI+PEM	45%/PR
TScan-ccRCC4	Male	51	IV	AXI+PEM	41%/PR
TScan-ccRCC5	Female	61	IV	IPI+NIVO+CABO	42%/PR
TScan-ccRCC6	Female	50	IV	IPI+NIVO	35.5%/PR
TScan-ccRCC7	Female	49	IV	Nephrectomy followed by IPI+NIVO for metastases	60%/PR
Au13-ccRCC8	N/A	N/A	N/A	NIVO	PFS>10 month/PR
T3-ccRCC9	Male	64	IV	IPI+NIVO	Mixed response
T40ccRCC10	Male	63	IV	IPI+NIVO	CR

Note: IPI-Ipilimumab (CTLA4); NIVO-Nivolumab (PD-1); PEM-Pembrolizumab (PD-1); CABO-Cabozantinib (VEGFR); AXI-Axitinib (VEGFR)

### Method Details

#### Lentiviral vectors

The pLenti-EF1α-Cas9-FLAG-IRES-Neo vector^97^ was used to generate Cas9-expressing cells. The pLX304-EV-IRES-GFP (Addgene 187640) and pLX304-VHL-IRES-Tdtomato (Addgene 187643) vectors were used to generate isogenic 786-O, A-498 and RCC4 cell lines. pLenti-EF1α-HIF2α dPA (P405A, P531A) vector (Addgene 232954) was used to generate 786-O cells with overexpressed HIF2α mutant.

The pLentiCRISPRv2-puro vector (Addgene 98290) was used as a backbone for all sgRNA expression vectors. The vector was digested with FastDigest Esp3l (Thermo Fisher Scientific FD0454) for 15 minutes (min) at 37°C, and the linearized vector was gel-purified using QIAquick Gel Extraction Kit (Qiagen 28706). sgRNA sense and antisense oligonucleotides were mixed at an equimolar ratio, phosphorylated by T4 Polynucleotide Kinase (New England Biolabs M0201L), and annealed. The mixture was incubated for 30 min at 37°C, for 5 min at 95°C and cooled to 25°C at a rate of 5°C/min in a thermocycler. The PCR products were diluted at 1:200 and then ligated with purified linearized vector using T4 DNA ligase (New England Biolabs M0202L) at 25°C for 1 hour (h). The ligation product was transformed into chemically competent HB101 *E. Coli* (Promega L2011) at 1:10 (v/v). Plasmids from ampicillin-resistant colonies were purified by QIAprep Spin Plasmid Miniprep Kit (Qiagen 27106) and validated by sanger sequencing.

#### Lentivirus production and infection

HEK293T cells were transfected with the lentiviral expression vector and the packaging vectors psPAX2 (Addgene 12260) and pMD2.G (Addgene 12259) in a 4:3:1 ratio using Lipofectamine 2000 (Invitrogen 11668019). The media was removed 24 h later and replaced with fresh media. The virus-containing supernatant was collected at 48 h and 72 h post transfection, pooled, and purified with 0.45-μm SCFA filters (Corning 431220). Viruses were then aliquoted and frozen at −80°C.

The adherent cells to be transfected were trypsinized and resuspended in fresh serum-containing media. 2×10^5^ cells/well in 2.3 mL of media were mixed with 200 μL viral supernatant and polybrene (Santa Cruz Biotechnology sc-134220 at the final concentration of 8 μg/mL) in 6-well plates. Plates were centrifuged at 2,000×*g* for 2 h at room temperature (RT) and then incubated in a humidified chamber overnight at 37°C. The supernatant was then removed and replaced with fresh media for an additional 24 h before antibiotics (puromycin at the final concentration of 2 μg/mL or blasticidin S at the final concentration of 10 μg/mL) were added for selection. For cells that were infected with bicistronic vectors (pLX304-EV-IRES-GFP or pLX304-VHL-IRES-Tdtomato), GFP or Tdtomato positive cells were isolated by FACS.

#### Chemicals

PT2399 was provided by Peloton Therapeutics and dissolved in DMSO to a final concentration of 100 mM for stock solutions. FG-4592 (ApexBio A4187) was dissolved in DMSO to a final concentration of 100 mM for stock solutions. GSK3685032 (DNMT1 inhibitor) was provided by GSK and dissolved in DMSO to prepare 1 mM stock solutions.

#### Immunoblot analysis

Cells were washed with PBS, trypsinized, transferred to Eppendorf tubes and collected by centrifugation at 300×*g* for 5 min at 4°C. The cell pellets were washed once with ice-cold PBS and then lysed in EBC buffer (50 mM Tris-HCl pH 7.4, 150 mM NaCl, 1% Nonident P-40, and 10% glycerol) supplemented with cOmplete protease inhibitors (Roche 11697498001) and PhosSTOP phosphatase inhibitors (Roche 04906837001). Pellets were thoroughly disrupted by repeated pipetting followed by end-over-end rotation for 30 min at 4°C. Lysates were cleared by centrifugation at 17,000×*g* for 15 min at 4°C. Protein lysates concentration was determined by the Bradford assay (Bio-Rad Laboratories 5000006).

Extracts were diluted in Laemmli sample buffer, boiled at 95°C for 5 min, loaded onto 4–20% protein gel (Novex WedgeWell XP04205BOX), resolved by sodium dodecyl sulfate (SDS) polyacrylamide gel electrophoresis, and transferred to nitrocellulose membranes using the Transblot Turbo System (Bio-Rad 1704155). Membranes were first stained with Ponceau S Staining Solution (Cell Signaling Technology 59803) to ensure proper transfer and rinsed with TBS-T (20 mM Tris, 150 mM NaCl, 0.1% Tween-20) on a rocker until the Ponceau S stain was not visible. The membrane was then incubated with 5% non-fat milk in TBS-T for 1 h at RT followed by three washes with TBS-T (5 min/wash). The membrane was incubated with primary antibody diluted in 5% protease-free bovine serum albumin (BSA, GoldBio A-420–10) and 0.05% sodium azide overnight at 4°C. On the following day, the primary antibody was removed, and the membrane was washed three times with TBS-T (5 min/wash) and then incubated with HRP-conjugated secondary antibodies [goat anti-rabbit immunoglobulin G (IgG) (Jackson ImmunoResearch Labs 111–035-003) or goat anti-mouse IgG (Jackson ImmunoResearch Labs 115–035-003)] diluted at 1:3000 for 1 h at RT. Then membrane was washed three times with TBS-T (5 min/wash). Bound antibodies were detected with Pierce ECL Plus Western Blotting Substrate (Thermo Fisher Scientific 32106) or SuperSignal West Pico PLUS Chemiluminescent Substrate (Thermo Fisher Scientific 34578). Chemiluminescent signal was detected with autoradiographic film (Denville E3031) or Azure Biosystems c600 Imager.

The primary antibodies used were rabbit α-HIF2α (Cell Signaling Technology 7096) at 1:1000, α-NDRG1 (Cell Signaling Technology 5196) at 1:1000, rabbit α-VHL (Cell Signaling Technology 68547) at 1:1500, rabbit α-HIF1α (Cell Signaling Technology 14179) at 1:1000, rabbit α-BNIP3 (Cell Signaling Technology 44060) at 1:1000, mouse α-Tubulin (Sigma-Aldrich T5168) at 1:10,000, SETDB1 (Proteintech 11231–1-AP) at 1:1000, rabbit α-IRF-7 (Cell Signaling Technology 4920) at 1:1000, rabbit α-Rig-I (Cell Signaling Technology 4200) at 1:1000.

#### Reverse transcription quantitative PCR (RT-qPCR)

Cells were homogenized with QIAshredder columns (Qiagen 79654), and total RNA was extracted from homogenized cell lysates using the RNeasy Mini Kit (Qiagen 74106) according to manufacturer’s protocol. The RNA was quantified on a NanoDrop spectrophotometer (Thermo Scientific ND-8000-GL). cDNA was reverse transcribed from 1 μg purified RNA in a 10 μL reaction using the AffinityScript qPCR cDNA Synthesis Kit (Agilent 600559) with random primers as the primer probes. cDNA for each sample was diluted 10-fold for RT-qPCR. RT-qPCR was performed in triplicates on 384-well plates (Roche 047297490001) on a LightCycler 480 Instrument II (Roche). The volume of each reaction was 7 μL [1.8 μL diluted cDNA, 1 μL H_2_O, 0.7 μL primer mix (forward and reverse primers; both at the concentration of 5 μM) and 3.5 μl SYBR Green I Master Mix (Roche 04707516001)]. Relative cDNA amount was calculated based on 2^-ΔΔCt^ values using *ACTB* as a reference.

#### RNA-seq sample library preparation and sequencing

For RNA-seq experiments to identify HIF-response ERVs in 786-O (QJ8080) and A-498 cells (QJ9511), ribosomal RNA (rRNA)-depleted libraries were prepared using KAPA RNA HyperPrep Kit with RiboErase (Roche) from 100 ng of total RNA according to the manufacturer’s protocol. Briefly, mRNA or non-coding RNA were enriched and then purified using KAPA pure beads and fragmented at 94°C for 8 min. 1^st^ strand and 2^nd^ strand were subsequently synthesized. cDNA fragments were end-repaired and adenylated at the 3′ ends, and universal adapters were ligated to cDNA fragments, followed by index addition and library enrichment with 14 cycles of PCR post-adapter ligation on a Beckman Coulter Biomek i7. RNA-seq experiments performed to identify ERVs whose transcription become dependent on HIF2α upon GSK3685032 (DNMT1 inhibitor) treatment (QJ10472) were done in the same manner except that the rRNA depletion was performed using QIAseq FastSelect rRNA HMR reagents (Qiagen 334375) according to the manufacturer’s protocol. Finished dsDNA libraries were quantified by Qubit fluorometer and Agilent TapeStation 4200. Uniquely dual indexed libraries were pooled in an equimolar ratio and shallowly sequenced on an Illumina MiSeq to further evaluate library quality and pool balance. The final pool was sequenced with paired-end 150 bp reads on an Illumina NovaSeq 6000 at the Dana-Farber Cancer Institute Molecular Biology Core Facilities.

**Table:** 

Experiment Identifier	Description of Experiments and Data Location in Figures/Tables
QJ8080	Identify HIF-responsive ERVs in 786-O cells, Figure 2, S1 and Table S1
QJ9511	Identify HIF-responsive ERVs in A-498 cells, Figure S1 and Table S1
QJ10472	Identify ERVs whose transcription become dependent on HIF2α upon DNMT1 inhibitor treatment in 786-O cells, Figure S2 and Table S1

#### ERV quantification based on RNA-seq

Human ERV RNA levels were quantified using a previously published method^10^. Briefly, raw sequencing reads were first aligned to the reference human transcriptome (hg19) using bowtie2 (v2.3.4.3)^98^. Reads that failed to map to the human transcriptome were subsequently aligned to the reference ERV genome^41^. Paired-end reads where both ends perfectly matched to at least one ERV reference sequence (PM), or those with no more than one mismatch across both reads (1MM), were preserved. Single ends from read pairs that met the following criteria were also kept for downstream analysis: (i) perfect match to an ERV reference sequence; (ii) not part of any paired-end PM or 1MM matches to any ERV reference sequence; (iii) no matches to the human transcriptome. Duplicate reads were identified and removed using Picard MarkDuplicates [Picard Toolkit, Broad Institute, http://broadinstitute.github.io/picard/(2021)]. Quantification was performed using HTSeq (v0.11.0)^99^, and the final expression was defined as 2× number of paired-end matches (PM & 1MM) + number of single-end matches to the ERV reference database. The expression of each ERV was normalized to the library size of the corresponding sample as well as the length of the ERV. These normalized ERV counts were used in downstream analyses.

#### HIF signature correlation with ERV

We leveraged available RNA-seq data from 250 tumors from patients with advanced ccRCC on the CheckMate 025 trial. The previously generated TPM matrix and normalized ERV raw counts as described in Braun *et al*^10^ were used. Expression data were log-transformed and z-score normalized. A validated HIF2 signature^43^ and a housekeeping gene signature^92^ were calculated by averaging the normalized expression data of each gene set. Pearson’s correlations were computed between the expression levels of 15 ERVs and expression of the HIF2 and housekeeping gene signatures, as well as the HIF2-dependent genes *EGLN3* and *NDRG1*. P-values were adjusted for multiple comparisons using the Benjamini–Hochberg method for each gene/pathway.

#### Knock-In FLAG-HA tag at the endogenous HIF2α locus

Solid Phase Reversible Immobilization (SPRI) magnetic beads were prepared by washing 1 mL carboxylate-modified magnetic bead solution (GE Healthcare 65152105050250) three times with 1 mL TE buffer (10 mM Tris-HCl pH 8.0, 1 mM EDTA pH 8.0) using a magnetic stand (Invitrogen 12321D). The beads were then resuspended in 50 mL DNA precipitation buffer [10 mM Tris-HCl pH 8.0, 1 mM EDTA pH 8.0, 1 M NaCl, 18% w/v PEG8000 (Sigma-Aldrich 89510–250G-F), 0.05% (v/v) Tween20] and stored at 4°C. For each experiment, an aliquot of the beads was equilibrated to RT before use. A double-stranded homology directed repair (HDR) DNA template was prepared using a previously published method^100^ with modifications. A double-stranded DNA that was designed to introduce a 3× FLAG-HA epitope tag and intervening linker to the N-terminus of HIF2α and which was flanked by 5’ and 3’ homology arms was synthesized by Genewiz and provided in a pUC-Genewiz-Amp plasmid. This plasmid was used as a template for high-output PCR amplification using Kapa HotStart polymerase (Roche KK2601). To purify the resulting PCR product, 100 μL of the PCR reaction was mixed with 120 μL of SPRI magnetic beads in an Eppendorf tube and the mixture was incubated for 10 min at RT. The Eppendorf tube was then placed on the magnetic stand for several minutes so that the beads would attach to the side of the Eppendorf tube. Next, the liquid was removed, and the beads were washed twice with 1 mL of freshly prepared 70% ethanol while leaving the tube in the stand. After the final wash, the liquid was removed by gentle aspiration and the beads were allowed to air-dry for 5–6 min. The Eppendorf tube was then removed from the magnetic stand and the beads were resuspended in 12 μL of 2 mM Tris-HCl pH 8.0 and incubated for 10 min at RT. The Eppendorf tube was then placed back in the magnetic stand for several minutes to capture the beads and supernatant containing the purified HDR template was transferred to a new Eppendorf tube.

To produce Cas9-Ribonucleoprotein (RNP) complexes, 1.2 μL of synthetic sgRNA (IDT) (stock solution: 100 μM), 1.4 μL of Alt-R Cas9 Electroporation Enhancer (stock solution: 100 μM, IDT 1075916), 1.7 μL of Alt-R S.p. Cas9 Nuclease V3 (stock solution: 62 μM, IDT 1081059), and 0.7 μL PBS (Gibco 14190–144) were mixed and incubated for at least 10 min at RT. Meanwhile, 2×10^5^ cells were resuspended in 20 μL of SF-nucleofection buffer (Lonza Amaxa SF Cell Line 4D-Nucleofector × Kit V4XC-2032) and added to the Cas9-RNP mixture together with 3 μg of HDR template (typically 1.5–2 μg/μL). This final mixture was transferred to Nucleocuvette strips (Lonza Amaxa SF Cell Line 4D-Nucleofector × Kit V4XC-2032). The mixture was electroporated by using 4D-Nucleofector × unit with the electroporation condition EN138 based on pilot experiments in which the electroporation efficiency for the cells to be electroporated was tested for the 15 manufacturer provided electroporation conditions and manufacturer provided GFP reporter plasmid. Then, 100 μL of prewarmed media was added into each cuvette and incubated for 10 min at RT. Individual electroporation mixtures were transferred into individual wells of 6-well plates containing 2 mL/well of prewarmed media supplemented with 1 μM of Alt-R HDR enhancer V2 (stock solution: 0.69 mM, IDT 10007921). The media was removed 16 h later and replaced with fresh media. Knock-in efficiency was determined by amplicon sequencing or immunoblot analysis 3–4 days post nucleofection. Polyclonal 786-O, OS-RC-2 and RCC4 cells with FLAG-HA knock-in at the N-terminus of HIF2α locus were used for the following ChIP-seq experiments. Single cell cloning was conducted for 786-O cells with FLAG-HA knock-in at the N-terminus of HIF2α locus and two clones were used for the following ChIP-qPCR assays.

#### Chromatin Immunoprecipitation (ChIP)

For ChIP sample preparation, 1×10^7^ adherent cells were washed with PBS, trypsinized, transferred to Eppendorf tubes and collected by centrifugation at 300×*g* for 5 min at RT. Cell pellets were washed once with RT PBS-complete [PBS supplemented with 1× protease inhibitor (Sigma-Aldrich PIC0006) and 5 mM sodium butyrate], resuspended in 2 mM of disuccinimidyl glutarate (DSG, Thermo Fisher Scientific 20593) diluted in PBS, and then agitated at 850 rpm on a Fisherbrand Isotemp Heat/Cool Programmable Thermal Mixer II (Thermo Fisher Scientific) at RT for 45 min. The cell pellets were collected by centrifugation at 300×*g* for 5 min at RT. The supernatant was removed, and the cell pellets were resuspended in freshly made 1% formaldehyde (Thermo Fisher Scientific 28908) diluted in PBS followed by agitation at 850 rpm on a Fisherbrand Isotemp Heat/Cool Programmable Thermal Mixer II for 10 min at RT. The crosslinking was quenched by adding glycine to a final concentration of 0.125 M with agitation at 850 rpm on a Fisherbrand Isotemp Heat/Cool Programmable Thermal Mixer II for 5 min at RT. The cell pellets were collected by centrifugation at 300×*g* for 5 min at 4°C. Cell pellets were washed once with ice-cold PBS-complete. Crosslinked cells were then pelleted, snapfrozen, and stored at −80°C.

Aliquots of frozen pellets were thawed on ice, resuspended in 500 μL of 1% SDS lysis buffer (50 mM Tris-HCl pH 8.0, 10 mM EDTA pH 8.0, 1% SDS, 1× protease inhibitor, 5 mM sodium butyrate), and lysed on ice for 10–15 min. The lysates were transferred to 1 mL Adaptive Forced Acoustics (AFA) fiber milliTUBEs (Covaris 520135) and sonicated at the following settings: 140 peak incident power, 5% duty factor and 200 cycles per burst) using Covaris E220 sonicator. Sonication lasted for 10 min (per sample for ChIP-seq samples) or 20 min (per sample for ChIP-qPCR samples). Post sonication, the lysates were transferred to Eppendorf tubes and cleared by centrifugation at 17,000×*g* for 15 min at 4°C.

To determine the amount of precleared chromatin to be used for ChIP-seq experiments, 10 μLl of the lysates were aliquoted (INPUT) and 90 μL of TE was added to make 100 μl solution. 1 μL RNase A (Invitrogen AM2696) was added to the samples and incubated for 30 min at 37°C, then 5 μL Proteinase K (20 mg/ml, Thermo Fisher Scientific EO0492) was directly added to the mixture and incubated at 65°C for 16 h with constant agitation at 850 rpm on a Fisherbrand Isotemp Heat/cool Programmable Thermal Mixer II at the indicated temperatures. Next the chromatin was purified using PCR Purification Kit (Qiagen 28104). Briefly, 500 μL PB buffer was added to each 100 μL sample and the mixture was mixed and loaded onto the purification column. The purified chromatin was eluted using 32 μL EB buffer. Chromatin (INPUT) concentration was determined using a Qubit High Sensitivity Kit (Life Technologies A33231) on a Qubit 4 Fluorometer (Invitrogen Q33239). 40 μg chromatin (INPUT) was immunoprecipitated with 5 μg of FLAG antibody (Sigma-Aldrich F1804) or 10 μL HIF2α antibody (a gift from Dr. David R. Mole)^101^. For ChIP-qPCR experiments, the concentration of precleared cell lysates were determined by the Bradford assay (Bio-Rad Laboratories 5000006). 500 μg of protein lysates were aliquoted and incubated with 2 μg of FLAG antibody for the subsequent immunoprecipitation experiments.

Antibody-bead conjugation was done by first taking 30 μL of Protein G Beads (Life Technologies 10004D) per sample and washing them three times with 500 μL ice-cold 0.5% BSA diluted in PBS. The beads were then resuspended in 500 μL ice-cold 0.5% BSA diluted in PBS with the desired number of antibodies and rotated end-to-end for 4–6 h at 4°C, followed by two additional washed with ice-cold 0.5% BSA diluted in PBS to remove unbound antibodies. Lysates containing the desired amount of chromatin/protein were diluted 10-fold using ChIP dilution buffer (20 mM Tris-HCl pH 8.0, 1% Triton X-100, 2 mM EDTA, 150 mM NaCl, 1× protease inhibitor, 5 mM sodium butyrate), added to pre-washed antibody-bead conjugates and then rotated end-to-end overnight at 4°C. The next day, the bead-bound chromatin-antibody complexes were washed twice with RIPA 0 buffer (10 mM Tris-HCl pH 7.4, 1 mM EDTA pH 8.0, 0.1% SDS, 1% Triton X-100, 0.1% sodium deoxycholate), twice with RIPA 0.3 buffer (10 mM Tris-HCl pH 7.4, 1 mM EDTA pH 8.0, 0.1% SDS, 1% Triton X-100, 0.1% sodium deoxycholate, 0.3 M NaCl), twice with LiCl buffer (10 mM Tris-HCl pH 7.4, 1 mM EDTA pH 8.0, 0.5% NP40, 0.5% sodium deoxycholate, 0.25 M LiCl), and then eluted with 100 μl pre-warmed (37°C) ChIP elution buffer (1% SDS, 0.1 M NaHCO_3_ in H_2_O). 1 μl RNase A was added to the samples and incubated for 30 min at 37°C, then 5 μl Proteinase K was added to the samples and incubated at 65°C for 16 h. For RNase A and Proteinase K incubation, the samples were constantly agitated at 850 rpm on Fisherbrand Isotemp Heat/cool Programmable Thermal Mixer II.

For ChIP-seq samples, the supernatant was purified using a MinElute PCR Purification Kit (Qiagen 28004), eluted in 12 μL H_2_O, and quantified using a Qubit High Sensitivity Kit. A 5 μL sample was used for ChIP-seq library preparation with a Swift DNA Library Prep Kit (Swift Biosciences). 75 bp paired-end reads were sequenced on an Illumina NextSeq 500 instrument. Browser snapshots shown in Figure 3 and S2 were generated with bigWig files and Integrative Genomics Viewer (https://software.broadinstitute.org/software/igv/). For ChIP-qPCR samples, chromatin was purified using a PCR Purification Kit (Qiagen 28104). The purified chromatin was eluted with 50 μl H_2_O. The eluted samples were diluted 2-fold with H_2_O prior to RT-qPCR. RT-qPCR was performed in triplicates in 384-well plates with a LightCycler^®^ 480 Instrument II. The volume of each reaction was 7 μL [1.8 μL diluted DNA, 1 μL H_2_O, 0.7 μL primer mix (forward and reverse primers; both at the concentration of 5 μM) and 3.5 μL SYBR Green I Master Mix]. Relative genomic DNA amount was calculated based on 2^-ΔΔCt^ values using *ACTB* as a reference.

For peak calling and data analysis, all samples were processed through the computational pipeline developed at the Dana-Farber Cancer Institute Center for Functional Cancer Epigenetics (CFCE) using primarily open-source programs^102,103^. Sequence tags were aligned with Burrows-Wheeler Aligner (BWA)^104^ to build hg19 and uniquely mapped, non-redundant reads were retained. These reads were used to generate binding sites with Model-Based Analysis of ChIP-seq 2 (MACS v2.1.1.20160309), with a *q*-value (FDR) threshold of 0.01^105^. We evaluated multiple quality control criteria based on alignment information and peak quality: (i) sequence quality score; (ii) uniquely mappable reads (reads that could only map to one location in the genome); (iii) uniquely mappable locations (locations that could only be mapped by at least one read); (iv) peak overlap with Velcro regions, a comprehensive set of locations – also called consensus signal artifact regions – in the genome that have anomalous, unstructured high signal or read counts in next-generation sequencing experiments independent of cell line and of type of experiment; (v) number of total peaks (each sample had >3,000 peaks, passing the minimum requirement of 1000); (vi) high-confidence peaks (the number of peaks that were 10-fold enriched over background); (vii) percentage overlap with known DHS sites derived from the ENCODE Project (samples meet the minimum threshold 80%); (viii) peak conservation (a measure of sequence similarity across species based on the hypothesis that conserved sequences are more likely to be functional). All samples in this study passed quality control criteria.

For differential binding analyses, differential peak analysis was performed using the CoBRA pipeline^103^. Differential peaks were identified by DEseq2 with adjusted *p* ≤ 0.05. A total number of reads in each sample was applied to the size factor in DEseq2, which can normalize the sequencing depth between samples.

To identify overlapped ERV and HIF2α peak location, the HIF2α bed file was extended by 1 kb, 5 kb, 10 kb for each peak region using the BEDOPS tool^106^. The extended HIF2α bed files were intersected with the ERV reference bed file using the BedTools intersect command^107^. Final lists contain overlapped genomic locations of ERV and extended HIF2α peaks, which are listed in Table S2.

#### PRO-seq sample preparation and analysis

Cells (Human or *Drosophila*) were washed with RT PBS, trypsinized for 1–2 min at 37°C, quenched with ice-cold DMEM +10% FBS and transferred to 50 mL conical tubes. After harvesting, cells were always kept on ice. 5×10^6^ to 1×10^7^ cells per condition were collected by centrifugation at 300×*g* for 4 min at 4°C. The supernatant was removed, and the cell pellets were gently resuspended in 10 mL ice-cold PBS, followed by centrifugation at 300×*g* for 4 min at 4°C. The supernatant was removed, and the cells were resuspended in 250 μL wash buffer [10 mM Tris-HCl pH 8.0, 10 mM KCl, 250 mM sucrose, 5 mM MgCl_2_, 1 mM EGTA, 10% (v/v) glycerol, 0.5 mM DTT (Thermo Fisher Scientific 707265ML), 0.2 μL/mL RNase inhibitor (Invitrogen AM2696) and 1× protease inhibitor (Thermo Fisher Scientific A32965)]. The cell pellets were gently pipetted using wide bore filtered pipette tips (Thermo Fisher Scientific 2079GPK) to create single-cell suspensions. The cell suspension was passed through a cell strainer once (Falcon 352235) to avoid cell clumps. 10 mL permeabilization buffer [10 mM Tris-HCl pH 8.0, 10 mM KCl, 250 mM sucrose, 5 mM MgCl_2_, 1 mM EGTA, 10% (v/v) glycerol, 0.1 % (v/v) IGEPAL CA-630, 0.05% (v/v) Tween-20, 0.5 mM DTT, 0.2 μL/mL RNase inhibitor and 1× protease inhibitor] was slowly (over 5–10 seconds) added to the single-cell suspension along the wall of the conical tube. The samples were then incubated on ice for 5 min before centrifugation at 400×*g* for 4 min at 4°C. The supernatant was removed by gently inverting the tubes after which 1 mL of ice-cold wash buffer was added to each of the pellets to dilute out the residual permeabilization buffer followed by gently pipetting 2–3 times to achieve a single cell suspension. Next an additional 9 mL of ice-cold wash buffer was added to each tube and the samples were pelleted by centrifugation at 400×*g* for 4 min at 4°C. This wash procedure (1 mL wash buffer followed by 9 mL wash buffer) was repeated an additional 2 times. After the washes, the cell pellets were resuspended in 250 μL freezing buffer [50 mM Tris-HCl, pH 8.0, 40% (v/v) glycerol, 5 mM MgCl_2_, 1.1 mM EDTA, 0.5 mM DTT, 1 μL/mL RNase inhibitor] and transferred to 1.5 mL low binding tubes (Thermo Fisher Scientific 90410). 250 μL freezing buffer/tube was also added to the 50 mL conical tubes, followed by gentle pipetting, to collect any residual cells, which were then pooled with the first resuspension (bringing the total volume to 500 μL, which was further resuspended by gentle pipetting). Trypan blue-stained cell number was determined using a hemocytomer after which the cells were diluted using freezing buffer to the concentration of 1×10^6^ cell/100 μL. Aliquots of 5×10^6^ permeabilized cells per 1.5 mL low binding tube were flash frozen in liquid nitrogen and stored at −80°C prior to use.

To conduct nuclear run-ons and library preparation, aliquots of frozen permeabilized cells were thawed on ice, pipetted gently, and recounted using a Luna II automated cell counter (Logos Biosystems). For each sample, 1×10^6^ permeabilized Human cells were used for nuclear run-ons, with 5×10^4^ permeabilized *Drosophila* S2 cells added to each sample for normalization. Nuclear run-on assays and library preparation were performed as described in Reimer *et al*^56^ with modifications. Briefly, nascent RNA was labeled by a run-on assay in which an equal volume of 2× nuclear run-on buffer [10 mM Tris-HCl, pH 8.0, 300 mM KCl, 1% sarkosyl, 10 mM MgCl_2_, 1 mM DTT, 20 μM/ea biotin-11-NTPs (Perkin Elmer NEL543001EA), 0.8 U/μl RNase inhibitor)] was added into permeabilized cell mixture and incubated for 5 min at 37°C. RNA was purified by the Total RNA Purification Kit (Norgen Biotek Corp 17200) per manufacturer’s instructions, eluted in 100 μL H_2_O, and fragmented by base hydrolysis by adding 25 μL 5× fragmentation solution (375 mM Tris-HCl, pH 8.3, 562.5 mM KCl, 22.5 mM MgCl_2_) followed by incubation for 5 min at 94°C. Fragmentation was stopped by adding 125 μL ice-cold 0.1 M EDTA. To enrich for biotin-labeled nascent RNA, 10 μL of streptavidin C1 magnetic beads (Invitrogen 65001) were washed, rendered RNase free per manufacturer’s instructions, and resuspended in 50 μL binding buffer (10 mM Tris-HCl pH 7.4, 300 mM NaCl, 0.1% Triton X-100) before being added to the fragmented RNA. The samples were rotated end-to-end for 20 min at RT. The streptavidin magnetic beads were then washed twice with high salt buffer (50 mM Tris-HCl pH 7.4, 2 M NaCl, 0.5% Triton X-100), twice with binding buffer (listed above), and twice with low salt buffer (5 mM Tris-HCl pH 7.4, 0.1% Triton X-100). The beads were next resuspended in 500 μL TRIzol Reagent (Invitrogen 15596026) and incubated at 65°C for 5 min to elute RNA from the beads. This elution step was repeated once and the first and second eluates for a given sample were pooled. The RNA was subsequently purified by adding 2.75× volumes of 100% ethanol to the 400 μL solution recovered from aqueous phase of TRIzol in the presence of 20 μg of Ultrapure glycogen (Invitrogen 10814010). The pelleted RNA was subsequently washed per manufacturer’s instructions and resuspended in 5 μL of 10 μM App-VRA3_6N 3’ end adapter prepared using the 5’ DNA Adenylation Kit (New England Biolabs M2611A) and ligated using T4 RNA ligase 2, truncated KQ (New England Biolabs M0373) per manufacturer’s instructions with 15% (w/v) PEG-8000 in final solution and incubated overnight at 16°C. 180 μL of betaine binding buffer [1.42g of betaine (Sigma-Aldrich 61962) in 10 mL with binding buffer] was mixed with ligations and incubated for 5 min at 65°C and then for 2 min on ice. Capture on Streptavidin beads was again performed as above to enrich for ligated nascent RNAs except for the addition of a final wash with 1× MDE buffer (New England Biolabs B0608S) after the washes with high salt buffer, binding buffer, and low salt buffer. To prepare nascent RNA for 5’ end adapter ligation, the 5’ ends of the RNA were first decapped by mRNA decapping enzyme (New England Biolabs M0608S) for 1 h at 37°C. The beads were washed once in high, once with low salt buffer, and then once in 1× T4 PNK reaction buffer. The samples were then treated with T4 polynucleotide kinase (New England Biolabs M0201) for 1 h at 37°C for 5’-hydroxyl repairs, followed by one wash with high salt buffer, one wash with low salt buffer, and one wash with 0.25× T4 RNA ligase reaction buffer prior to resuspension in 5 μL of 10 pmol 5’ RNA adapter VRA5_6N. Next T4 RNA ligase 1 (New England Biolabs M0204L) was used to ligate the 5’ RNA adapter per manufacturer’s instructions with 15% (w/v) PEG-8000 in final solution and incubated overnight at 16°C. Following the 5’ end ligation, beads were washed twice with high salt buffer, twice with binding buffer, and twice with low salt buffer, then once with 0.25× SuperScript IV reaction buffer. Reverse transcription was performed using SuperScript IV Reverse Transcriptase (Invitrogen 18090010) and the cDNA was eluted from the beads by heating the samples twice for 30 seconds at 95°C. The eluted cDNA was initially amplified for 5-cycles of “preCR” [NEBNext Ultra II Q5 master mix (New England Biolabs M0544S) with Illumina TruSeq PCR primers RP-1 and RPI-X for indexing] following the manufacturer’s suggested PCR cycling protocol for library construction]. A portion of preCR was serially diluted and for test amplification to determine optimal amplification of final libraries. Libraries were amplified by 3 additional cycles of PCR (8 cycles in total). The amplified library was purified using the ProNex Size-Selective Purification System (Promega) and sequenced using the Illumina NovaSeq platform.

All the custom scripts used to process the PRO-seq data described herein are available on the AdelmanLab GitHub (https://github.com/AdelmanLab/NIH_scripts; https://zenodo.org/records/5519915). Dual, 6nt Unique Molecular Identifiers (UMIs) were extracted from read pairs using UMI-tools [10.1101/gr.209601.116]. Read pairs were trimmed using cutadapt 1.14. The UMI length was trimmed off the end of both reads to prevent read-through into the UMI of the other end of the sequence, which will happen for shorter fragments. An additional nucleotide was removed from the end of read 1 (R1), using seqtk trimfq (https://github.com/lh3/seqtk), to preserve a single mate orientation during alignment. The paired-end reads were then mapped to a combined genome index, including both the spike (dm6, drosophila) and primary (hg19, human) genomes, using bowtie2 [10.1038/nmeth.1923]. Properly paired-end reads were retained. These read pairs were then separated based on the genome (i.e. spike-in vs primary) to which they mapped, and both these spike and primary reads were independently deduplicated, again using UMI-tools. Reads mapping to the reference genome were separated according to whether they were R1 or R2, sorted via samtools 1.3.1 (-n), and subsequently converted to bedGraph format using a custom script (bowtie2stdBedGraph.pl; 10.5281/zenodo.5519915). We note that this script counts each read once at the exact 3’ end of the nascent RNA. Because R1 in PRO-seq reveals the position of the RNA 3’ end, the “+” and “-” strands were swapped to generate bedGraphs representing 3’ end positions at single nucleotide resolution. Samples displayed highly comparable recovery of spike-in reads, thus samples were normalized based on the DESeq2 size factors. Combined bedGraphs were generated by summing counts per nucleotide across replicates for each condition.

Annotated transcription start sites were obtained from human (GRCh37.87) GTFs from Ensembl. After removing transcripts with biotypes, PRO-seq signal in each sample was calculated in the window from the annotated transcription start site (TSS) to +150 nt downstream, using a custom script, make_heatmap.pl. This script counts each read one time, at the exact 3’ end location of the nascent RNA. Given good agreement between replicates and similar return of spike-in reads, bedGraphs were merged within conditions, and depth-normalized, to generate bigWig files binned at 10 bp. Browser snapshots shown in Figure 3 and S3 were generated with bigWig files and Integrative Genomics Viewer (https://software.broadinstitute.org/software/igv/).

#### Polysome-seq sample preparation

1×10^7^-2×10^7^ adherent cells were washed once with ice-cold PBS and subsequently lysed/scraped directly from the plates on ice using a polysome extraction buffer [(25 mM HEPES pH 7.5, 5 mM MgCl_2_, 100 mM KCl, 2 mM DTT, 1% Triton X-100, 0.1 mg/mL cycloheximide, 0.05 U/μL RNase inhibitor (Invitrogen AM2696), and 1× Roche-complete EDTA-free protease inhibitor cocktail (Roche 4693132001)]. The lysates were homogenized with 5–6 strokes in a glass Dounce homogenizer on ice, then cleared at 10,000×*g* for 5 min at 4°C. Cleared lysates were loaded on top of 10–50% linear sucrose gradients (mixed using a BioComp gradient mixer gradients containing 25 mM HEPES pH 7.5, 5 mM MgCl_2_, 100 mM KCl, 2 mM DTT, 0.1 mg/mL cycloheximide, and 1× Roche-complete EDTA-free protease inhibitor cocktail) and centrifuged in a Beckman Coulter ultracentrifuge at 40,000 rpm for 3 h at 4°C in a SW41Ti rotor. After centrifugation, gradients were fractionated and analyzed using a BioComp fractionator monitoring absorbance at 260 nm. Polysome profiles were manually inspected to determine fractions containing polysomes, and these fractions were pooled for RNA purification (polysome-containing fractions were identified as all fractions heavier than the monosome peak which was excluded). RNA was purified from pooled polysome sucrose gradient fractions using TRIzol LS (Invitrogen 10296028) followed by sequential isopropanol and ethanol precipitations. RNA quality was analyzed using an Agilent RNA TapeStation (RIN values all > 9), and Illumina sequencing libraries were prepared using a SMARTer Stranded Total RNA Sample Prep Kit - HI Mammalian (Takara 634873). Final sequencing libraries were quantified using Qubit HS DNA assays and an Agilent High Sensitivity TapeStation. Uniquely dual indexes libraries were pooled and sequenced on an Illumina NovaSeq 6000 at the Dana-Farber Cancer Institute Molecular Biology Core Facilities. The sequencing method was the same as described above for RNA-seq. 786-O samples (QJ8789) were sequenced with pair-end 100 bp reads, whereas A-498 samples (QJ9677) were sequenced with pair-end 150 bp reads.

#### Flow Cytometry to measure cell surface HLA complexes

786-O and A-498 cells expressing pVHL or EV were treated with either 100 ng/mL IFNγ (Peprotech 300–02) or vehicle for 72 h. The cells were then washed once with PBS, detached by Versene (Gibco 15040066), transferred to 15 mL conical tubes, and pelleted by centrifugation at 450×*g* for 5 min at RT. The cell pellets were next resuspended in cell culture media and passed through a cell strainer (Falcon 352235) to avoid cell clumps. The single cell suspension was centrifuged at 450×*g* for 5 min at RT, after which the cell pellets were resuspended at the concentration of 5×10^6^ cells/mL cell culture media. 200 μL of cells were used for each staining. 5 μL of Human TruStain FcX (Biolegend 422302) was added to each sample to block Fc receptors for 10 min at RT in the dark. 5 μL of Alexa 647 conjugated antibodies [W6/32 for HLA-Class I (Santa Cruz Biotechnology sc-32235 AF647) and its isotype control (Santa Cruz Biotechnology sc-24637)] were added to each sample, and the mixture was incubated for 30 min at RT in the dark. 4.5 mL PBS was added to each sample, after which the cells were pelleted by centrifugation at 450×*g* for 5 min at RT. The cell pellets were next resuspended in 500 μL of fixation buffer (Biolegend 420801) and analyzed at the Dana-Farber Cancer Institute Flow Cytometry Core Facility using BD LSRFortessa (BD Biosciences).

#### Immunopeptidomes – HLA-IP

To upregulate cell surface HLA class I protein expression, 786-O or A-498 cells were stimulated with 100 ng/mL IFNγ for 72 h and then detached with Versene, transferred to 50 mL conical tubes, and pelleted by centrifugation at 450×*g* for 5 min at 4°C. IP for HLA was performed using previously published methods^108,109^ with modifications. Each IP was conducted using 5×10^7^ cells or 0.1 g of tumor tissue in 6 replicates. Cell pellets or RCC tumor tissues were washed once with ice-cold PBS and lysed in 1 mL ice-cold lysis buffer [20 mM Tris pH 8.0, 1 mM EDTA pH 8.0, 100 mM NaCl, 1% Triton X-100, 60 mM octyl β-d-glucopyranoside (Sigma-Aldrich O8001), 6 mM Iodoacetamide (Sigma-Aldrich A3221), 20 U/mL DNase I (Roche 4536282001) supplemented with 1 mM phenylmethanesulfonyl fluoride (Sigma-Aldrich 78830) and 1× protease inhibitor. Cells and tissues were homogenized with Fisher 150 homogenizer using disposable plastic generator probes (Thermo Fisher Scientific 15–340-176). Cell lysates were centrifuged at 14,000×*g* for 22 min at 4°C. Cleared lysate supernatant was incubated with 10 μg of HLA-I antibody (Santa Cruz Biotechnology Clone W6/32, sc-32235) and coupled with 25 μL dry weight GammaBind Plus Sepharose beads (Cytiva 17088601) under rotary agitation for 3 h at 4°C. The beads were then thoroughly washed as follows to remove unbound protein and antibodies: once with wash buffer (20 mM Tris pH 8.0, 1 mM EDTA pH 8.0, 100 mM NaCl, 60 mM octyl β-d-glucopyranoside, 1% Triton X-100), three times with octyl buffer (20 mM Tris pH 8.0, 1 mM EDTA pH 8.0, 100 mM NaCl, 60 mM octyl β-d-glucopyranoside), twice with Tris buffer (20 mM Tris pH 8.0) and once with Millipore H_2_O. After the final wash any remaining supernatant was removed with a pipette tip hook to a vacuum. The beads were then allowed to air dry and stored at −80°C. Two IP samples for a given condition were combined in one MS run.

#### Immunopeptidomics - LC-MS/MS data generation

For both label free (786-O and A-498 cells) and TMT (786-O cells) immunopeptidome experiments, HLA-I bound peptides were eluted from the HLA-IP beads on top of Sep-Pak tC18 1 cc Vac Cartridges (Waters WAT054960) on a vacuum manifold. Cartridges were equilibrated twice with 200 μL MeOH, once with 100 μL 50% acetonitrile (ACN)/1% formic acid (FA), and then four times with 500 μL 1% FA. Beads with HLA-I bound peptides were resuspended in 200 μL 3% ACN/5% FA and transferred to the equilibrated cartridges. HLA-I bound peptides were eluted twice with 200 μL 10% acetic acid for 5 min and then desalted four times with 500 μL 1% FA. Finally, peptides were eluted from the desalting cartridge first with 250 μL 15% ACN/1% FA and then with 250 μL 50% ACN/1% FA. The two eluates were pooled and dried in a vacuum concentrator. For the TMT labeled samples, dried peptides from the acid elution and desalting described above, were reconstituted in 12.5 μL 50 mM HEPES (pH 7.0). Peptides from all samples were labelled with 5 μL 20 μg/μL TMT10 reagents (100 μg per channel) (Thermo Fisher Scientific 90406, Lot#SE240163) for 1 h at RT. Excess TMT reagent was quenched by addition of 1 μL 5% hydroxylamine. Six samples (EV +IFNγ, VHL+IFNγ in triplicates) as well as a common reference sample (used to normalize across multiple TMT plexes, data not included in this manuscript) were pooled (see Table S4 for channel layout) into a TMT-7plex. After pooling, samples were desalted on C18 StageTips and eluted into four fractions using increasing concentrations of ACN (3%, 10%, 15%, and 50%) in 5 mM ammonium formate (pH 10). Fractions were dried in a vacuum concentrator and stored at −80°C until LC–MS/MS data acquisition.

For the label free samples, HLA-peptides were secondarily desalted using bRP fractionation^110^, StageTips were prepared using two disks of SDB-XC material (Empore 2240), washed and equilibrated once with 100 μL 100% MeOH, once with 100 μL 50% ACN/1% FA, and then three times with 100 μL 1% FA. Dried peptides were resuspended in 200 μL 5% ACN/3% FA and loaded onto the StageTips. Peptides were desalted with 100 μL 1% FA three times before elution into three fractions with increasing concentrations of ACN (5%, 10%, and 30%) in 0.1% (w/v) NH_4_OH (Sigma–Aldrich 338818, pH 10). Fractions were dried in a vacuum concentrator and stored at −80°C until LC–MS/MS data acquisition.

For cell line samples, peptides were reconstituted in 3% ACN/5% FA before loading onto analytical columns [25–30 cm, 1.9 μm C18 (Dr. Maisch HPLC)] that were packed in-house into PicoFrit columns with 75 μm inner diameter, 10 μm emitters (New Objective). Peptides were eluted with a linear gradient (Thermo Fischer Scientific EasyNanoLC 1000) ranging from 6–30% buffer B (90% ACN/0.1 % FA) over 84 min, 30–90% buffer B over 9 min and held at 90% buffer B for 5 min at 200 nL min^−1^. MS/MS spectra were acquired on a Thermo Orbitrap Exploris 480 (Thermo Fisher Scientific) in data dependent acquisition.

For the TMT samples, the spray voltage was set at 1,800 V with the ion transfer tube temperature of 305°C and the MS1 spectra were collected at 60,000 resolution until either 100% normalized automatic gain control target or a maximum injection time of 50 ms was reached. Monoisotopic peak detection was set to “peptide” and “relax restrictions” was enabled; precursor fit filter was set to 50% fit threshold and 1.2 m/z fit window. Dynamic exclusion was 10 s. For HLA-I bound peptides, up to 10 precursors of charge 2+ to 4+ were subjected to MS/MS acquisition. Precursors were isolated with a 1.1 m/z window, collected with 50% normalized automatic gain control target and 100 ms maximum injection time, fragmented at 34% HCD collision energy, and acquired at 45,000 resolution.

For the label free samples, the spray voltage was set at 1,800 V with the ion transfer tube temperature of 305°C and the MS1 spectra were collected at 60,000 resolution until either 100% normalized automatic gain control target or a maximum injection time of 50 ms was reached. Monoisotopic peak detection was set to “peptide,” and “relax restrictions” was enabled; precursor fit filter was set to 50% fit threshold and 1.2 m/z fit window. Dynamic exclusion was 10 s. For HLA-I bound peptides, up to five precursors of charge 1+ between 800 and 1700 m/z or 10 precursors of charge 2+ to 4+ were subjected to MS/MS acquisition. Precursors were isolated with a 1.1 m/z window, collected with 50% normalized automatic gain control target and 100 ms maximum injection time, fragmented at 30% HCD collision energy, and acquired at 15,000 resolution.

For RCC patient samples, data were generated over a 3-year period, 2019–2021. The data for samples 101, 102, 104, and 105 was previously published^110^, and used similar methods to the label-free cell line samples described above. Replicate data were collected both with and without SDB-XC fractionation and with and without FAIMS (CVs −50 and −70) for most samples. Data for samples 101, 102, and 106 was generated with a Thermo Orbitrap Fusion Lumos instrument using the Xcalibur version 3.1.2412.25. Data for all other samples was generated with the same Thermo Orbitrap Exploris 480 used for the cell line samples using the Xcalibur versions 1.1–1.1.75.22/1.1.117.22, 2.0–2.0.122.16/2.0.182.25, 3.0–3.0.204.12/3.0.261.13, 3.1–3.1.231.6/3.1.279.9.

#### Immunopeptidomic – LC-MS/MS data analysis

Spectrum Mill (SM) v8.02 (proteomics.broadinstitute.org) was used to prepare sequence databases and identify peptides in LC-MS/MS datasets.

##### Sequence database construction

To search LC-MS/MS spectra to find ERV-derived peptides, we prepared a sequence database containing both ERV proteome and reference proteome. The ERV proteome was constructed from the 3173 human ERVs described in Vargiu *et al*. with supplemental Table 1^41^ converted to a .bed file and GRCh37 (hg19) nucleotide sequences extracted with QUILTS^111^ v3 (https://github.com/ekawaler/pyQUILTS). 6-frame translation was performed using the SM sequence database utilities with minimum protein length 7 and Met start for each ORF not required. We started with nucleotide ERV sequences from the Vargiu database^41^ rather than protein sequences from the gEVE database^112^, because gEVE imposes an ORF length minimum of 80 amino acids (AAs). In practice, the well-studied Childs’ peptide ATFLGSLTWK may be derived from 7 possible relatively short ERV ORFs (length 21–61 AAs). Several peptides identified in this study may also be derived from ORFs shorter than 80 AAs. In addition, the Vargiu database has been used in prior studies, including Smith *et al*^33^ and Kobayashi *et al*^81^, where immunogenic peptides derived from ERVs for ccRCCs were discovered. Subsets of the HIF2-responsive ERVs for the RCC lines 786-O (57 ERVs), A-498 (31 ERVs), or 786-O + A-498 (81 ERVs - 44,747 proteins, 721,483 peptide 9-mers) were 6-frame translated as above and appended to the base reference proteome for searching the LC-MS/MS data from 786-O cells, A-498 cells, or RCC patient tissue, respectively.

For the reference proteome, equivalent versions of the last GRCh37 (hg19) genome assembly were used as the reference genome (Broad GRCh37/b37) for RNA-seq analyses, and the reference proteome (Ensembl v38), ftp.ensembl.org/pub/grch37/current/fasta/homo_sapiens/pep/Homo_sapiens.GRCh37.p ep.all.fa.gz. The Ensembl v38 protein fasta file was filtered to retain non-redundant entries from standard chromosomes (not GL, H, LRG) with transcript_biotype – protein coding, and minimum length 7. The base reference proteome consisted of Ensembl v38 (71,704 proteins), along with common laboratory contaminants (602 proteins). For RCC patient tissues, the 81 ERVs described above appended to the base reference proteome yields relative unique 9-mer contributions of 94% and 6% for Ensembl v38 + contaminants, and ERVs, respectively, while the ERV contributions were 4% and 2% for the 786-O and A-498 cell lines, respectively.

##### Spectrum quality filtering

Using the SM Data Extractor module similar MS/MS spectra in the same chromatographic peak with the same precursor mass were merged. The precursor MH + inclusion range was 800–6000 (TMT labeled samples), 600–4000 (RCC cell lines), or 700–2000 (RCC patient). The search-time spectral quality filter was a sequence tag length >1 (i.e., minimum of three peaks separated by the in-chain masses of two consecutive AAs).

##### MS/MS search conditions

Parameters for the SM MS/MS search module for HLA-I immunopeptidomes included: no enzyme specificity; precursor and product mass tolerance of ±10 ppm; minimum matched peak intensity of 40%; ESI-QEXACTIVE-HCD-HLA-v3 scoring; fixed modification: carbamidomethylation of cysteine; variable modifications: cysteinylation of cysteine, oxidation of methionine, deamidation of asparagine, acetylation of protein N-termini, and pyroglutamic acid at peptide N-terminal glutamine; and precursor mass shift range of −18 to 81 Da for unlabeled sample. For TMT-labeled samples the fixed modifications also included TMT10 Full-Lys.

##### Peptide-spectrum match filtering and FDRs

Using the SM Autovalidation module, peptide-spectrum matches (PSMs) were confidently assigned by applying target-decoy based FDR estimation to achieve <1.0% FDR at the PSM level in aggregate for peptides from all sources. PSM-level thresholding was done with a minimum backbone cleavage score of 5, allowed precursor charges were 1–4, allowed peptide length was 8–11 AAs, and <1.0% FDR for each LC-MS/MS run. Immunopeptidomics data were further filtered to remove non-human contaminants.

##### Machine learning FDR filtering

While the aggregate FDR for each dataset was set to <1%, as described above, FDR for certain subsets of rarely observed classes (<5% of total) of peptides required more stringent score thresholding to reach a suitable subset-specific FDR < 1.0%. To this end, we devised and applied machine learning based filtering approaches using the SM Multi Metric FDR estimation module with machine learning based metrics of spectrum similarity of a measured MS/MS spectrum to the predicted b/y intensities from Prosit^113^, deviation of the measured liquid chromatography retention times (RT) to the predicted RTs from DeepLC^114^, and HLAthena binding percentile ranks^115^. A spectral angle, dot product measure of spectrum similarity (SA_DP) was calculated for each PSM between the intensities of b/y ions in a measured MS/MS spectrum and the b/y intensities predicted by Prosit (with model Prosit 2020 intensity HCD or Prosit 2020 intensity TMT for unlabeled and labeled datasets, respectively, koina.proteomicsdb.org) using the collision energy and precursor charge of the measured MS/MS spectrum. Deviation of measured RT from predicted RT was calculated for each peptide using RTs predicted by DeepLC v2.2.36 for all identified peptides after the DeepLC calibration routine was provided with a subset of peptides obtained by dividing the entire RT range into 50 segments and selecting the top 15 human reference proteome derived peptides ranked by identification score. HLAthena binding prediction percentile ranks for the best scoring allele for each peptide were classified into 3 categories (<0.5: strong binders; 0.5 – 2: weak binders; >2 non binders).

Peptides that mapped to both canonical reference proteome and ERVs were eliminated, including elimination of peptides with sequence differing only by Leucine/Isoleucine substitutions because those two AAs are indistinguishable by mass. The remaining list of ERV-derived peptides matched in any patient sample was further thresholded. ERV subset-specific PSM scoring metric thresholds were tightened in a fixed manner for all ERV PSMs so that distributions for each metric improved to meet or exceed the aggregate distributions. The fixed thresholds were: minimum score: 7, minimum percent scored peak intensity: 50%, minimum backbone cleavage score (BCS): 5. Since each machine-learning companion program prediction has strengths and weaknesses, score thresholds were applied for each metric to roughly classify a PSM into a pass/fail status: Prosit b/y intensity (pass: SA_DP > 0.6), DeepLC RT (pass: deltaRT within ± 10 min), and HLAthena binding category (pass: strong/weak percentile rank < 2.0). Each ERV-derived PSM was then classified depending on the number of strikes against: 0 - pass, 1 - maybe, 2 or 3 – fail (Figure 4B). In Table S4 and Table S6 the classifications are shown as green, yellow and red, respectively.

##### BLASTP to find alternative protein sources

We retained, but annotated in Tables S4 and S6, peptides that mapped to both non-coding source and ERVs. We also listed all possible ERVs for peptides that could be produced by multiple ERVs.

##### Statistical analyses for TMT immunopeptidomics

Proteomics Toolset for Integrative Data Analysis (Protigy, v1.1.4, Broad Institute, https://github.com/broadinstitute/protigy) was used to calculate moderated t-test P values.

#### Peptide synthesis

Peptides were synthesized at purity of >95% by RS synthesis (https://rssynthesis.com). Lyophilized powder was resuspended in DMSO at the concentration of 20 mg/mL. The two synthetic peptides used for spectrum validation in Figures 4E, 4F, and S5E, each contained a heavy lysine [KLIAGLIFL(K) and ATFLGSLTG(K), K=lys (^13^C6, ^15^N2), 8 Da shift in mass].

#### HLA-I antigen presentation prediction

HLA class I presentation for all unique ERV derived peptides (8–11 mer) across various HLAs of 786-O cells, A-498 cells, and 11 ccRCC patients from the NCT02950766 clinical trial, was predicted using HLAthena (an epitope prediction tool trained on endogenous LC-MS/MS-identified epitope data using the website: http://hlathena.tools) with the MSi (mass spectrometry intrinsic) model and allele typing for each cell line and ccRCC patient^115^. HLAthena percentile ranks for the best scoring allele are reported (<0.5 strong binders; 0.5–2: weak binders; >2 non-binders). For the RCC patients that underwent allo-SCT, HLA class I binding predictions for ERV peptides were performed across all identified donor alleles using both the HLAthena MSi prediction model as well as NetMHC. Any peptide classified as strong binders, either by the HLAthena MSi prediction model (HLAthena percentile rank<0.5) or the NetMHC prediction model (NetMHC percentile rank<0.5), was selected for immunogenicity screening in the corresponding donor.

#### Single-cell data processing and analysis

##### Map Vargiu database to the hg38 genome assembly

To map 3173 ERVs from the database described in Vagiu *et al* ^41^ (based on hg19 genome assembly) to the hg38 genome assembly, we used the UCSC LiftOver tool (https://genome.ucsc.edu/cgi-bin/hgLiftOver). In the conversion process, after downloading the appropriate chain file, the hg19 ERV coordinates were converted to hg38 coordinates in BED file format. Out of the 3173 ERVs, 3164 were successfully mapped, leaving only 9 ERVs unmapped. We obtained the FASTA file for the hg38 ERVs using BEDTools.

##### Single-cell ATAC-seq data processing and analysis

Single-cell ATAC-seq (scATAC-seq) data published by Yu *et al*^94^ were downloaded as 10× Cell-Ranger ATAC outputs (fragments.tsv.gz, peaks.bed, and singlecell.csv files) that were pre-aligned to GRCh38/hg38 and submitted to GEO (accession number: GSE207493). Samples that were reported to be *VHL* wild-type (RCC87, RCC100, RCC116) were excluded from all downstream analyses. Sample fragment files were first indexed using Tabix (v1.2.1)^116^, before being imported into R (v4.4.1), and RStudio (v2023.09.1+494). Signac (v1.13.0)^117^ and Seurat (v5.1.0)^118^ were used to import sample files into chromatin assay objects, followed by Seurat objects. Individual peak files were combined to create a consensus peak set. Individual Seurat objects were then merged and annotated using hg38 before using scDblFinder (v1.18.0), using ‘aggregatefeatures=TRUE’^119^ to filter out cells inferred to be potential doublets. We further filtered the data by computing and setting thresholds for the following parameters: FRiP > 0.20, TSS Enrichment > 1.4, Nucleosome Signal < 2.5, Blacklist Fraction < 0.1, and Peak Region Fragments > 3000; < 10000. We reduced the dimensionality of the scATAC-seq data, as described in the original Signac pipeline, by performing latent semantic indexing (LSI) which entails performing term frequency-inverse document frequency normalization followed by singular value decomposition on the merged Seurat object^117^. We batch corrected the resulting LSI components using Harmony (v1.2.0)^120^, using sample ID as variable. *UMAP* and *FindNeighbors* were performed using the 2^nd^ through 30^th^ Harmony-adjusted LSI embeddings. *FindClusters* was performed using a resolution of 0.05. Cell type or cluster annotation was performed by measuring chromatin accessibility at promoters for cell type-specific marker genes such as *CA9* for ‘Cancer’ cells^121^, *PTPRC* and *GZMB*, for ‘Immune’ cells^122^, *VWF* and *PECAM1* for endothelial cells^123^, and *COL4A1* and *PDGFRA* for interstitial cells^124^. To validate our assignment of cells to the ‘Cancer’ identity, we retrospectively used CopyscAT^125^, which uses scATAC-seq fragment data to infer copy number variation at the chromosome arm level. Tumors 84, 86, and 96 were filtered out as we could not conclusively identify cancer cells using CopyscAT. We used this tool on a sample-by-sample basis, by setting ‘Immune’ and ‘Other’ cells as ‘Normal’ cells. We then combined the samples and looked for inferred chromosome 3p copy number, setting a cutoff of 0–2. We then proceeded to compute differential accessibility using the *FindMarkers* function of the Seurat package, using logistic regression, and setting ‘min.pct’ to 0.01 and ‘logfc.threshold’ to ‘0’. We termed significant peaks to be those that had an adjusted p-value < 0.05 and |Log2FC| > 1. Peaks that were computed to have an adjusted p-value of 0 were assigned an arbitrary log adjusted p-value of 300 for visualization purposes. To determine differential accessibility at ERVs, we first used Bedtools (v2.31.0)^107^ intersect to compute accessible chromatin peaks filtered by genomic coordinates for ERVs ± 3kb. We then used the *findOverlaps* function of the GenomicRanges R package (v3.19)^126^ to annotate these differentially accessible peaks at ERVs +/− 3 kb by their respective ERVs. We used ChIPseeker (v1.40.0)^127^ to annotate unfiltered differentially accessible peaks at coding features of the genome, specifically to compare differential accessibility at ERVs to known cell type-specific markers such as *CA9* and *PTPRC*. HIF2-responsive ERVs with multiple peaks scoring as ‘Up’ and ‘Unchanged’, or ‘Down’ and ‘Unchanged’ were scored as ‘Up’ and ‘Down’ respectively. One HIF2-responsive ERV (ERV6084) was computed to have both ‘Up’ and ‘Down’ peaks. This ERV was excluded from downstream analyses. Finally, using the *CoveragePlot* function of Signac, we generated chromatin accessibility genome browser tracks at specific genomic loci.

##### Single-cell RNA-seq data processing and analysis

Single-cell RNA-seq (scRNA-seq) data were also obtained^94^ as ‘possorted.bam’ file outputs from 10× Cell Ranger count (GSE207493), that were aligned to GRCh38/hg38. As with the scATAC-seq data, samples that were reported to be *VHL* wild-type (RCC87, RCC100, RCC116) were excluded from all downstream analyses. Bam files were filtered for reads containing ‘UB’ and ‘CB’ tags using Samtools ‘view’^128^. The tool ‘scTE’^129^ was used to map single cell RNA-seq reads to genes and ERVs. This was done by first generating a custom index using a bed file containing hg38 coordinates of ERV loci, and a Gencode GRCh38.p14 (release 46) ‘basic gene annotation’ GTF file using ‘scTE_build’ and specifying ‘UB’ and ‘CB’ tags. Resulting ‘.h5ad’ files were imported into R using the *ReadH5AD* function of the SeuratDisk R package ‘https://mojaveazure.github.io/Seuratdisk’. Individual Seurat objects for each sample were then merged, after which the data were normalized and scaled, and then principal component analysis (PCA) was used as the method of dimensionality reduction. As described above, potential doublets identified using scDblFinder with default settings were removed. Next, cells containing greater than 10% mitochondrial reads, fewer than 200 features, or more than 3000 features were filtered out. As described above, batch effect correction was performed on PCs using Harmony, after which *UMAP* and *FindNeighbors* were performed using the first through the tenth Harmony-adjusted PCs. Next, *FindClusters* was performed using a resolution of ‘0.5’. Cell type or cluster annotation was then performed by measuring the expression of cell type-specific markers, like the annotation of scATAC-seq clusters, with genes such as *CA9*, *NDUFA4L2* strongly expressed by cells that were termed ‘Cancer’, *PTPRC*, *GZMB* expressing cells termed ‘Immune’, and *PECAM1*, *VWF*, *COL4A1*, *PDGFRA* expressing cells termed ‘Other’. Differentially expressed ERVs were computed using the FindMarkers function of Seurat, using the Wilcoxon sum rank test, and ‘min.pct’ set to 0.01 and ‘logfc.threshold’ set to ‘0’. We termed significant ERVs to be those that had an adjusted p-value < 0.05 and |Log2FC| > 1. ERVs that were computed to have an adjusted p-value of 0 were assigned an arbitrary log adjusted p-value of 305 for visualization purposes.

#### Human patient samples

The source of the biospecimens (tumor, normal and blood samples) used in this study were pre-vaccination samples from patients enrolled in a clinical trial (NCT02950766). Grossly viable tumor and adjacent normal tissue was placed in DMEM complete media [DMEM (Gibco 11995065) supplemented with 10% FBS (Gibco A5256701) and 1% penicillin-streptomycin] on ice then immediately used for downstream processing and analysis.

When available, additional RNA-seq from purified cancer cells (i.e. absent other cells from the microenvironment) was performed. Fresh tumors (isolated from RCC patients from the NCT02950766 clinical trial) were first minced by scalpels into 1–2 mm^3^ pieces and then enzymatically dissociated into a single cell suspension by incubation in digestion buffer [HBSS buffer (Life Technologies 14175095) supplemented with 0.11 U/mL collagenase D (Roche 1108858001), 0.56 U/mL dispase (STEMCELL Technologies 07913), 50 U/mL DNase I (New England Biolabs M0303L), 5 mM calcium chloride (VWR E506–100ML)]. Carbonic anhydrase IX (CA9)-positive cancer cells were immunomagnetically purified using anti-CA9 PE antibody (Miltenyi Biotec 130–123-299) from freshly dissociated tumors according to manufacturer protocol and rested for 48–72 h in RCC media [OptiMEM GlutaMax media (Gibco 5198091) supplemented with 5% FBS, 1 mM sodium pyruvate, 1% penicillin-streptomycin, 50 μg/mL gentamicin (Sigma-Aldrich G1397–10ML), 5 μg/mL insulin (Sigma-Aldrich I9278–5ML), and 5 ng/mL EGF (Miltenyi Biotec 130–093-825). Floating cells were washed off and only attached cancer cells were harvested. RNA was extracted using the RNeasy Micro Kit (Qiagen 74004), and RNAseq of this low-input RNA was performed using a modified version of the Smart-seq2 protocol (using NEBNext Single Cell/Low Input cDNA Synthesis & Amplification module), as previously described^130^. For one tumor sample (from patient 110), the initial low-input RNA-seq protocol was unsuccessful. However, a primary cell line was successfully generated and kept in culture and was used for RNA-seq using the same protocols. The raw RNA-seq data were deposited as part of the NeoVax (NCT02950766) clinical trial data in dbGaP (accession number: phs003710.v1).

PBMCs were isolated by Ficoll density gradient centrifugation (GE Healthcare) and cryopreserved. For the ELISpot experiments in Figure 6, PBMCs were cultured in HS media [DMEM (Gibco 11965092) supplemented with 10% human serum (Sigma-Aldrich H4522), 10 mM HEPES (Gibco 15630080), 1× non-essential amino acids, 1 mM sodium pyruvate, 4% penicillin-streptomycin, 55 μM 2-mercaptoethnol (Gibco 21985023); media was filtered using the CELLTREAT Filter System (CELLTREAT Scientific Products 229707) prior to use]. Cryopreserved PBMCs were thawed at 37°C and cultured in HS media with 10 ng/mL IL-7 (Peprotech 200–07) for 16–24 h. Then the PBMCs were pelleted at 450×*g* for 5 min at RT and the cell pellets were resuspended in 7212 media [DMEM supplemented with 20% FBS, 10 mM HEPES, 1× non-essential amino acids, 1 mM sodium pyruvate, 4% penicillin-streptomycin; media was filtered using the CELLTREAT Filter System prior to use] in preparation for the ELISpot assays.

For the ELISpot experiments in Figure 7, cryopreserved PBMCs were thawed and plated in AIM-V media (Gibco 12055091) supplemented with 20 IU/mL IL-7, 1% GlutaMAX (Gibco 35050061), 10% CTS immune cell serum replacement (Gibco A2596101), 10 mM HEPES, 1× non-essential amino acids, 1 mM sodium pyruvate, 1% penicillin-streptomycin, and 55 μM 2-mercaptoethanol. Media was filtered using the CELLTREAT Filter System. PBMCs were rested overnight and then peptide-pulsed at 10 μg/mL. Three days post-stimulation, the media was changed to AIM-V media supplemented with 100 IU/mL IL-2 (Peprotech 200–02) and 20 IU/mL IL-7. 12 days post-stimulation, PBMCs were switched to cytokine-free AIM-V media and rested for 48 h, after which immune response was measured by IFNɣ ELISpot.

#### Mouse vaccination experiment

ERV peptide pools were prepared using 50 μg of each peptide (11 peptides, synthesized by RS Synthesis, peptide sequence listed in Table S6) per immunization. The peptide pool was supplemented with 15 μg Hiltonol (Poly ICLC Oncovir) and 200 μg freeze-dried Mycobacterium Tuberculosis H37Ra (BD Biosciences Difco DF3114–33-8) mix per immunization. The peptides were emulsified with complete Freund’s adjuvant (BD Biosciences Difco DF0638–60-7). Transgenic mice that express HLA-A*11:01 allele [Taconic CB6F1-Tg(HLA-A*1101/H2-Kb)A11.01] were immunized with 200 μL of the emulsion in the groin/flank. Three weeks post immunization, the mice were boosted with the same vaccine emulsion on the opposite site. Mice were euthanized on Day10 post vaccination using CO_2_. Their spleens were then surgically removed, placed onto a 70 μm strainer on cell culture plates, gently pressed through the strainer using the back of a 3 mL syringe plunger (BD Biosciences BECD-309657-A). Cells that passed through the 70 μm strainer were collected and transferred into 50 mL conical tubes and pelleted by centrifugation at 450×*g* for 5 min at RT. Red blood cells were osmotically lysed by resuspending the cell pellets in 9 mL of deionized H_2_O, followed immediately by 1 mL of 10× PBS and an additional 20 mL of cell culture media to avoid lysing the other cells. The conical tubes were inverted a few times to ensure thorough mixing, after which the cells were pelleted by centrifugation at 450×*g* for 5 min at RT. The supernatant was removed and splenocytes were resuspended in 7212 media in preparation for the ELISpot assays.

#### ELISpot assay

ELISpot was performed using red blood cell-depleted mouse splenocytes or human PBMCs according to manufacturer’s protocol (Mabtech). Briefly, opaque ELISpot plates (Millipore MSIPS4W10) were wetted with 35% ethanol briefly and then washed 3–4 times with deionized H_2_O. The plates were then coated with 100 μL anti-IFNγ capture antibodies [anti-mouse IFNγ antibody (Mabtech 3321–3-250) at the final concentration of 8 μg/mL, or anti-human IFNγ antibody (Mabtech 3420–3-250) at the final concentration of 2 μg/mL diluted in PBS (Corning MT21040CV)] overnight at 4°C. The plates were then washed 6 times with PBS and blocked with 200 μL 7212 media (Figure 6) or 50 μL AIM-V media (Figure 7) while preparing the cell/peptide mixture. For the assays in Figure 6, splenocytes or PBMCs were plated at 200,000 cells/well, 250 μL total volume for each well. For the assays in Figure 7, a variable number of cells were plated in 250 μL total volume for each well. Cells were co-incubated with the individual peptides (at the final concentration of 10 μg/mL) in triplicates in ELISpot plates for 18 h-24 h. Plates were then washed 4 times with 0.5% FBS/PBS (splenocytes) or PBS (PBMCs). 100 μL secondary antibodies [anti-mouse secondary antibody (Mabtech 3321–6-250) at the final concentration of 2 μg/mL, or anti-human secondary antibody (Mabtech 3420–6-250) at the final concentration of 1 μg/mL diluted in PBS] were added to each well and incubated in the dark for 2 h (splenocytes) or 1 h (PBMCs) at RT. The wells were then washed 6 times with PBS and incubated with 100 μL streptavidin-HRP (Mabtech 3310–9-1000, diluted 1:1375 in PBS) in the dark for 1 h at RT. The wells were then washed 4 times with PBS before adding 100 μL TMB substrate (Mabtech 3651–10) per well. The wells were incubated for 2–6 min until the spots in wells containing positive controls were completely developed. The development was stopped by washing the plates with deionized H_2_O six times. Plates were air-dried and then imaged using an ImmunoSpot Series Analyzer (Cellular Technology, Ltd, Cleveland, OH)^109,131^. Images in Figure S6 were converted to grayscale for easier visualization. Any brightness and contrast adjustment were applied uniformly across images for each patient. For wells with unquantifiable spot number due to high count number (e.g. in the case of PHA), an arbitrary number was assigned, which equaled to the highest countable number of other wells on the same plate. HIV peptide (SLYNTVATL)^132^ treated wells served as negative controls. For splenocyte experiments, anti-CD3 antibody (BD Biosciences 553058, at the final concentration of 0.4 μg/mL) served as a positive control. For human PBMC experiments, the CEF peptides (Mabtech 3618–1 at the final concentration of 10 μg/mL) or PHA (Life Technologies 10576–015 at the final concentration of 0.2% v/v) served as positive controls.

#### T-Scan screening and analysis

##### Whole Exome Sequencing (WES)

DNA was extracted from tumor tissue and normal PBMCs for the 7 patients (ccRCC1-ccRCC7) and prepared for WES. Samples from ccRCC3 were not submitted for WES due to insufficient material. Sequencing libraries were prepared and sequenced at Fornax Biotech (Worcester, MA). Libraries were prepared using SureSelect XT HS reagents and kits and Human All Exon V7 Target Enrichment Baits according to the manufacturer specifications (Agilent). Pooled libraries were sequenced on NextSeq 500/550 Mid-output v2.5 kit (300 cycles). Each sample was sequenced at 150× - 400× depth. Variant calling was performed using both Strelka (Illumina) and MuTect (The Broad Institute) by comparing each patient’s tumor DNA to a normal reference (same patient’s PBMC DNA).

##### Isolation of CD8+ TILs from dissociated tumors

Dissociated tumor cells were thawed in RPMI medium supplemented with 10% FBS. Cell pellets were stained with Zombie aqua dye and subsequently labelled with TotalSeq^™^-C Human Universal Cocktail, followed by staining with fluorescent antibodies (CD8 PE-Cy7, CD45 AF488, CD3 APC-Cy7, CD4 PE, and EpCam APC) according to manufacturer protocol (BioLegend). Labelled cells were sorted to collect live, CD45+, CD3+, CD8+ cells using a MoFlo Astrios EQ cell sorter (Beckman Coulter) and submitted for single cell TCR sequencing.

##### Single Cell TCR sequencing and analysis

Single-cell TCR-seq (scTCR-seq) libraries were prepared following the 10x Genomics Single Cell 5’ V2 Immune Sequencing Kit protocol. Briefly, single cells were captured in droplets before undergoing reverse transcription. Following cDNA purification, cDNA was amplified and purified. 2 μL of each library was then used for TCR sequence enrichment. TCR enriched libraries were fragmented, end-repaired, and amplified with indexing primers. The scTCR-seq libraries were sequenced on an Illumina NextSeq550 using a High Output v2.5 kit (150 cycles) with read lengths: 26 bp- read 1, 10 bp- i7 index, 10 bp- i5 index, 90 bp- read 2.

scTCR-seq reads were processed using Cellranger VDJ 5.0.1 pipeline with the reference vdj-GRCh38-alts-ensembl-5.0.0. We applied custom Python software to filter for productive paired TCR clonotypes. TCR clonotypes were quantified for each sample. Expanded TCR clonotypes were synthesized (GenScript) and cloned into a Lentiviral expression vector for subsequent T cell engineering and genome wide screens as previously described^133^. Excepting ccRCC7, ‘expanded’ TCR clonotypes were defined as comprising ≥ 1% of total TCR frequency of the CD8+ TILs. For ccRCC7, clonotypes present at ≥ 0.5% frequency post-treatment were included. Additionally, clonotypes that expanded ≥ 2-fold post-treatment compared to pre-treatment frequencies were included for screening.

##### CD8 T cell engineering

CD8+ T cells were isolated, activated, and transduced with lentiviral particles to express orphan RCC TCRs as previously described^133^.

##### Reporter cell line engineering

MHC null HEK293T cells were engineered to express a fluorescent reporter activated by T cell killing as described in^62,93^. HLA alleles relevant to each patient were introduced into reporter cells, which were then transduced with a genome-wide library of protein fragments, as described^62^.

##### T-Scan Screen

Genome-wide screens were performed and analyzed as previously described^62,93,133^.

##### Epitope Mapping Assay

Specific MHC-binding epitopes for each scoring tile were predicted using MHCflurry. Candidate epitopes for each target were selected by identifying predicted strong binding epitopes shared across overlapping adjacent and redundant tiles that were enriched in the screen.

HEK293T reporter cells expressing the relevant HLA alleles were seeded in a 96 well plate and pulsed with the target peptide (Genscript) at 1 μg/mL for 1 h. For target 1514, reporter cells were stably transduced with the 63AA protein fragment clone that scored in the genome wide screen and seeded. Transduced or peptide-pulsed cells were then co-cultured with the TCR-transduced T cells at an E:T ratio of 1:1 for 4 h. Cells were harvested by pipetting into single-cell suspensions and evaluated for IFP fluorescence by flow cytometry (Cytoflex S, Beckman Coulter).

### Quantification and statistical analysis

Significance of all comparisons, excepted what are listed below, was calculated by unpaired two-tailed t test with cutoffs as follows: *p<.05, **p<.01, ***p<.001, ****p<.0001. For Figure 2E, Expression data were log-transformed and z-score normalized. Signatures were calculated by averaging the normalized expression data of each gene set. P-value were adjusted for multiple comparisons using the Benjamini-Hochberg method for each gene/pathway. *: *p.adj*<0.05; For Figure 4C, moderated t-test P values were calculated based on the log2 transformed, median normalized TMT intensities of the identified HLA peptides. P values were adjusted for multiple hypothesis testing using the Benjamini-Hochberg method; For Figures 5F and 5G, significance was determined by Kruskal-Wallis test followed by Dunn’s multiple comparisons test. ns = not significant, **p<.01, ***p<.001, ****p<.0001. For all comparisons, except what are listed below, data were presented as means ± SD. For Figures 3E, 7A–7C, S2C, S6E and S6F, data were presented as means ± SEM. N represents number of biological replicates (except Figure 7F, N represents technical replicates), or number of mice.

## Supplementary Material

1

2 **Figure S2.** Identification of HIF2 Binding Sites Near ERVs, Related to Figure 3 (A) Anti-HIF2α ChIP-seq tracks (blue) in 786-O cells that underwent CRISPR-based gene editing with a HIF2α sgRNA or a control sgRNA (sgCtrl) and Anti-FLAG ChIP-seq tracks (purple) in 786-O, OS-RC-2 and RCC4 cells in which a FLAG-HA epitope tag coding sequence was inserted at the 5’ end of the endogenous *HIF2*α open reading frame by CRISPR-HDR (FLAG-HIF2α). Parental cells infected with a control guide RNA (sgAAVS1) were included as controls. All the experiments were conducted in duplicates except for Anti-HIF2α ChIP-seq in 786-O cells with a HIF2α sgRNA, which was conducted only once. Rep 1 corresponds to the data in Figure 3D. (B) Anti-FLAG immunoblot analysis for 4 different clones of 786-O cells in which a FLAG-HA epitope tag coding sequence was inserted at the 5’ end of the endogenous *HIF2*α open reading frame by CRISPR-HDR compared to parental cells that were infected with a control guide RNA (sgAAVS1). 786-O cells stably infected to express pVHL (VHL) or with the empty vector (EV) were included as additional controls. (C) Anti-FLAG ChIP-qPCR assays using two different clones (CL15 and CL53) of 786-O cells as shown in (B). Cells were treated with 2 μM PT2399 or vehicle for 72 hours prior to ChIP-qPCR with primers designed to interrogate HREs for the indicated ERVs. Data were normalized to the untreated sgAAVS1 cells. (N=4) (D and E) Immunoblot analysis (D) and Venn Diagram of DNMT1i-responsive ERVs based on RNA-seq (E) of 786-O cells that underwent CRISPR-based gene editing with a HIF2α sgRNA or control sgRNA (sgCtrl) and then treated with 0.5 μM GSK3685032 (DNMT1i) or vehicle (Veh) for 9 days. Note that in (E) 28 ERVs were derepressed by treatment with 0.5 μM GSK3685032 (DNMT1i) for 9 days compared to vehicle-treated cells in a HIF2-dependent manner. (F and G) RT-qPCR validation (F) and anti-HIF2α ChIP-seq (G) of 3 ERVs that were 1) one of the 28 as shown in (E) and 2) located within 10 kb of a HIF2α binding site that was induced by GSK3685032 DNMT1i treatment based on anti-HIF2α ChIP-seq. The cells underwent CRISPR-based gene editing with a HIF2α sgRNA or a control sgRNA (sgCtrl) and were then treated with 0.5 μM GSK3685032 (DNMT1i) or vehicle (Veh) for 9 days. Rep1 corresponds to the data in Figure 1M. (N=3)

3 **Figure S3.** ERVs that are Direct HIF2 Transcriptional Targets in OS-RC-2 Cells, Related to Figure 3 (A) ERV levels in OS-RC-2 cells treated with either 2 μM PT2399 or vehicle for 72 hours. ERV RNA levels were normalized to *ACTB* RNA levels and then to the vehicle condition. (N=3) (B) Representative nascent RNA tracks in OS-RC-2 cells that were treated with 2 μM PT2399 (PT) or vehicle (Veh) for 2 hours. All 8 listed ERVs scored as HIF2-responsive in the PRO-seq analysis. The housekeeping genes *ACTB, VCL* and *TUBA1A* were included as controls.

4

5 **Figure S5.** Human HIF2-responsive ERVs are Largely Derived from Cancer Cells, related to Figure 5; MS/MS spectra for ERV-derived ATFLGSLTGK peptide, related to Figure 6 (A) Coverage plot showing scATAC-seq tracks at the housekeeping genes *ACTB*, *VCL* and *TUBA1A* ± 3 kb. (B) UMAP for scATAC-seq data from Yu *et al*^60^. In left panel, cell type inferred based on chromatin accessibility at promoters of cell type specific genes. In the middle and right panels, chromosome 3p and 3q copy number inferred using CopyscAT. (C) Violin plots depicting RNA levels for the indicated ERVs and genes in Cancer or Immune cells based on scRNA-seq data from Yu *et al*^60^. (D) Heatmap showing the abundance of the 81 HIF2-responsive ERVs based on Log_**10**_(TPM, transcripts per million)+1 of the RNA-seq data from CA9-enriched tumor samples derived from patients enrolled in the NCT02950766 clinical trial. Samples are labeled as “1–8”, the detailed information for each tumor sample is listed in Table S5. (E) MS/MS spectra comparing HLA-bound ERV-derived peptide ATFLGSLTGK from tumor immunopeptidome of RCC patient 110 (1 out of 3 patient with alleles HLA-A*03:01 or -A*11:01) (upper) and the corresponding synthetic peptide bearing a heavy lysine (Lys8, lower). The tumor spectrum has a Spectrum Mill sequence coverage metric, backbone cleavage score (BCS) of 4 that is below the automated threshold of 5 used for confident identification. However, comparison to the synthetic peptide is a clear match, comparable to the match shown in Figure 4F for the peptide detected in the immunopeptidome of the 786-O cell line.

6 **Figure S7.** ERV1514 is HIF2-responsive in 4/10 ccRCC cell lines, related to Figure 7 ERV1514 RNA level was examined by RT-qPCR in 10 ccRCC cell lines. The cell lines were treated with either 2 μM PT2399 or vehicle for 72 hours. *NDRG1* mRNA levels were included as controls. ERV RNA levels were normalized to *ACTB* RNA levels and then to the vehicle condition. For each cell line N=2–4.

7 **Figure S1.** Pharmacologic and Epigenetic Regulation of ERVs, Related to Figure 1 and 2 (A) ERV RNA levels of MGG-152 glioblastoma cells that were treated with 100 μM FG-4592 or vehicle for 72 hours. ERV RNA levels were normalized to *ACTB* RNA levels and then to cells treated with vehicle. (B) Immunoblot analysis of triple negative breast cancer cells (TNBC) (BT-549), melanoma cells (WM266–4), glioblastoma cells (GBM) (MGG152) and ccRCC cells (RCC4). Where indicated, the cells were treated with 100 μM FG-4592 for 72 hours. S.E. = short exposure. L.E. = long exposure. (C and E) ERV RNA levels in 786-O cells (C) or WM266–4 cells (E) that underwent CRISPR-based gene editing with one of two distinct SETDB1 sgRNAs (#g2 or #g4) (red bars) or a control sgRNA (sgCtrl) (blue bars). In (E) the cells were treated with 100 μM FG-4592 or vehicle for 72 hours. ERV RNA levels were normalized to *ACTB* RNA levels and then to sgCtrl cells treated with vehicle. (D and F) Immunoblots of cells as in (C and E). (G-L) RT-qPCR validation of HIF2-responsive ERVs in 786-O cells (G-I) or A-498 cells (J-L) that were stably infected with a virus encoding pVHL (VHL) or the empty vector (EV) (G and J), treated with 2 μM PT2399 (PT) or vehicle (Veh) for 72 hours (H and K), or that underwent CRISPR-based gene editing with a HIF2α sgRNA or a control sgRNA (sgCtrl) (I and L). *NDRG1* mRNA levels were included as controls. RNA levels were normalized to *ACTB* RNA levels and then to the EV, Veh or sgCtrl conditions. (G-I) Shown are the 20 ERVs that scored in two out of three high HIF2 versus low HIF2 comparisons by RNA-seq, as indicate by the “+”. (J-L) Shown are the 9 ERVs that scored in all three high HIF2 versus low HIF2 comparisons based on RNA-seq. (M-Q) 6 ERVs that scored as HIF2-responsive ERVs in both 786-O cells and A-498 cells are shown in the Venn Diagram (M) and were validated by RT-qPCR in multiple ccRCC cell lines (N-Q). In (N-Q) the cell lines were treated with either 2 μM PT2399 or vehicle for 72 hours. *NDRG1* mRNA levels were included as controls. ERV RNA levels were normalized to *ACTB* RNA levels and then to the vehicle condition. For panel A, N=6; for all the other panels, N=3.
**Table S1.** ERV quantification based on RNA-seq, Related to Figure 2

8 **Table S2.** ChIP-seq and PRO-seq analysis of ERV, Related to Figure 3

9 **Table S3.** ERV quantification based on Polysome-seq, Related to Figure 4

10 **Figure S4.** Studies Related to Identification of pVHL-responsive ERVs that are Translated and Presented as HLA-bound Peptides, Related to Figure 4 (A and C) Venn Diagrams showing pVHL-responsive translated ERVs based on Polysome-seq of 786-O cells (A) or A-498 cells (C) that were stably infected with a lentivirus encoding pVHL (VHL) or with the empty vector (EV). Note that in (A) 21 of the ERVs scored as pVHL-responsive Polysome-seq but had not scored as pVHL/HIF-responsive by RNA-seq in Figure 2. Of the remaining 5 ERVs, 4 scored as a pVHL/HIF-responsive in all 3 isogenic RNA-seq comparisons and 1 scored in 2 out of 3 of the isogenic RNA-seq experiments in Figure 2. (B and D) Polysome-associated ERV levels, as determined by RT-qPCR, in VHL and EV 786-O cells (B) and A-498 cells (D) for the ERVs that scored both by Polysome-seq and RNA-seq as pVHL/HIF-responsive in (A) and (C). cDNA derived from polysome-associated RNA was used as templates. ERV RNA levels were normalized to *ACTB* RNA levels and then to the EV cells. (E) Cell surface expression of HLA Class I proteins based on flow cytometry analysis of 786-O and A-498 cells that expressed pVHL or EV, and then were treated with either 100 ng/mL IFNγ or vehicle for 72 h. Class I proteins were detected with Alex647-conjugated antibodies compared to Alex647-conjugate isotype control IgG_2a_. (F,G,L,M) Immunoblot analysis (F and L) and ERV RNA levels (G and M) of cells as in (E). (G and M) *NDRG1* mRNA levels were included as controls. ERV RNA levels were normalized to *ACTB* RNA levels and then to the corresponding EV cells (with or without IFNγ). (H-K) RNA levels, as determined by normalized ERV counts in RNA-seq (H and J) and Polysome-seq (I and K) for ERVs capable of encoding Peptide KLIAGLIFLK (H and I) or ERVs capable of encoding ATFLGSLTGK (J and K). Peptide KLIAGLIFLK can be encoded by 28 homologous ERVs, while peptide ATFLGSLTGK can be encoded by 48 homologous ERVs. Shown in (H-K) are ERVs for which normalized ERV counts were non-zero in at least two out of the three replicates in EV cells. For all panels, N=3.
**Table S4.** LC-MS/MS analysis of ccRCC cell lines, Related to Figure 4

11 **Table S5.** ERV quantification based on patient scATAC-seq and scRNA-seq and RNA-seq, Related to Figure 5

12 **Figure S6.** ERV-derived Peptides Recognized by T cells from Human Kidney Cancer Patients, related to Figure 7 (A) Clinical information for RCC patients. N/A = not applicable. (B-F) Representative ELISpot images (B-D) and ELISpot quantification (spot count) (E-F) of IFNγ secreting PBMCs derived from allo-SCT RCC patients after stimulation with the indicated ERV-derived peptides. DMSO and an HIV peptide served as negative controls. CEF peptides and PHA served as positive controls. (B-D) Cell number plated in each well was listed on the right side of the images for patient 1, 2 and 4. Cell number varied because of differences in viable cell number after thawing. (E-F) Significance was determined by comparing the response to indicated peptides and to the HIV peptide for the corresponding patient. (N=3)

13 **Table S6.** LC-MS/MS analysis of ccRCC patient biospecimens, Related to Figure 6

14 **Table S7.** Peptide information for T-Scan assays, Related to Figure 7

## Figures and Tables

**Table:** Key resource table

REAGENT or RESOURCE	SOURCE	IDENTIFIER
**Antibodies**		
Rabbit monoclonal anti-HIF2α antibody	Cell Signaling Technology	Cat#7096, RRID: AB_10898028
Rabbit polyclonal anti-NDRG1 antibody	Cell Signaling Technology	Cat#5196, RRID: AB_10626626
Rabbit polyclonal anti-VHL antibody	Cell Signaling Technology	Cat#68547, RRID: AB_2716279
Rabbit monoclonal anti-HIF1α antibody	Cell Signaling Technology	Cat#14179, RRID: AB_2622225
Rabbit monoclonal anti-BNIP3 antibody	Cell Signaling Technology	Cat#44060, RRID: AB_2799259
Mouse monoclonal anti-α-Tubulin antibody	Sigma-Aldrich	Cat#T5168, RRID: AB_477579
Rabbit polyclonal anti-SETDB1 antibody	Proteintech	Cat#11231–1-AP, RRID: AB_2186069
Rabbit polyclonal anti-IRF-7 antibody	Cell Signaling Technology	Cat#4920, RRID: AB_2127551
Rabbit monoclonal anti-Rig-I antibody	Cell Signaling Technology	Cat#4200, RRID: AB_2175706
Peroxidase AffiniPure Goat anti-rabbit IgG	Jackson ImmunoResea rch Labs	Cat#111–035-003, RRID: AB_2313567
Peroxidase AffiniPure Goat anti-mouse IgG	Jackson ImmunoResea rch Labs	Cat#115–035-003, RRID: AB_10015289
FLAG	Sigma-Aldrich	Cat#F1804, RRID: AB_262044
HIF2α (Homemade)	Schodel et al.^101^	N/A
Carbonic Anhydrase 9 antibody, antihuman, PE, REAfinity	Miltenyi Biotec	Cat#130–123-299, RRID: AB_2857582
Mouse monoclonal anti-MHC class I (W6/32) AF647	Santa Cruz Biotechnology	Cat#sc-32235, RRID: AB_627934
Mouse monoclonal IgG2_a_ AF647	Santa Cruz Biotechnology	Cat#sc-24637, RRID: AB_737235
Mouse monoclonal anti-MHC class I (W6/32)	Santa Cruz Biotechnology	Cat#sc-32235, RRID: AB_627934
Rat Anti-Mouse IFNγ mAb (AN18), Unconjugated	Mabtech	Cat#3321–3-250, RRID: AB_907279
Mouse Anti-Human IFNγ mAb (1-D1K), Unconjugated	Mabtech	Cat#3420–3-250, RRID: AB_907283
Rat Anti-Mouse IFNγ mAb (R4–6A2), Biotin Conjugated	Mabtech	Cat#3321–6-250, RRID: AB_2280104
Mouse Anti-Human IFNγ mAb (7-B6–1), Biotin Conjugated	Mabtech	Cat#3420–6-250, RRID: AB_907273
CD3	BD Biosciences	Cat#553058, RRID: AB_394591
**Bacterial and virus strains**		
One Shot Stbl3 Competent Cells	Life Technologies	Cat#C737303
**Biological samples**		
Patient 16097101 tumor and PBMC biospecimen	NCT02950766	https://classic.clinicaltrials.gov/ct2/show/NCT02950766
Patient 16097102 tumor biospecimen	NCT02950766	https://classic.clinicaltrials.gov/ct2/show/NCT02950766
Patient 16097104 tumor and PBMC biospecimen	NCT02950766	https://classic.clinicaltrials.gov/ct2/show/NCT02950766
Patient 16097105 tumor, normal kidney and PBMC biospecimen	NCT02950766	https://classic.clinicaltrials.gov/ct2/show/NCT02950766
Patient 16097106 tumor, normal kidney and PBMC biospecimen	NCT02950766	https://classic.clinicaltrials.gov/ct2/show/NCT02950766
Patient 16097107 tumor, normal kidney and PBMC biospecimen	NCT02950766	https://classic.clinicaltrials.gov/ct2/show/NCT02950766
Patient 16097108 tumor biospecimen	NCT02950766	https://classic.clinicaltrials.gov/ct2/show/NCT02950766
Patient 16097109 tumor, normal kidney and PBMC biospecimen	NCT02950766	https://classic.clinicaltrials.gov/ct2/show/NCT02950766
Patient 16097110 tumor, normal kidney and PBMC biospecimen	NCT02950766	https://classic.clinicaltrials.gov/ct2/show/NCT02950766
Patient 16097111 tumor and PBMC biospecimen	NCT02950766	https://classic.clinicaltrials.gov/ct2/show/NCT02950766
Patient 16097112 tumor and PBMC biospecimen	NCT02950766	https://classic.clinicaltrials.gov/ct2/show/NCT02950766
Patient NHLBI-RCC-39 PBMC biospecimen	Provided by Dr. Richard W. Childs	N/A
Patient NHLBI-RCC-1 PBMC biospecimen	Provided by Dr. Richard W. Childs	N/A
Patient NHLBI-RCC-69 PBMC biospecimen	Provided by Dr. Richard W. Childs	N/A
Patient NHLBI-RCC-62 PBMC biospecimen	Provided by Dr. Richard W. Childs	N/A
Patient NHLBI-RCC-63 PBMC biospecimen	Provided by Dr. Richard W. Childs	N/A
Patient NHLBI-RCC-71 PBMC biospecimen	Provided by Dr. Richard W. Childs	N/A
ccRCC1 tumor, TILs, PBMC biospecimen	Provided by Tscan Therapeutics	N/A
ccRCC2 tumor, TILs, PBMC biospecimen	Provided by Tscan Therapeutics	N/A
ccRCC3 tumor, TILs, PBMC biospecimen	Provided by Tscan Therapeutics	N/A
ccRCC4 tumor, TILs, PBMC biospecimen	Provided by Tscan Therapeutics	N/A
ccRCC5 tumor, TILs, PBMC biospecimen	Provided by Tscan Therapeutics	N/A
ccRCC6 tumor, TILs, PBMC biospecimen	Provided by Tscan Therapeutics	N/A
ccRCC7 tumor, TILs, PBMC biospecimen	Provided by Tscan Therapeutics	N/A
**Chemicals, peptides, and recombinant proteins**		
PT2399	Peloton Therapeutics	N/A
FG-4592	ApexBio	Cat#A4187
GSK3685032	GSK	N/A
SYBR Green I Master Mix	Roche	Cat#04707516001
Disuccinimidyl glutarate	Thermo Fisher Scientific	Cat#20593
16% Formaldehyde (w/v)	Thermo Fisher Scientific	Cat#28908
RNase A	Invitrogen	Cat#AM2696
Proteinase K	Thermo Fisher Scientific	Cat#EO0492
Dithiothreitol	Thermo Fisher Scientific	Cat#707265ML
Biotin-11-UTP	Perkin Elmer	Cat#NEL543001EA
Ultrapure glycogen	Invitrogen	Cat#10814010
Recombinant Human IFNγ	Peprotech	Cat#300–02
Octyl β-d-glucopyranoside	Sigma-Aldrich	Cat#O8001
Iodoacetamide	Sigma-Aldrich	Cat#A3221
DNase I	Roche	Cat#4536282001
Phenylmethanesulfonyl Fluoride	Sigma-Aldrich	Cat#78830
2-Mercaptoethnol	Gibco	Cat#21985023
Collagenase D	Roche	Cat#1108858001
Dispase	STEMCELL Technologies	Cat#07913
DNase I	New England Biolabs	Cat#M0303L
Insulin	Sigma-Aldrich	Cat#I9278–5ML
EGF	Miltenyi Biotec	Cat#130–093-825
IL-7	Peprotech	200–07
IL-2	Peprotech	200–02
Hiltonol (Poly ICLC)	Oncovir	N/A
TMB substrate	Mabtech	Cat#3651–10
Phytohemagglutinin	Life Technologies	Cat#10576–015
KLIAGLIFL(K), K=lys (^13^C6, ^15^N2)	RS Synthesis	N/A
ATFLGSLTG(K), K=lys (^13^C6, ^15^N2)	RS Synthesis	N/A
ATFLGSLTW(K), K=lys (^13^C6, ^15^N2)	RS Synthesis	N/A
QLGGLVTF(K), K=lys (^13^C6, ^15^N2)	RS Synthesis	N/A
LIAGLIFLK	RS Synthesis	N/A
TALLWSLRK	RS Synthesis	N/A
QVLSLITNR	RS Synthesis	N/A
GLDLWIIGK	RS Synthesis	N/A
VLLDAGLR	RS Synthesis	N/A
APFPLSQIR	RS Synthesis	N/A
NLLSYLGKK	RS Synthesis	N/A
EEIIRKII	RS Synthesis	N/A
GQAGLKLLI	RS Synthesis	N/A
LPDKIPLA	RS Synthesis	N/A
LVILQHKAL	RS Synthesis	N/A
VEEASKFTL	RS Synthesis	N/A
VSASGTHTW	RS Synthesis	N/A
SLYNTVATL (HIV peptide)	RS Synthesis	N/A
CEF peptides	Mabtech	3618–1
SQEKDFHKV	Tscan Therapeutics	N/A
QEKDFHKVM	Tscan Therapeutics	N/A
HFFCGGISSV	Tscan Therapeutics	N/A
SQEKDFHKVM	Tscan Therapeutics	N/A
MSQEKDFHKV	Tscan Therapeutics	N/A
SVKVGQGIFTSF	Tscan Therapeutics	N/A
KDFHKVMSSL	Tscan Therapeutics	N/A
ARTGHLHFF	Tscan Therapeutics	N/A
YMCKSQGLQW	Tscan Therapeutics	N/A
RPDFRDHPW	Tscan Therapeutics	N/A
RPDFRDHPWMSL	Tscan Therapeutics	N/A
HPWMSLDWEL	Tscan Therapeutics	N/A
RPDFRDHPWM	Tscan Therapeutics	N/A
LPQGTSAQKA	Tscan Therapeutics	N/A
RPEGRPDFRDHPW	Tscan Therapeutics	N/A
FRDHPWMSL	Tscan Therapeutics	N/A
LPQGTSAQK	Tscan Therapeutics	N/A
DFRDHPWMSL	Tscan Therapeutics	N/A
KTKDLFLQL	Tscan Therapeutics	N/A
LIQGEIHKR	Tscan Therapeutics	N/A
SPKPVFQTF	Tscan Therapeutics	N/A
NVPIPELPGK	Tscan Therapeutics	N/A
KTKDLFLQLA	Tscan Therapeutics	N/A
RSPKPVFQTF	Tscan Therapeutics	N/A
LLIQGEIHK	Tscan Therapeutics	N/A
HSLNITSCY	Tscan Therapeutics	N/A
VPIPELPGK	Tscan Therapeutics	N/A
VPIPELPGKTK	Tscan Therapeutics	N/A
RLRVVPVHR	Tscan Therapeutics	N/A
WTRLPQGFK	Tscan Therapeutics	N/A
KVSCQRPRL	Tscan Therapeutics	N/A
GGTGSRPPK	Tscan Therapeutics	N/A
GFKNFPHHL	Tscan Therapeutics	N/A
HLWGGTGSRPPK	Tscan Therapeutics	N/A
GSRPPKVSCQR	Tscan Therapeutics	N/A
VTTHYTWTR	Tscan Therapeutics	N/A
GSRVRQRNR	Tscan Therapeutics	N/A
**Critical commercial assays**		
QIAshredder	Qiagen	Cat#79654
RNeasy Mini Kit	Qiagen	Cat#74106
AffinityScript qPCR cDNA Synthesis Kit	Agilent	Cat#600559
NEBNext Ultra Directional RNA Library Prep Kit	New England Biolabs	Cat#E7760
NEBNext rRNA Depletion Kit v2	New England Biolabs	Cat#E7405
KAPA RNA HyperPrep Kit with RiboErase	Roche	N/A
KAPA HiFi HotStart ReadyMix	Roche	Cat#KK2601
QIAseq FastSelect rRNA HMR	Qiagen	Cat#334375
PCR Purification Kit	Qiagen	Cat#28104
Qubit High Sensitivity Kit	Life Technologies	Cat#A33231
MinElute PCR Purification Kit	Qiagen	Cat#28004
Swift DNA Library Prep Kit	Swift Biosciences	N/A
Total RNA Purification Kit	Norgen Biotek Corp	Cat#17200
5’ DNA Adenylation Kit	New England Biolabs	Cat#M2611A
SMARTer Stranded Total RNA Sample Prep Kit - HI Mammalian	Takara	Cat#634873
RNeasy Micro Kit	Qiagen	Cat#74004
**Deposited data**		
RNA-seq_QJ8080 (HIF-responsive ERV in 786-O cells)	GEO	https://www.ncbi.nlm.nih.gov/geo/query/acc.cgi?acc=GSE253327
RNA-seq_QJ9511 (HIF-responsive ERV in A-498 cells)	GEO	https://www.ncbi.nlm.nih.gov/geo/query/acc.cgi?acc=GSE253373
RNA-seq_QJ10472 (DNMT1i derepressed ERV in 786-O cells)	GEO	https://www.ncbi.nlm.nih.gov/geo/query/acc.cgi?acc=GSE253328
ChIP-seq	GEO	https://www.ncbi.nlm.nih.gov/geo/query/acc.cgi?acc=GSE253325
PRO-seq	GEO	https://www.ncbi.nlm.nih.gov/geo/query/acc.cgi?acc=GSE253324
Polysome-seq	GEO	https://www.ncbi.nlm.nih.gov/geo/query/acc.cgi?acc=GSE253323
Original mass spectra, peptide spectrum matches, protein sequence databases	MassIVE	ftp://MSV000093874@massive.ucsd.edu with username: MSV000093874 and password: HIFresponsiveERV
**Experimental models: Cell lines**		
786-O	ATCC	CRL-1932
A-498	ATCC	HTB-44
769-P	ATCC	CRL-1933
Caki-2	ATCC	HTB-47
HEK293T	ATCC	CRL-11268
BT-549	ATCC	HTB-122
RCC4	Provided by Dr. Peter Ratcliffe (University of Oxford and Francis Click Institute)	N/A
OS-RC-2	RIKEN Bioresource Research Center Cell Bank	RCB0735
TUHR4TKB	RIKEN Bioresource Research Center Cell Bank	RCB1198
WM266–4	Provided by Dr. Rizwan Haq (Dana-Farber Cancer Institute) and Dr. David E. Fisher (Massachusett s General Hospital)	N/A
MGG152	Wakimoto et al.^95^	N/A
UM-RC-2	Provided by Dr. Bert Zbar and Marston Linehan (National Cancer Institute)	N/A
A-704	ATCC	HTB-45
TUHR14TKB	RIKEN Bioresource Research Center Cell Bank	RCB1383
**Experimental models: Organisms/strains**		
CB6F1-Tg(HLA-A*1101/H2-Kb)A11.01	Taconic	Cat#9660-F
**Oligonucleotides**		
sgCtrl: CTTCCGCGGCCCGTTCAA	Stransky et al.^52^	N/A
sgAAVS1: GGGGCCACTAGGGACAGGAT	Wang et al.^53^	N/A
sgHIF2α: TCATGAGGATGAAGTGCA	Stransky et al.^52^	N/A
sgSETDB1#g2: AAGGAAAGAGTCTACTGTCG	This paper	N/A
sgSETDB1#g4: TTGTTACACTCATATACCCT	This paper	N/A
sgHIF1α: GAACTCACATTATGTGGAAG	This paper	N/A
ERVE-4 (RT-qPCR) fwd: GCAGATCCTGGGAGCACTCT	Braun et al.^10^	N/A
ERVE-4 (RT-qPCR) rev: TGTTCAACCGCTGTGTTAATTCTC	Braun et al.^10^	N/A
ERV3.2 (RT-qPCR) fwd: CAAGAGGCGGCATAGAAGCAA	Panda et al.^34^	N/A
ERV3.2 (RT-qPCR) rev: GGAGAGTAGCTTGGGGTTTCA	Panda et al.^34^	N/A
ERV4700 (RT-qPCR) fwd: GACGCTCCCAGCAGAATAAA	Braun et al.^10^	N/A
ERV4700 (RT-qPCR) rev: CCGGTCAGGAAACCAAGAAA	Braun et al.^10^	N/A
ACTB (RT-qPCR) fwd: ACCAACTGGGACGACATGGAGAAA	Nicholson et al.^97^	N/A
ACTB (RT-qPCR) rev: TAGCACAGCCTGGATAGCAACGTA	Nicholson et al.^97^	N/A
2112 (RT-qPCR) fwd: TTCTGGCCAAAATTCGGGTC	This paper	N/A
2112 (RT-qPCR) rev: TTTGGTAGGGGAAGGTGGTC	This paper	N/A
2948 (RT-qPCR) fwd: TCCATTCTTGCCACCCTGAT	This paper	N/A
2948 (RT-qPCR) rev: AGGGGACGGTGTAGGAAAAG	This paper	N/A
3797 (RT-qPCR) fwd: ATGTGATGCCCGATACCTTC	This paper	N/A
3797 (RT-qPCR) rev: CTAGATTTGGACTGCCCTTACG	This paper	N/A
3888 (RT-qPCR) fwd: CTGAGACCACAGCACCTACA	This paper	N/A
3888 (RT-qPCR) rev: AGGCGGAAGGTTGATACACA	This paper	N/A
4377 (RT-qPCR) fwd: GAAATGCATTGACGCTTGGC	This paper	N/A
4377 (RT-qPCR) rev: GCCCTCAAACCTCACAACAG	This paper	N/A
4721 (RT-qPCR) fwd: ACTCATGGCAGTGGTGATCA	This paper	N/A
4721 (RT-qPCR) rev: CGAGCAACACCTGTCAAACA	This paper	N/A
475 (RT-qPCR) fwd: TGGCCAGCATTAGAGGTAGG	This paper	N/A
475 (RT-qPCR) rev: CTCTCAACCACGGTGTGTTG	This paper	N/A
4818 (RT-qPCR) fwd: TGAGAAGTTTTGTCTGCGGC	This paper	N/A
4818 (RT-qPCR) rev: GGGAGTGCTCTTTGGATCCT	This paper	N/A
504 (RT-qPCR) fwd: ACATCTCCAGCACACAAGGA	This paper	N/A
504 (RT-qPCR) rev: TGCCGGGTGAGTTTGATAGT	This paper	N/A
5048 (RT-qPCR) fwd: TGGGCCTTAGAATGTCTGCA	This paper	N/A
5048 (RT-qPCR) rev: AGCATCAGTCTTCAGCCACT	This paper	N/A
506 (RT-qPCR) fwd: CCCCTTAGCCTCTGTTGTCA	This paper	N/A
506 (RT-qPCR) rev: GACATCAGGCACCTCAGACT	This paper	N/A
5147 (RT-qPCR) fwd: TTAAGGGGACAGACAGGCAG	This paper	N/A
5147 (RT-qPCR) rev: GGCCAGTGGAGTAAGAACCT	This paper	N/A
565 (RT-qPCR) fwd: ATACTTCCTGGGGTTGGGTG	This paper	N/A
565 (RT-qPCR) rev: AGAGAAGTCTGACCTGTGGC	This paper	N/A
5875 (RT-qPCR) fwd: TCCCTGCCATGAGAGACAAG	This paper	N/A
5875 (RT-qPCR) rev: ATATCCTGAAGTCCCTGCGG	This paper	N/A
NDRG1 (RT-qPCR) fwd: CTCCTGCAAGAGTTTGATGTCC	Cho et al.^43^	N/A
NDRG1 (RT-qPCR) rev: TCATGCCGATGTCATGGTAGG	Cho et al.^43^	N/A
1066 (RT-qPCR) fwd: GATTTCCTCCTCCCCACCAA	This paper	N/A
1066 (RT-qPCR) rev: CCTGAGTACTCCACACTGCA	This paper	N/A
1166 (RT-qPCR) fwd: ACAAGGAGACGAGACAAGCA	This paper	N/A
1166 (RT-qPCR) rev: GAAAGGGGATGGAGTGGGAA	This paper	N/A
1271 (RT-qPCR) fwd: AAATACGTACGTGCATGGCC	This paper	N/A
1271 (RT-qPCR) rev: AACCTGCCCTTTGTATCCCA	This paper	N/A
1347 (RT-qPCR) fwd: AAGGGAGGAGGCTACACAAC	This paper	N/A
1347 (RT-qPCR) rev: TGGGTTTTGGGGATACTGCT	This paper	N/A
1693 (RT-qPCR) fwd: AAAAGTTCAGCCTGGCCATG	This paper	N/A
1693 (RT-qPCR) rev: TGTGATGGGAGTGACAGCTT	This paper	N/A
2852 (RT-qPCR) fwd: TGGCAGTAAACCCCACAGAA	This paper	N/A
2852 (RT-qPCR) rev: GAGGGAGGGAGGCCTTATTC	This paper	N/A
3136 (RT-qPCR) fwd: GCTGTTTGGAGTCTTTGGCA	This paper	N/A
3136 (RT-qPCR) rev: ATGGTGACTTGACGACCCAT	This paper	N/A
3254 (RT-qPCR) fwd: GCACAGCGACCAGACTAAAG	This paper	N/A
3254 (RT-qPCR) rev: GCCCAGTAATCCATGCAGTG	This paper	N/A
3920 (RT-qPCR) fwd: TCTTTTCCGGCGCATTTTGA	This paper	N/A
3920 (RT-qPCR) rev: CCACCCCTCCTCAAAATCCT	This paper	N/A
4054 (RT-qPCR) fwd: CTGCGCAGTCAGTAAGTCAC	This paper	N/A
4054 (RT-qPCR) rev: GTCCCTTAATTGAGCCTGCG	This paper	N/A
6281 (RT-qPCR) fwd: CCTTCACATCCTCCCCTTGT	This paper	N/A
6281 (RT-qPCR) rev: TTTAAAGCGTGCTGTGGGAC	This paper	N/A
876 (RT-qPCR) fwd: TCTCAAAGTCACGTCGTCCA	This paper	N/A
876 (RT-qPCR) rev: CCAGAGACAGGGAGTGGTTT	This paper	N/A
6056 (RT-qPCR) fwd: AGTCTGAGCTTCTTGGACCC	This paper	N/A
6056 (RT-qPCR) rev: CAGTGGCACCAAGGACTAGA	This paper	N/A
3177 (RT-qPCR) fwd: TGATGCCAGAAATGGGTCCT	This paper	N/A
3177 (RT-qPCR) rev: TGTGTCCCATCAATCCACCA	This paper	N/A
3895 (RT-qPCR) fwd: TGGGCTAGTCAGTGTCACTC	This paper	N/A
3895 (RT-qPCR) rev: ACTGTGGCACTACTAGCTGT	This paper	N/A
4862 (RT-qPCR) fwd: AATCAACGAGGTGGTGGCTA	This paper	N/A
4862 (RT-qPCR) rev: ACTGAGATGGGCCCAATCAA	This paper	N/A
5682 (RT-qPCR) fwd: GTTTCCCATCTGTGCAGGAC	This paper	N/A
5682 (RT-qPCR) rev: GCGATTCGGCAGTATCAGTC	This paper	N/A
6084 (RT-qPCR) fwd: CCCCTGTGTATTACGCTTGC	This paper	N/A
6084 (RT-qPCR) rev: GGAGGCAGCAGGAGTAGTAG	This paper	N/A
838 (RT-qPCR) fwd: AAAACCGCATCCAGTCCAAC	This paper	N/A
838 (RT-qPCR) rev: TGTTGGGGTGGCGAAAATTT	This paper	N/A
862 (RT-qPCR) fwd: TCAATCCAGCCTCCCACATT	This paper	N/A
862 (RT-qPCR) rev: ATGAGAGGTTCTAAGGGGCG	This paper	N/A
1745 (RT-qPCR) fwd: AGGACAGTGGGCGAAAAGTA	This paper	N/A
1745 (RT-qPCR) rev: TGTTGATCTGCGGGGTGTAT	This paper	N/A
2651 (RT-qPCR) fwd: CCTGGAGACTCTGTTTGGGT	This paper	N/A
2651 (RT-qPCR) rev: AGAGCAGGGCAGTCATCTTT	This paper	N/A
2581 (RT-qPCR) fwd: GCAATTCAGACTGGCCACAA	This paper	N/A
2581 (RT-qPCR) rev: CGAGTTGGGAGGAGAAAGGT	This paper	N/A
4757 (RT-qPCR) fwd: GCCTATACAGCCTCCTCCAG	This paper	N/A
4757 (RT-qPCR) rev: GTGGATTGTTGGGATCAGGC	This paper	N/A
EPAS1-N-sg1 (FLAG-HA-Knockin): AGCGACAATGACAGCTGACA	This paper	N/A
3XFLAG-HA-10-N-EPAS1 (FLAG-HAKnockin HDR Template): ACACTCGCGAGCGGACCGCCACACGG GTCCGGTGCCCGCTGCGCTTCCGCCC CAGCGCTCCTGAGGCGGCCGTACAAT CCTCGGCAGTGTCCTGAGACTGTATG GTCAGCTCAGCCCGGCCTCCGACTCC TTCCGACTCCCAGCATTCGAGCCACTT TTTTTTTTCTTTGAAAACTCAGAAAAGT GACTCCTTTTCCAGGGAAAAAGGAACT TGGGTTCCCTTCTCTCCGTCCTCTTTT CGGGTCTGACAGCCTCCACCCACTCC TTCCCCGGACCCCGCCTCCGCGCGCA GGTTCCTCCCAGTCACCTTTCTCCACC CCCGCCCCCGCACCTAGCCCGCCGCG CGCCACCTTCCACCTGACTGCGCGGG GCGCTCGGGACCTGCGCGCACCTCGG ACCTTCACCACCCGCCCGGGCCGCGG GGAGCGGACGAGGGCCACAGCCCCC CACCCGCCAGGGAGCCCAGGTGCTCG GCGTCTGAACGTCTCAAAGGGCCACA GCGACAATGGactacaaagaccatgacggtgatt ataaagatcatgacatcgattacaaggatgacgatgac aagGGCGGC**tacccatacgatgttccagattacg ctGGCGGCGGCGGCTCCGGCGGCGGC GGCTCC**ACAGCAGATAAAGAGAAGAAA AGgtaagcgggcgtccgggccgatcagggggccggt ccgaggccagggccgggctgcgcggggcaggcgcg accgagagtggttgggagaagagtgctgagaggtcttc ggagctcgaggctcggtttggagggctgtgagggggag agcatgtgcccggtttgggggcgcggatcagcagctttg	This paper	N/A
cagtggagacttctgcggctcggaggagtcggggatttg cgcgcacggcgaggccaggaggccgagggagatgg cctggaggtcggaggtgcttccgcggtgcctttgaaaatc tcccagccgccccgcagcagattaccgtggccaccgg cgcttggaaatgtttgtgggtgcatgtcctcgactttctctgg tctctcattttggggcagcatccacgtctctatttttctctgga ttcgcggagtccttccaaatgcgctcctgtgcccgcgccg cggcccaagtgggcaaag		
EPAS1 (Amplicon sequencing) fwd: GCGCGCCACCTTCCACCTGA	This paper	N/A
EPAS1 (Amplicon sequencing) rev: CGTGCGCGCAAATCCCCGACT	This paper	N/A
EPAS1 with HDR (Amplicon sequence, 550 bp): GCGCGCCACCTTCCACCTGACTGCGC GGGGCGCTCGGGACCTGCGCGCACCT CGGACCTTCACCACCCGCCCGGGCCG CGGGGAGCGGACGAGGGCCACAGCC CCCCACCCGCCAGGGAGCCCAGGTGC TCGGCGTCTGAACGTCTCAAAGGGCC ACAGCGACAATGGactacaaagaccatgacgg tgattataaagatcatgacatcgattacaaggatgacgat gacaagGGCGGC**tacccatacgatgttccagatt acgctGGCGGCGGCGGCTCCGGCGGC GGCGGCTCC**ACAGCAGATAAAGAGAA GAAAAGgtaagcgggcgtccgggccgatcagggg gccggtccgaggccagggccgggctgcgcggggcag gcgcgaccgagagtggttgggagaagagtgctgagag gtcttcggagctcgaggctcggtttggagggctgtgagg gggagagcatgtgcccggtttgggggcgcggatcagc agctttgcagtggagacttctgcggctcggaggagtcgg ggatttgcgcgcac	This paper	N/A
EPAS1 with no HDR (Amplicon sequence, 421 bp): GCGCGCCACCTTCCACCTGACTGCGC GGGGCGCTCGGGACCTGCGCGCACCT CGGACCTTCACCACCCGCCCGGGCCG CGGGGAGCGGACGAGGGCCACAGCC CCCCACCCGCCAGGGAGCCCAGGTGC TCGGCGTCTGAACGTCTCAAAGGGCC ACAGCGACAATGACAGCTGACAAGGA GAAGAAAAGgtaagcgggcgtccgggccgatca gggggccggtccgaggccagggccgggctgcgcggg gcaggcgcgaccgagagtggttgggagaagagtgctg agaggtcttcggagctcgaggctcggtttggagggctgt gagggggagagcatgtgcccggtttgggggcgcggat cagcagctttgcagtggagacttctgcggctcggaggag tcggggatttgcgcgcacg	This paper	N/A
EGLN3 (ChIP-qPCR) fwd: AGTGTCCGTTCCCAGCTCAG	Schodel et al.101	N/A
EGLN3 (ChIP-qPCR) rev: TAGGCACAGTAAACAGGCC	Schodel et al.101	N/A
ACTB (ChIP-qPCR) fwd: ACCATGGATGATGATATCGCC	Schodel et al.101	N/A
ACTB (ChIP-qPCR) rev: GCCTTGCACATGCCGG	Schodel et al.101	N/A
ERVE-4 HRE1 (ChIP-qPCR) fwd: GGAGCACTCTCGCGTAGG	This paper	N/A
ERVE-4 HRE1 (ChIP-qPCR) rev: GATCAGCCCACACTTCCACT	This paper	N/A
ERVE-4 HRE 2 (ChIP-qPCR) fwd: TGACCTAACCGCTTGTGGAT	This paper	N/A
ERVE-4 HRE 2 (ChIP-qPCR) rev: AACTCTGAGGGGTCCATGTG	This paper	N/A
4818 HRE 1 (ChIP-qPCR) fwd: GCTCCCCGAATGCACAATTT	This paper	N/A
4818 HRE 1 (ChIP-qPCR) rev: TGAAACTCTCTGCCCTGTTCT	This paper	N/A
4818 HRE 2 (ChIP-qPCR) fwd: ACAGGGCAGAGAGTTTCACA	This paper	N/A
4818 HRE 2 (ChIP-qPCR) rev: GACCCTCCCGTATCGCTTTA	This paper	N/A
5875 HRE 1 (ChIP-qPCR) fwd: TCCCCAAGAGAGCAATTCCT	This paper	N/A
5875 HRE 1 (ChIP-qPCR) rev: GAAACTCCCTGCCCTGTTCT	This paper	N/A
5875 HRE 2 (ChIP-qPCR) fwd: CGAGTGAGCAATTCCTGTCC	This paper	N/A
5875 HRE 2 (ChIP-qPCR) rev: CTGCCTGACCTAAACCCAGT	This paper	N/A
ERV3.2 (ChIP-qPCR) fwd: TGTTTCTCCCTGACCATCGG	This paper	N/A
ERV3.2 (ChIP-qPCR) rev: GCCACAGACAAAACCTCTCA	This paper	N/A
3797 (ChIP-qPCR) fwd: CTACTCACTTGTGGCATGCC	This paper	N/A
3797 (ChIP-qPCR) rev: AGGAGGCTGTGAAGATGGTC	This paper	N/A
4377 (ChIP-qPCR) fwd: AGATTGAAGGGTGGCACTGA	This paper	N/A
4377 (ChIP-qPCR) rev: AATCCACCTACAACCTCGGG	This paper	N/A
4721 (ChIP-qPCR) fwd: AGAAAGTCCTGAGAGCGGA	This paper	N/A
4721 (ChIP-qPCR) rev: AAGAGCGAATAACCCTCCCC	This paper	N/A
1514 (RT-qPCR) fwd: CGTGCTGAGGGATGGGATAT	This paper	N/A
1514 (RT-qPCR) rev: TCCCAATCCAAAGCCTCCTT	This paper	N/A
**Recombinant DNA**		
pLenti-EF1α-cas9-FLAG-IRES-Neo	Nicholson et al.97	N/A
pLX304-EV-IRES-GFP	Addgene	187640
pLX304-VHL-IRES-Tdtomato	Addgene	187643
pLenti-EF1α-HIF2α dPA (P405A, P531A)	Addgene	232954
pLentiCRISPRv2-Puro	Addgene	98290
psPAX2	Addgene	12260
pMD2.G	Addgene	12259
**Software and algorithms**		
GraphPad Prism	Dotmatics	https://www.graphpad.com/features
ImageJ	NIH	https://imagej.net
Biorender	Biorender	https://www.biorender.com
R	The R Project for Statistical Computing	https://www.r-project.org/
IGV: Integrative Genomics Viewer	Broad Institute	https://www.igv.org
FlowJo	BD Biosciences	https://www.flowjo.com/solutions/flowjo
Spectrum Mill MS Proteomics Software	Broad Institute	https://proteomics.broadinstitute.org, Version 8.02
Orbitrap Exploris 480 Xcalibur	Thermo Scientific	1.1–1.1.75.22/1.1.117.22 2.0–2.0.122.16/2.0.182.25 3.0–3.0.204.12/3.0.261.13 3.1–3.1.231.6/3.1.279.9
Orbitrap Fusion Lumos Xcalibur	Thermo Scientific	3.1.2412.25
HLAthena binding prediction	Broad Institute	http://hlathena.tools/
**Other**		
Adaptive Forced Acoustics (AFA) fiber milliTUBE	Covaris	Cat#520135
ART^™^ Wide Bore Filtered Pipette Tips	Thermo Fisher Scientific	Cat#2079GPK
Round-Bottom Polystyrene Test Tubes with Cell Strainer Snap Cap, 5mL	Falcon	Cat#352235
T4 RNA Ligase 2, truncated KQ	New England Biolabs	Cat#M0373
mRNA Decapping Enzyme	New England Biolabs	Cat#M0608S
T4 RNA Ligase 1	New England Biolabs	Cat#M0204L
SuperScript IV Reverse Transcriptase	Invitrogen	Cat#18090010
NEBNext Ultra II Q5 Master Mix	New England Biolabs	Cat#M0544S
Low Protein Binding Microcentrifuge Tubes	Thermo Fisher Scientific	Cat#90410
Dynabeads Streptavidin C1	Invitrogen	Cat#65001
Betaine	Sigma-Aldrich	Cat#61962
Versene	Gibco	Cat#15040066
Human TruStain FcX	Biolegend	Cat#422302
Fixation Buffer	Biolegend	Cat#420801
Fisher 150 homogenizer	Thermo Fisher Scientific	Cat#15–340-176
GammaBind Plus Sepharose antibody purification resin	Cytiva	Cat#17088601
Human Serum	Sigma-Aldrich	Cat#H4522
Mycobacterium Tuberculosis H37Ra	BD Biosciences Difco	DF3114–33-8
BD Difco Complete Freund’s adjuvant	BD Biosciences Difco	DF0638–60-7
ELISpot Plate	Millipore	Cat#MSIPS4W10
Streptavidin-HRP	Mabtech	Cat#3310–9-1000

## Highlights

HIF2 transcriptionally upregulates many relatively intact ERVs in kidney cancers

Some of these ERVs are translated and presented as HLA-bound peptides

T Cells against such peptides are detectable after successful kidney cancer immunotherapy

ERV expression can be induced in non-kidney cancer cell lines with a HIF agonist

## References

[1] Postow MA, Callahan MK, and Wolchok JD (2015). Immune Checkpoint Blockade in Cancer Therapy. J Clin Oncol 33, 1974–1982. 10.1200/JCO.2014.59.4358. 25605845 PMC4980573

[2] Baumeister SH, Freeman GJ, Dranoff G, and Sharpe AH (2016). Coinhibitory Pathways in Immunotherapy for Cancer. Annu Rev Immunol 34, 539–573. 10.1146/annurev-immunol-032414-112049. 26927206

[3] Sharma P, Siddiqui BA, Anandhan S, Yadav SS, Subudhi SK, Gao J, Goswami S, and Allison JP (2021). The Next Decade of Immune Checkpoint Therapy. Cancer Discov 11, 838–857. 10.1158/2159-8290.CD-20-1680. 33811120

[4] Rizvi NA, Hellmann MD, Snyder A, Kvistborg P, Makarov V, Havel JJ, Lee W, Yuan J, Wong P, Ho TS, (2015). Cancer immunology. Mutational landscape determines sensitivity to PD-1 blockade in non-small cell lung cancer. Science 348, 124–128. 10.1126/science.aaa1348. 25765070 PMC4993154

[5] Yarchoan M, Hopkins A, and Jaffee EM (2017). Tumor Mutational Burden and Response Rate to PD-1 Inhibition. N Engl J Med 377, 2500–2501. 10.1056/NEJMc1713444. 29262275 PMC6549688

[6] Lawrence MS, Stojanov P, Polak P, Kryukov GV, Cibulskis K, Sivachenko A, Carter SL, Stewart C, Mermel CH, Roberts SA, (2013). Mutational heterogeneity in cancer and the search for new cancer-associated genes. Nature 499, 214–218. 10.1038/nature12213. 23770567 PMC3919509

[7] Xu J, Lv J, Zhuang Q, Yang Z, Cao Z, Xu L, Pei P, Wang C, Wu H, Dong Z, (2020). A general strategy towards personalized nanovaccines based on fluoropolymers for post-surgical cancer immunotherapy. Nat Nanotechnol 15, 1043–1052. 10.1038/s41565-020-00781-4. 33139933

[8] Turajlic S, Litchfield K, Xu H, Rosenthal R, McGranahan N, Reading JL, Wong YNS, Rowan A, Kanu N, Al Bakir M, (2017). Insertion-and-deletion-derived tumour-specific neoantigens and the immunogenic phenotype: a pan-cancer analysis. Lancet Oncol 18, 1009–1021. 10.1016/S1470-2045(17)30516–8. 28694034

[9] McDermott DF, Huseni MA, Atkins MB, Motzer RJ, Rini BI, Escudier B, Fong L, Joseph RW, Pal SK, Reeves JA, (2018). Clinical activity and molecular correlates of response to atezolizumab alone or in combination with bevacizumab versus sunitinib in renal cell carcinoma. Nat Med 24, 749–757. 10.1038/s41591-018-0053-3. 29867230 PMC6721896

[10] Braun DA, Hou Y, Bakouny Z, Ficial M, Sant’ Angelo M, Forman J, Ross-Macdonald P, Berger AC, Jegede OA, Elagina L, (2020). Interplay of somatic alterations and immune infiltration modulates response to PD-1 blockade in advanced clear cell renal cell carcinoma. Nat Med 26, 909–918. 10.1038/s41591-020-0839-y. 32472114 PMC7499153

[11] Motzer RJ, Robbins PB, Powles T, Albiges L, Haanen JB, Larkin J, Mu XJ, Ching KA, Uemura M, Pal SK, (2020). Avelumab plus axitinib versus sunitinib in advanced renal cell carcinoma: biomarker analysis of the phase 3 JAVELIN Renal 101 trial. Nat Med 26, 1733–1741. 10.1038/s41591-020-1044-8. 32895571 PMC8493486

[12] Lokich J. (1997). Spontaneous regression of metastatic renal cancer. Case report and literature review. Am J Clin Oncol 20, 416–418. 10.1097/00000421-199708000-00020. 9256902

[13] Negrier S, Escudier B, Lasset C, Douillard JY, Savary J, Chevreau C, Ravaud A, Mercatello A, Peny J, Mousseau M, (1998). Recombinant human interleukin-2, recombinant human interferon alfa-2a, or both in metastatic renal-cell carcinoma. Groupe Francais d’Immunotherapie. N Engl J Med 338, 1272–1278. 10.1056/NEJM199804303381805. 9562581

[14] McDermott DF, and Atkins MB (2004). Application of IL-2 and other cytokines in renal cancer. Expert Opin Biol Ther 4, 455–468. 10.1517/14712598.4.4.455. 15102596

[15] Choueiri TK, and Motzer RJ (2017). Systemic Therapy for Metastatic Renal-Cell Carcinoma. N Engl J Med 376, 354–366. 10.1056/NEJMra1601333. 28121507

[16] Rooney MS, Shukla SA, Wu CJ, Getz G, and Hacohen N. (2015). Molecular and genetic properties of tumors associated with local immune cytolytic activity. Cell 160, 48–61. 10.1016/j.cell.2014.12.033. 25594174 PMC4856474

[17] Bruni D, Angell HK, and Galon J. (2020). The immune contexture and Immunoscore in cancer prognosis and therapeutic efficacy. Nat Rev Cancer 20, 662–680. 10.1038/s41568-020-0285-7. 32753728

[18] Kaelin WG Jr. (2022). Von Hippel-Lindau disease: insights into oxygen sensing, protein degradation, and cancer. J Clin Invest 132. 10.1172/JCI162480. PMC9479583 36106637

[19] Kaelin WG Jr. (2008). The von Hippel-Lindau tumour suppressor protein: O2 sensing and cancer. Nat Rev Cancer 8, 865–873. 18923434 10.1038/nrc2502

[20] Choueiri TK, and Kaelin WG Jr. (2020). Targeting the HIF2-VEGF axis in renal cell carcinoma. Nat Med 26, 1519–1530. 10.1038/s41591-020-1093-z. 33020645

[21] Jonasch E, Donskov F, Iliopoulos O, Rathmell WK, Narayan VK, Maughan BL, Oudard S, Else T, Maranchie JK, Welsh SJ, (2021). Belzutifan for Renal Cell Carcinoma in von Hippel-Lindau Disease. N Engl J Med 385, 2036–2046. 10.1056/NEJMoa2103425. 34818478 PMC9275515

[22] Choueiri TK, Powles T, Peltola K, de Velasco G, Burotto M, Suarez C, Ghatalia P, Iacovelli R, Lam ET, Verzoni E, (2024). Belzutifan versus Everolimus for Advanced Renal-Cell Carcinoma. N Engl J Med 391, 710–721. 10.1056/NEJMoa2313906. 39167807

[23] Lander ES, Linton LM, Birren B, Nusbaum C, Zody MC, Baldwin J, Devon K, Dewar K, Doyle M, FitzHugh W, (2001). Initial sequencing and analysis of the human genome. Nature 409, 860–921. 10.1038/35057062. 11237011

[24] Li WH, Gu Z, Wang H, and Nekrutenko A. (2001). Evolutionary analyses of the human genome. Nature 409, 847–849. 10.1038/35057039. 11237007

[25] Jansz N, and Faulkner GJ (2021). Endogenous retroviruses in the origins and treatment of cancer. Genome Biol 22, 147. 10.1186/s13059-021-02357-4. 33971937 PMC8108463

[26] Saini SK, Orskov AD, Bjerregaard AM, Unnikrishnan A, Holmberg-Thyden S, Borch A, Jensen KV, Anande G, Bentzen AK, Marquard AM, (2020). Human endogenous retroviruses form a reservoir of T cell targets in hematological cancers. Nat Commun 11, 5660. 10.1038/s41467-020-19464-8. 33168830 PMC7653045

[27] Bonaventura P, Alcazer V, Mutez V, Tonon L, Martin J, Chuvin N, Michel E, Boulos RE, Estornes Y, Valladeau-Guilemond J, (2022). Identification of shared tumor epitopes from endogenous retroviruses inducing high-avidity cytotoxic T cells for cancer immunotherapy. Sci Adv 8, eabj3671. 10.1126/sciadv.abj3671. PMC8791462 35080970

[28] Feinberg AP, and Vogelstein B. (1983). Hypomethylation distinguishes genes of some human cancers from their normal counterparts. Nature 301, 89–92. 10.1038/301089a0. 6185846

[29] Wang-Johanning F, Radvanyi L, Rycaj K, Plummer JB, Yan P, Sastry KJ, Piyathilake CJ, Hunt KK, and Johanning GL (2008). Human endogenous retrovirus K triggers an antigen-specific immune response in breast cancer patients. Cancer Res 68, 5869–5877. 10.1158/0008-5472.CAN-07-6838. 18632641 PMC5802396

[30] Weyerer V, Strissel PL, Stohr C, Eckstein M, Wach S, Taubert H, Brandl L, Geppert CI, Wullich B, Cynis H, (2021). Endogenous Retroviral-K Envelope Is a Novel Tumor Antigen and Prognostic Indicator of Renal Cell Carcinoma. Front Oncol 11, 657187. 10.3389/fonc.2021.657187. PMC8100683 33968761

[31] Krug B, De Jay N, Harutyunyan AS, Deshmukh S, Marchione DM, Guilhamon P, Bertrand KC, Mikael LG, McConechy MK, Chen CCL, (2019). Pervasive H3K27 Acetylation Leads to ERV Expression and a Therapeutic Vulnerability in H3K27M Gliomas. Cancer Cell 35, 782–797 e788. 10.1016/j.ccell.2019.04.004. 31085178 PMC6521975

[32] Zhao S, Lu J, Pan B, Fan H, Byrum SD, Xu C, Kim A, Guo Y, Kanchi KL, Gong W, (2023). TNRC18 engages H3K9me3 to mediate silencing of endogenous retrotransposons. Nature 623, 633–642. 10.1038/s41586-023-06688-z. 37938770 PMC11000523

[33] Smith CC, Beckermann KE, Bortone DS, De Cubas AA, Bixby LM, Lee SJ, Panda A, Ganesan S, Bhanot G, Wallen EM, (2018). Endogenous retroviral signatures predict immunotherapy response in clear cell renal cell carcinoma. J Clin Invest 128, 4804–4820. 10.1172/JCI121476. 30137025 PMC6205406

[34] Panda A, de Cubas AA, Stein M, Riedlinger G, Kra J, Mayer T, Smith CC, Vincent BG, Serody JS, Beckermann KE, (2018). Endogenous retrovirus expression is associated with response to immune checkpoint blockade in clear cell renal cell carcinoma. JCI Insight 3. 10.1172/jci.insight.121522. PMC6141170 30135306

[35] de Cubas AA, Dunker W, Zaninovich A, Hongo RA, Bhatia A, Panda A, Beckermann KE, Bhanot G, Ganesan S, Karijolich J, and Rathmell WK (2020). DNA hypomethylation promotes transposable element expression and activation of immune signaling in renal cell cancer. JCI Insight 5. 10.1172/jci.insight.137569. PMC7308050 32493845

[36] Ficial M, Jegede OA, Sant’Angelo M, Hou Y, Flaifel A, Pignon JC, Braun DA, Wind-Rotolo M, Sticco-Ivins MA, Catalano PJ, (2021). Expression of T-Cell Exhaustion Molecules and Human Endogenous Retroviruses as Predictive Biomarkers for Response to Nivolumab in Metastatic Clear Cell Renal Cell Carcinoma. Clin Cancer Res 27, 1371–1380. 10.1158/1078-0432.CCR-20-3084. 33219016 PMC8443005

[37] Childs R, Chernoff A, Contentin N, Bahceci E, Schrump D, Leitman S, Read EJ, Tisdale J, Dunbar C, Linehan WM, (2000). Regression of metastatic renal-cell carcinoma after nonmyeloablative allogeneic peripheral-blood stem-cell transplantation. N Engl J Med 343, 750–758. 10.1056/NEJM200009143431101. 10984562

[38] Takahashi Y, Harashima N, Kajigaya S, Yokoyama H, Cherkasova E, McCoy JP, Hanada K, Mena O, Kurlander R, Tawab A, (2008). Regression of human kidney cancer following allogeneic stem cell transplantation is associated with recognition of an HERV-E antigen by T cells. J Clin Invest 118, 1099–1109. 10.1172/JCI34409. 18292810 PMC2248804

[39] Cherkasova E, Scrivani C, Doh S, Weisman Q, Takahashi Y, Harashima N, Yokoyama H, Srinivasan R, Linehan WM, Lerman MI, and Childs RW (2016). Detection of an Immunogenic HERV-E Envelope with Selective Expression in Clear Cell Kidney Cancer. Cancer Res 76, 2177–2185. 10.1158/0008-5472.CAN-15-3139. 26862115 PMC4873424

[40] Cherkasova E, Malinzak E, Rao S, Takahashi Y, Senchenko VN, Kudryavtseva AV, Nickerson ML, Merino M, Hong JA, Schrump DS, (2011). Inactivation of the von Hippel-Lindau tumor suppressor leads to selective expression of a human endogenous retrovirus in kidney cancer. Oncogene 30, 4697–4706. 10.1038/onc.2011.179. 21602888 PMC3161150

[41] Vargiu L, Rodriguez-Tome P, Sperber GO, Cadeddu M, Grandi N, Blikstad V, Tramontano E, and Blomberg J. (2016). Classification and characterization of human endogenous retroviruses; mosaic forms are common. Retrovirology 13, 7. 10.1186/s12977-015-0232-y. 26800882 PMC4724089

[42] Chen W, Hill H, Christie A, Kim MS, Holloman E, Pavia-Jimenez A, Homayoun F, Ma Y, Patel N, Yell P, (2016). Targeting renal cell carcinoma with a HIF-2 antagonist. Nature 539, 112–117. 10.1038/nature19796. 27595394 PMC5340502

[43] Cho H, Du X, Rizzi JP, Liberzon E, Chakraborty AA, Gao W, Carvo I, Signoretti S, Bruick RK, Josey JA, (2016). On-target efficacy of a HIF-2alpha antagonist in preclinical kidney cancer models. Nature 539, 107–111. 10.1038/nature19795. 27595393 PMC5499381

[44] Wallace EM, Rizzi JP, Han G, Wehn PM, Cao Z, Du X, Cheng T, Czerwinski RM, Dixon DD, Goggin BS, (2016). A Small-Molecule Antagonist of HIF2alpha Is Efficacious in Preclinical Models of Renal Cell Carcinoma. Cancer Res 76, 5491–5500. 10.1158/0008-5472.CAN-16-0473. 27635045

[45] Kondo K, Klco J, Nakamura E, Lechpammer M, and Kaelin WG Jr. (2002). Inhibition of HIF is necessary for tumor suppression by the von Hippel-Lindau protein. Cancer Cell 1, 237–246. 10.1016/s1535-6108(02)00043-0. 12086860

[46] Kondo K, Kim WY, Lechpammer M, and Kaelin WG Jr. (2003). Inhibition of HIF2alpha is sufficient to suppress pVHL-defective tumor growth. PLoS Biol 1, E83. 10.1371/journal.pbio.0000083. 14691554 PMC300692

[47] Pappalardi MB, Keenan K, Cockerill M, Kellner WA, Stowell A, Sherk C, Wong K, Pathuri S, Briand J, Steidel M, (2021). Discovery of a first-in-class reversible DNMT1-selective inhibitor with improved tolerability and efficacy in acute myeloid leukemia. Nat Cancer 2, 1002–1017. 34790902 PMC8594913

[48] Wiseman AK, Tiedemann RL, Fan H, Shen H, Madaj Z, McCabe MT, Pappalardi MB, and Jones PA (2022). Chromosome-specific retention of cancer-associated DNA hypermethylation following pharmacological inhibition of DNMT1. Commun Biol 5, 528. 10.1038/s42003-022-03509-3. 35654826 PMC9163065

[49] Shen C, Beroukhim R, Schumacher SE, Zhou J, Chang M, Signoretti S, and Kaelin WG Jr. (2011). Genetic and functional studies implicate HIF1alpha as a 14q kidney cancer suppressor gene. Cancer Discov 1, 222–235. 10.1158/2159-8290.CD-11-0098. 22037472 PMC3202343

[50] Griffin GK, Wu J, Iracheta-Vellve A, Patti JC, Hsu J, Davis T, Dele-Oni D, Du PP, Halawi AG, Ishizuka JJ, (2021). Epigenetic silencing by SETDB1 suppresses tumour intrinsic immunogenicity. Nature 595, 309–314. 10.1038/s41586-021-03520-4. 33953401 PMC9166167

[51] Zhang SM, Cai WL, Liu X, Thakral D, Luo J, Chan LH, McGeary MK, Song E, Blenman KRM, Micevic G, (2021). KDM5B promotes immune evasion by recruiting SETDB1 to silence retroelements. Nature 598, 682–687. 10.1038/s41586-021-03994-2. 34671158 PMC8555464

[52] Stransky LA, Vigeant SM, Huang B, West D, Denize T, Walton E, Signoretti S, and Kaelin WG Jr. (2022). Sensitivity of VHL mutant kidney cancers to HIF2 inhibitors does not require an intact p53 pathway. Proc Natl Acad Sci U S A 119, e2120403119. 10.1073/pnas.2120403119. PMC9168943 35357972

[53] Wang T, Birsoy K, Hughes NW, Krupczak KM, Post Y, Wei JJ, Lander ES, and Sabatini DM (2015). Identification and characterization of essential genes in the human genome. Science 350, 1096–1101. 10.1126/science.aac7041. 26472758 PMC4662922

[54] Kwak H, Fuda NJ, Core LJ, and Lis JT (2013). Precise maps of RNA polymerase reveal how promoters direct initiation and pausing. Science 339, 950–953. 10.1126/science.1229386. 23430654 PMC3974810

[55] Mahat DB, Kwak H, Booth GT, Jonkers IH, Danko CG, Patel RK, Waters CT, Munson K, Core LJ, and Lis JT (2016). Base-pair-resolution genome-wide mapping of active RNA polymerases using precision nuclear run-on (PRO-seq). Nat Protoc 11, 1455–1476. 10.1038/nprot.2016.086. 27442863 PMC5502525

[56] Reimer KA, Mimoso CA, Adelman K, and Neugebauer KM (2021). Co-transcriptional splicing regulates 3’ end cleavage during mammalian erythropoiesis. Mol Cell 81, 998–1012 e1017. 10.1016/j.molcel.2020.12.018. 33440169 PMC8038867

[57] Ingolia NT, Brar GA, Rouskin S, McGeachy AM, and Weissman JS (2012). The ribosome profiling strategy for monitoring translation in vivo by deep sequencing of ribosome-protected mRNA fragments. Nat Protoc 7, 1534–1550. 10.1038/nprot.2012.086. 22836135 PMC3535016

[58] Shalgi R, Hurt JA, Krykbaeva I, Taipale M, Lindquist S, and Burge CB (2013). Widespread regulation of translation by elongation pausing in heat shock. Mol Cell 49, 439–452. 10.1016/j.molcel.2012.11.028. 23290915 PMC3570722

[59] Au L, Hatipoglu E, Robert de Massy M, Litchfield K, Beattie G, Rowan A, Schnidrig D, Thompson R, Byrne F, Horswell S, (2021). Determinants of anti-PD-1 response and resistance in clear cell renal cell carcinoma. Cancer Cell 39, 1497–1518 e1411. 10.1016/j.ccell.2021.10.001. 34715028 PMC8599450

[60] Yu Z, Lv Y, Su C, Lu W, Zhang R, Li J, Guo B, Yan H, Liu D, Yang Z, (2023). Integrative Single-Cell Analysis Reveals Transcriptional and Epigenetic Regulatory Features of Clear Cell Renal Cell Carcinoma. Cancer Res 83, 700–719. 10.1158/0008-5472.CAN-22-2224. 36607615 PMC9978887

[61] Braun DA, Street K, Burke KP, Cookmeyer DL, Denize T, Pedersen CB, Gohil SH, Schindler N, Pomerance L, Hirsch L, (2021). Progressive immune dysfunction with advancing disease stage in renal cell carcinoma. Cancer Cell 39, 632–648 e638. 10.1016/j.ccell.2021.02.013. 33711273 PMC8138872

[62] Kula T, Dezfulian MH, Wang CI, Abdelfattah NS, Hartman ZC, Wucherpfennig KW, Lyerly HK, and Elledge SJ (2019). T-Scan: A Genome-wide Method for the Systematic Discovery of T Cell Epitopes. Cell 178, 1016–1028 e1013. 10.1016/j.cell.2019.07.009. 31398327 PMC6939866

[63] Kulkarni A, Mateus M, Thinnes CC, McCullagh JS, Schofield CJ, Taylor GP, and Bangham CRM (2017). Glucose Metabolism and Oxygen Availability Govern Reactivation of the Latent Human Retrovirus HTLV-1. Cell Chem Biol 24, 1377–1387 e1373. 10.1016/j.chembiol.2017.08.016. 28965728 PMC5696563

[64] Kewitz S, and Staege MS (2013). Expression and Regulation of the Endogenous Retrovirus 3 in Hodgkin’s Lymphoma Cells. Front Oncol 3, 179. 10.3389/fonc.2013.00179. 23847767 PMC3706881

[65] Brutting C, Narasimhan H, Hoffmann F, Kornhuber ME, Staege MS, and Emmer A. (2018). Investigation of Endogenous Retrovirus Sequences in the Neighborhood of Genes Up-regulated in a Neuroblastoma Model after Treatment with Hypoxia-Mimetic Cobalt Chloride. Front Microbiol 9, 287. 10.3389/fmicb.2018.00287. 29515560 PMC5826361

[66] Batie M, Frost J, Frost M, Wilson JW, Schofield P, and Rocha S. (2019). Hypoxia induces rapid changes to histone methylation and reprograms chromatin. Science 363, 1222–1226. 10.1126/science.aau5870. 30872526

[67] Chakraborty AA, Laukka T, Myllykoski M, Ringel AE, Booker MA, Tolstorukov MY, Meng YJ, Meier SR, Jennings RB, Creech AL, (2019). Histone demethylase KDM6A directly senses oxygen to control chromatin and cell fate. Science 363, 1217–1222. 10.1126/science.aaw1026. 30872525 PMC7336390

[68] Bi K, He MX, Bakouny Z, Kanodia A, Napolitano S, Wu J, Grimaldi G, Braun DA, Cuoco MS, Mayorga A, (2021). Tumor and immune reprogramming during immunotherapy in advanced renal cell carcinoma. Cancer Cell 39, 649–661 e645. 10.1016/j.ccell.2021.02.015. 33711272 PMC8115394

[69] Miao D, Margolis CA, Gao W, Voss MH, Li W, Martini DJ, Norton C, Bosse D, Wankowicz SM, Cullen D, (2018). Genomic correlates of response to immune checkpoint therapies in clear cell renal cell carcinoma. Science 359, 801–806. 10.1126/science.aan5951. 29301960 PMC6035749

[70] Nargund AM, Pham CG, Dong Y, Wang PI, Osmangeyoglu HU, Xie Y, Aras O, Han S, Oyama T, Takeda S, (2017). The SWI/SNF Protein PBRM1 Restrains VHL-Loss-Driven Clear Cell Renal Cell Carcinoma. Cell Rep 18, 2893–2906. 10.1016/j.celrep.2017.02.074. 28329682 PMC5431084

[71] Gao W, Li W, Xiao T, Liu XS, and Kaelin WG Jr. (2017). Inactivation of the PBRM1 tumor suppressor gene amplifies the HIF-response in VHL−/− clear cell renal carcinoma. Proc Natl Acad Sci U S A 114, 1027–1032. 10.1073/pnas.1619726114. 28082722 PMC5293026

[72] Zhou M, Leung JY, Gessner KH, Hepperla AJ, Simon JM, Davis IJ, and Kim WY (2022). PBRM1 Inactivation Promotes Upregulation of Human Endogenous Retroviruses in a HIF-Dependent Manner. Cancer Immunol Res 10, 285–290. 10.1158/2326-6066.CIR-21-0480. 35013001 PMC8898299

[73] Ehrlich M. (2009). DNA hypomethylation in cancer cells. Epigenomics 1, 239–259. 10.2217/epi.09.33. 20495664 PMC2873040

[74] Cao JZ, Liu H, Wickrema A, and Godley LA (2020). HIF-1 directly induces TET3 expression to enhance 5-hmC density and induce erythroid gene expression in hypoxia. Blood Adv 4, 3053–3062. 10.1182/bloodadvances.2020001535. 32634239 PMC7362358

[75] Mariani CJ, Vasanthakumar A, Madzo J, Yesilkanal A, Bhagat T, Yu Y, Bhattacharyya S, Wenger RH, Cohn SL, Nanduri J, (2014). TET1-mediated hydroxymethylation facilitates hypoxic gene induction in neuroblastoma. Cell Rep 7, 1343–1352. 10.1016/j.celrep.2014.04.040. 24835990 PMC4516227

[76] Kindrick JD, and Mole DR (2020). Hypoxic Regulation of Gene Transcription and Chromatin: Cause and Effect. Int J Mol Sci 21. 10.3390/ijms21218320. PMC7664190 33171917

[77] Rakoff-Nahoum S, Kuebler PJ, Heymann JJ, M ES, Ortiz GM, G SO, Barbour, Lenz J, Steinfeld AD, and Nixon DF. (2006). Detection of T lymphocytes specific for human endogenous retrovirus K (HERV-K) in patients with seminoma. AIDS Res Hum Retroviruses 22, 52–56. 10.1089/aid.2006.22.52. 16438646

[78] Schiavetti F, Thonnard J, Colau D, Boon T, and Coulie PG (2002). A human endogenous retroviral sequence encoding an antigen recognized on melanoma by cytolytic T lymphocytes. Cancer Res 62, 5510–5516. 12359761

[79] Mullins CS, and Linnebacher M. (2012). Endogenous retrovirus sequences as a novel class of tumor-specific antigens: an example of HERV-H env encoding strong CTL epitopes. Cancer Immunol Immunother 61, 1093–1100. 10.1007/s00262-011-1183-3. 22187063 PMC11029769

[80] Laumont CM, Vincent K, Hesnard L, Audemard E, Bonneil E, Laverdure JP, Gendron P, Courcelles M, Hardy MP, Cote C, (2018). Noncoding regions are the main source of targetable tumor-specific antigens. Sci Transl Med 10. 10.1126/scitranslmed.aau5516. 30518613

[81] Kobayashi S, Tokita S, Moniwa K, Kitahara K, Iuchi H, Matsuo K, Kakizaki H, Kanaseki T, and Torigoe T. (2023). Proteogenomic identification of an immunogenic antigen derived from human endogenous retrovirus in renal cell carcinoma. JCI Insight 8. 10.1172/jci.insight.167712. PMC10543709 37606040

[82] Roulois D, Loo Yau H, Singhania R, Wang Y, Danesh A, Shen SY, Han H, Liang G, Jones PA, Pugh TJ, (2015). DNA-Demethylating Agents Target Colorectal Cancer Cells by Inducing Viral Mimicry by Endogenous Transcripts. Cell 162, 961–973. 10.1016/j.cell.2015.07.056. 26317465 PMC4843502

[83] Chiappinelli KB, Strissel PL, Desrichard A, Li H, Henke C, Akman B, Hein A, Rote NS, Cope LM, Snyder A, (2015). Inhibiting DNA Methylation Causes an Interferon Response in Cancer via dsRNA Including Endogenous Retroviruses. Cell 162, 974–986. 10.1016/j.cell.2015.07.011. 26317466 PMC4556003

[84] D’Anna F, Van Dyck L, Xiong J, Zhao H, Berrens RV, Qian J, Bieniasz-Krzywiec P, Chandra V, Schoonjans L, Matthews J, (2020). DNA methylation repels binding of hypoxia-inducible transcription factors to maintain tumor immunotolerance. Genome Biol 21, 182. 10.1186/s13059-020-02087-z. 32718321 PMC7384226

[85] Dranoff G, Jaffee E, Lazenby A, Golumbek P, Levitsky H, Brose K, Jackson V, Hamada H, Pardoll D, and Mulligan RC (1993). Vaccination with irradiated tumor cells engineered to secrete murine granulocyte-macrophage colony-stimulating factor stimulates potent, specific, and long-lasting anti-tumor immunity. Proc Natl Acad Sci U S A 90, 3539–3543. 10.1073/pnas.90.8.3539. 8097319 PMC46336

[86] Ng KW, Boumelha J, Enfield KSS, Almagro J, Cha H, Pich O, Karasaki T, Moore DA, Salgado R, Sivakumar M, (2023). Antibodies against endogenous retroviruses promote lung cancer immunotherapy. Nature 616, 563–573. 10.1038/s41586-023-05771-9. 37046094 PMC10115647

[87] Keith B, Johnson RS, and Simon MC (2011). HIF1alpha and HIF2alpha: sibling rivalry in hypoxic tumour growth and progression. Nat Rev Cancer 12, 9–22. 10.1038/nrc3183. 22169972 PMC3401912

[88] Siebenthall KT, Miller CP, Vierstra JD, Mathieu J, Tretiakova M, Reynolds A, Sandstrom R, Rynes E, Haugen E, Johnson A, (2019). Integrated epigenomic profiling reveals endogenous retrovirus reactivation in renal cell carcinoma. EBioMedicine 41, 427–442. 10.1016/j.ebiom.2019.01.063. 30827930 PMC6441874

[89] Klebanoff CA, Chandran SS, Baker BM, Quezada SA, and Ribas A. (2023). T cell receptor therapeutics: immunological targeting of the intracellular cancer proteome. Nat Rev Drug Discov 22, 996–1017. 10.1038/s41573-023-00809-z. 37891435 PMC10947610

[90] Malviya M, Aretz ZEH, Molvi Z, Lee J, Pierre S, Wallisch P, Dao T, and Scheinberg DA (2023). Challenges and solutions for therapeutic TCR-based agents. Immunol Rev 320, 58–82. 10.1111/imr.13233. 37455333 PMC11141734

[91] Akitsu A, Kobayashi E, Feng Y, Stephens HM, Brazin KN, Masi DJ, Kirkpatrick EH, Mallis RJ, Duke-Cohan JS, Booker MA, (2024). Parsing digital or analog TCR performance through piconewton forces. Sci Adv 10, eado4313. 10.1126/sciadv.ado4313. PMC11323890 39141734

[92] Wang Z, Lyu Z, Pan L, Zeng G, and Randhawa P. (2019). Defining housekeeping genes suitable for RNA-seq analysis of the human allograft kidney biopsy tissue. BMC Med Genomics 12, 86. 10.1186/s12920-019-0538-z. 31208411 PMC6580566

[93] Ferretti AP, Kula T, Wang Y, Nguyen DMV, Weinheimer A, Dunlap GS, Xu Q, Nabilsi N, Perullo CR, Cristofaro AW, (2020). Unbiased Screens Show CD8(+) T Cells of COVID-19 Patients Recognize Shared Epitopes in SARS-CoV-2 that Largely Reside outside the Spike Protein. Immunity 53, 1095–1107 e1093. 10.1016/j.immuni.2020.10.006. 33128877 PMC7574860

[94] Yu Z, Lv Y, Su C, Lu W, Zhang R, Li J, Guo B, Yan H, Liu D, Yang Z, (2023). Integrative Single-Cell Analysis Reveals Transcriptional and Epigenetic Regulatory Features of Clear Cell Renal Cell Carcinoma. Cancer Research 83, 700–719. 10.1158/0008-5472.CAN-22-2224. 36607615 PMC9978887

[95] Wakimoto H, Tanaka S, Curry WT, Loebel F, Zhao D, Tateishi K, Chen J, Klofas LK, Lelic N, Kim JC, (2014). Targetable signaling pathway mutations are associated with malignant phenotype in IDH-mutant gliomas. Clin Cancer Res 20, 2898–2909. 10.1158/1078-0432.CCR-13-3052. 24714777 PMC4070445

[96] Krishna C, DiNatale RG, Kuo F, Srivastava RM, Vuong L, Chowell D, Gupta S, Vanderbilt C, Purohit TA, Liu M, (2021). Single-cell sequencing links multiregional immune landscapes and tissue-resident T cells in ccRCC to tumor topology and therapy efficacy. Cancer Cell 39, 662–677 e666. 10.1016/j.ccell.2021.03.007. 33861994 PMC8268947

[97] Nicholson HE, Tariq Z, Housden BE, Jennings RB, Stransky LA, Perrimon N, Signoretti S, and Kaelin WG Jr. (2019). HIF-independent synthetic lethality between CDK4/6 inhibition and VHL loss across species. Sci Signal 12. 10.1126/scisignal.aay0482. PMC6913182 31575731

[98] Langmead B, and Salzberg SL (2012). Fast gapped-read alignment with Bowtie 2. Nat Methods 9, 357–359. 10.1038/nmeth.1923. 22388286 PMC3322381

[99] Anders S, Pyl PT, and Huber W. (2015). HTSeq--a Python framework to work with high-throughput sequencing data. Bioinformatics 31, 166–169. 10.1093/bioinformatics/btu638. 25260700 PMC4287950

[100] Roth TL, Li PJ, Blaeschke F, Nies JF, Apathy R, Mowery C, Yu R, Nguyen MLT, Lee Y, Truong A, (2020). Pooled Knockin Targeting for Genome Engineering of Cellular Immunotherapies. Cell 181, 728–744 e721. 10.1016/j.cell.2020.03.039. 32302591 PMC7219528

[101] Schodel J, Oikonomopoulos S, Ragoussis J, Pugh CW, Ratcliffe PJ, and Mole DR (2011). High-resolution genome-wide mapping of HIF-binding sites by ChIP-seq. Blood 117, e207–217. 10.1182/blood-2010-10-314427. 21447827 PMC3374576

[102] Qin Q, Mei S, Wu Q, Sun H, Li L, Taing L, Chen S, Li F, Liu T, Zang C, (2016). ChiLin: a comprehensive ChIP-seq and DNase-seq quality control and analysis pipeline. BMC Bioinformatics 17, 404. 10.1186/s12859-016-1274-4. 27716038 PMC5048594

[103] Qiu X, Feit AS, Feiglin A, Xie Y, Kesten N, Taing L, Perkins J, Gu S, Li Y, Cejas P, (2021). CoBRA: Containerized Bioinformatics Workflow for Reproducible ChIP/ATAC-seq Analysis. Genomics Proteomics Bioinformatics 19, 652–661. 10.1016/j.gpb.2020.11.007. 34284136 PMC9039557

[104] Li H, and Durbin R. (2009). Fast and accurate short read alignment with Burrows-Wheeler transform. Bioinformatics 25, 1754–1760. 10.1093/bioinformatics/btp324. 19451168 PMC2705234

[105] Zhang Y, Liu T, Meyer CA, Eeckhoute J, Johnson DS, Bernstein BE, Nusbaum C, Myers RM, Brown M, Li W, and Liu XS (2008). Model-based analysis of ChIP-Seq (MACS). Genome Biol 9, R137. 10.1186/gb-2008-9-9-r137. 18798982 PMC2592715

[106] Neph S, Kuehn MS, Reynolds AP, Haugen E, Thurman RE, Johnson AK, Rynes E, Maurano MT, Vierstra J, Thomas S, (2012). BEDOPS: high-performance genomic feature operations. Bioinformatics 28, 1919–1920. 10.1093/bioinformatics/bts277. 22576172 PMC3389768

[107] Quinlan AR, and Hall IM (2010). BEDTools: a flexible suite of utilities for comparing genomic features. Bioinformatics 26, 841–842. 10.1093/bioinformatics/btq033. 20110278 PMC2832824

[108] Weingarten-Gabbay S, Pearlman LR, Chen DY, Klaeger S, Taylor HB, Welch NL, Keskin DB, Carr SA, Abelin JG, Saeed M, and Sabeti PC (2022). HLA-I immunopeptidome profiling of human cells infected with high-containment enveloped viruses. STAR Protoc 3, 101910. 10.1016/j.xpro.2022.101910. 36595954 PMC9731565

[109] Keskin DB, Reinhold B, Lee SY, Zhang G, Lank S, O’Connor DH, Berkowitz RS, Brusic V, Kim SJ, and Reinherz EL (2011). Direct identification of an HPV-16 tumor antigen from cervical cancer biopsy specimens. Front Immunol 2, 75. 10.3389/fimmu.2011.00075. 22566864 PMC3342284

[110] Klaeger S, Apffel A, Clauser KR, Sarkizova S, Oliveira G, Rachimi S, Le PM, Tarren A, Chea V, Abelin JG, (2021). Optimized Liquid and Gas Phase Fractionation Increases HLA-Peptidome Coverage for Primary Cell and Tissue Samples. Mol Cell Proteomics 20, 100133. 10.1016/j.mcpro.2021.100133. PMC8724927 34391888

[111] Ruggles KV, Tang Z, Wang X, Grover H, Askenazi M, Teubl J, Cao S, McLellan MD, Clauser KR, Tabb DL, (2016). An Analysis of the Sensitivity of Proteogenomic Mapping of Somatic Mutations and Novel Splicing Events in Cancer. Mol Cell Proteomics 15, 1060–1071. 10.1074/mcp.M115.056226. 26631509 PMC4813688

[112] Nakagawa S, and Takahashi MU (2016). gEVE: a genome-based endogenous viral element database provides comprehensive viral protein-coding sequences in mammalian genomes. Database (Oxford) 2016. 10.1093/database/baw087. PMC4885607 27242033

[113] Wilhelm M, Zolg DP, Graber M, Gessulat S, Schmidt T, Schnatbaum K, Schwencke-Westphal C, Seifert P, de Andrade Kratzig N, Zerweck J, (2021). Deep learning boosts sensitivity of mass spectrometry-based immunopeptidomics. Nat Commun 12, 3346. 10.1038/s41467-021-23713-9. 34099720 PMC8184761

[114] Bouwmeester R, Gabriels R, Hulstaert N, Martens L, and Degroeve S. (2021). DeepLC can predict retention times for peptides that carry as-yet unseen modifications. Nat Methods 18, 1363–1369. 10.1038/s41592-021-01301-5. 34711972

[115] Sarkizova S, Klaeger S, Le PM, Li LW, Oliveira G, Keshishian H, Hartigan CR, Zhang W, Braun DA, Ligon KL, (2020). A large peptidome dataset improves HLA class I epitope prediction across most of the human population. Nat Biotechnol 38, 199–209. 10.1038/s41587-019-0322-9. 31844290 PMC7008090

[116] Li H. (2011). Tabix: fast retrieval of sequence features from generic TAB-delimited files. Bioinformatics 27, 718–719. 10.1093/bioinformatics/btq671. 21208982 PMC3042176

[117] Stuart T, Srivastava A, Madad S, Lareau CA, and Satija R. (2021). Single-cell chromatin state analysis with Signac. Nature Methods 18, 1333–1341. 10.1038/s41592-021-01282-5. 34725479 PMC9255697

[118] Hao Y, Stuart T, Kowalski MH, Choudhary S, Hoffman P, Hartman A, Srivastava A, Molla G, Madad S, Fernandez-Granda C, and Satija R. (2024). Dictionary learning for integrative, multimodal and scalable single-cell analysis. Nature Biotechnology 42, 293–304. 10.1038/s41587-023-01767-y. PMC10928517 37231261

[119] Germain PL, Lun A, Garcia Meixide C, Macnair W, and Robinson MD 10.12688/f1000research.73600.1 PMC9204188 35814628

[120] Korsunsky I, Millard N, Fan J, Slowikowski K, Zhang F, Wei K, Baglaenko Y, Brenner M, Loh P. r., and Raychaudhuri S. (2019). Fast, sensitive and accurate integration of single-cell data with Harmony. Nature Methods 16, 1289–1296. 10.1038/s41592-019-0619-0. 31740819 PMC6884693

[121] Young MD, Mitchell TJ, Vieira Braga FA, Tran MGB, Stewart BJ, Ferdinand JR, Collord G, Botting RA, Popescu D-M, Loudon KW, (2018). Single-cell transcriptomes from human kidneys reveal the cellular identity of renal tumors. Science 361, 594–599. 10.1126/science.aat1699. 30093597 PMC6104812

[122] Kourtis N, Wang Q, Wang B, Oswald E, Adler C, Cherravuru S, Malahias E, Zhang L, Golubov J, Wei Q, (2022). A single-cell map of dynamic chromatin landscapes of immune cells in renal cell carcinoma. Nature Cancer 3, 885–898. 10.1038/s43018-022-00391-0. 35668194 PMC9325682

[123] Müller AM, Hermanns MI, Skrzynski C, Nesslinger M, Müller K-M, and Kirkpatrick CJ (2002). Expression of the Endothelial Markers PECAM-1, vWf, and CD34 in Vivo and in Vitro. Experimental and Molecular Pathology 72, 221–229. 10.1006/exmp.2002.2424. 12009786

[124] Tombor LS, John D, Glaser SF, Luxán G, Forte E, Furtado M, Rosenthal N, Baumgarten N, Schulz MH, Wittig J, (2021). Single cell sequencing reveals endothelial plasticity with transient mesenchymal activation after myocardial infarction. Nature Communications 12, 681. 10.1038/s41467-021-20905-1. PMC7846794 33514719

[125] Nikolic A, Singhal D, Ellestad K, Johnston M, Shen Y, Gillmor A, Morrissy S, Cairncross JG, Jones S, Lupien M, Copy-scAT: Deconvoluting single-cell chromatin accessibility of genetic subclones in cancer. Science Advances 7, eabg6045. 10.1126/sciadv.abg6045. PMC8514091 34644115

[126] Lawrence M, Huber W, Pagès H, Aboyoun P, Carlson M, Gentleman R, Morgan MT, and Carey VJ (2013). Software for Computing and Annotating Genomic Ranges. PLOS Computational Biology 9, e1003118. 10.1371/journal.pcbi.1003118. PMC3738458 23950696

[127] Wang Q, Li M, Wu T, Zhan L, Li L, Chen M, Xie W, Xie Z, Hu E, Xu S, and Yu G. (2022). Exploring Epigenomic Datasets by ChIPseeker. Curr Protoc 2, e585. 10.1002/cpz1.585. 36286622

[128] Danecek P, Bonfield JK, Liddle J, Marshall J, Ohan V, Pollard MO, Whitwham A, Keane T, McCarthy SA, Davies RM, and Li H. (2021). Twelve years of SAMtools and BCFtools. GigaScience 10, giab008. 10.1093/gigascience/giab008. PMC7931819 33590861

[129] He J, Babarinde IA, Sun L, Xu S, Chen R, Shi J, Wei Y, Li Y, Ma G, Zhuang Q, (2021). Identifying transposable element expression dynamics and heterogeneity during development at the single-cell level with a processing pipeline scTE. Nature Communications 12, 1456. 10.1038/s41467-021-21808-x. PMC7935913 33674594

[130] Keskin DB, Anandappa AJ, Sun J, Tirosh I, Mathewson ND, Li S, Oliveira G, Giobbie-Hurder A, Felt K, Gjini E, (2019). Neoantigen vaccine generates intratumoral T cell responses in phase Ib glioblastoma trial. Nature 565, 234–239. 10.1038/s41586-018-0792-9. 30568305 PMC6546179

[131] Lee PC, Klaeger S, Le PM, Korthauer K, Cheng J, Ananthapadmanabhan V, Frost TC, Stevens JD, Wong AY, Iorgulescu JB, (2022). Reversal of viral and epigenetic HLA class I repression in Merkel cell carcinoma. J Clin Invest 132. 10.1172/JCI151666. PMC9246387 35775490

[132] Kan-Mitchell J, Bisikirska B, Wong-Staal F, Schaubert KL, Bajcz M, and Bereta M. (2004). The HIV-1 HLA-A2-SLYNTVATL is a help-independent CTL epitope. J Immunol 172, 5249–5261. 10.4049/jimmunol.172.9.5249. 15100263

[133] Luoma AM, Suo S, Wang Y, Gunasti L, Porter CBM, Nabilsi N, Tadros J, Ferretti AP, Liao S, Gurer C, (2022). Tissue-resident memory and circulating T cells are early responders to pre-surgical cancer immunotherapy. Cell 185, 2918–2935 e2929. 10.1016/j.cell.2022.06.018. 35803260 PMC9508682

