# Supplementary figures and images for: HIF Regulates Multiple Translated Endogenous Retroviruses: Implications for Cancer Immunotherapy
Source: Cell. Author manuscript; Available in PMC 2025 Apr 11. (PMC11988688; doi:10.1016/j.cell.2025.01.046)

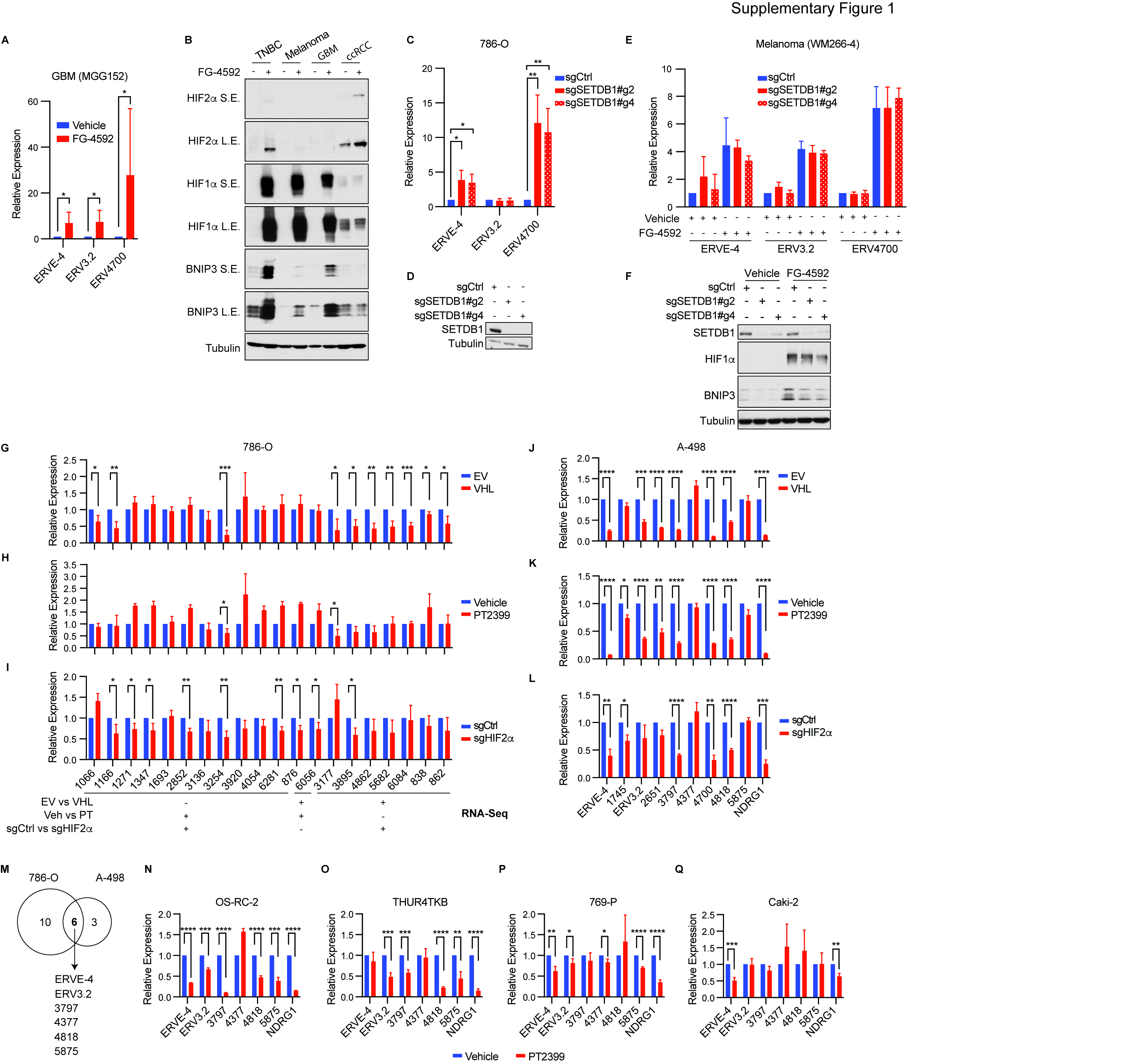

Supplement: 1 [file NIHMS2060984-supplement-1.tif]

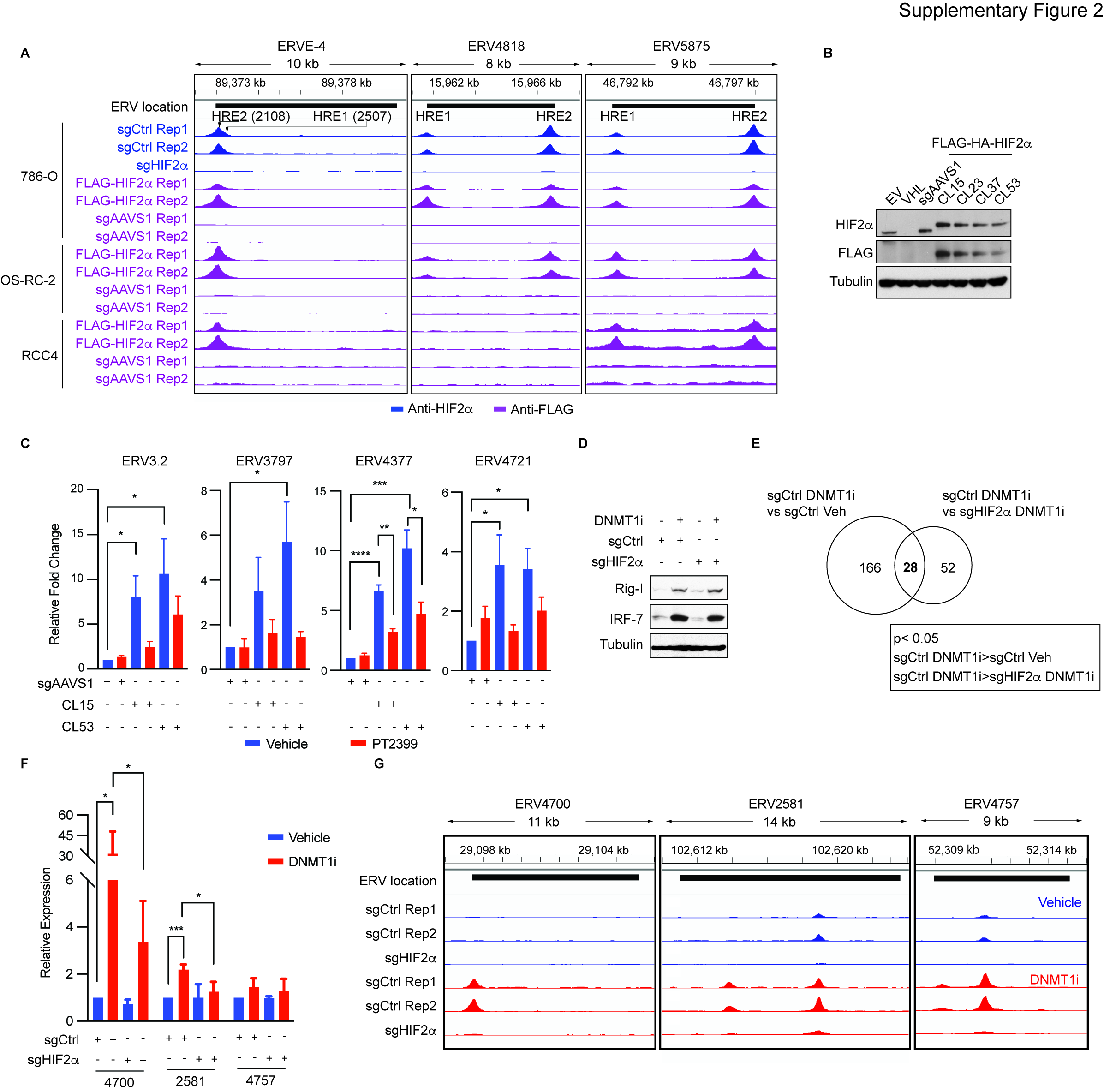

Supplement: 2 — Figure S2. Identification of HIF2 Binding Sites Near ERVs, Related to Figure 3 (A) Anti-HIF2α ChIP-seq tracks (blue) in 786-O cells that underwent CRISPR-based gene editing with a HIF2α sgRNA or a control sgRNA (sgCtrl) and Anti-FLAG ChIP-seq tracks (purple) in 786-O, OS-RC-2 and RCC4 cells in which a FLAG-HA epitope tag coding sequence was inserted at the 5’ end of the endogenous HIF2α open reading frame by CRISPR-HDR (FLAG-HIF2α). Parental cells infected with a control guide RNA (sgAAVS1) were included as controls. All the experiments were conducted in duplicates except for Anti-HIF2α ChIP-seq in 786-O cells with a HIF2α sgRNA, which was conducted only once. Rep 1 corresponds to the data in Figure 3D. (B) Anti-FLAG immunoblot analysis for 4 different clones of 786-O cells in which a FLAG-HA epitope tag coding sequence was inserted at the 5’ end of the endogenous HIF2α open reading frame by CRISPR-HDR compared to parental cells that were infected with a control guide RNA (sgAAVS1). 786-O cells stably infected to express pVHL (VHL) or with the empty vector (EV) were included as additional controls. (C) Anti-FLAG ChIP-qPCR assays using two different clones (CL15 and CL53) of 786-O cells as shown in (B). Cells were treated with 2 μM PT2399 or vehicle for 72 hours prior to ChIP-qPCR with primers designed to interrogate HREs for the indicated ERVs. Data were normalized to the untreated sgAAVS1 cells. (N=4) (D and E) Immunoblot analysis (D) and Venn Diagram of DNMT1i-responsive ERVs based on RNA-seq (E) of 786-O cells that underwent CRISPR-based gene editing with a HIF2α sgRNA or control sgRNA (sgCtrl) and then treated with 0.5 μM GSK3685032 (DNMT1i) or vehicle (Veh) for 9 days. Note that in (E) 28 ERVs were derepressed by treatment with 0.5 μM GSK3685032 (DNMT1i) for 9 days compared to vehicle-treated cells in a HIF2-dependent manner. (F and G) RT-qPCR validation (F) and anti-HIF2α ChIP-seq (G) of 3 ERVs that were 1) one of the 28 as shown in (E) and 2) located within 1 [file NIHMS2060984-supplement-2.tif]

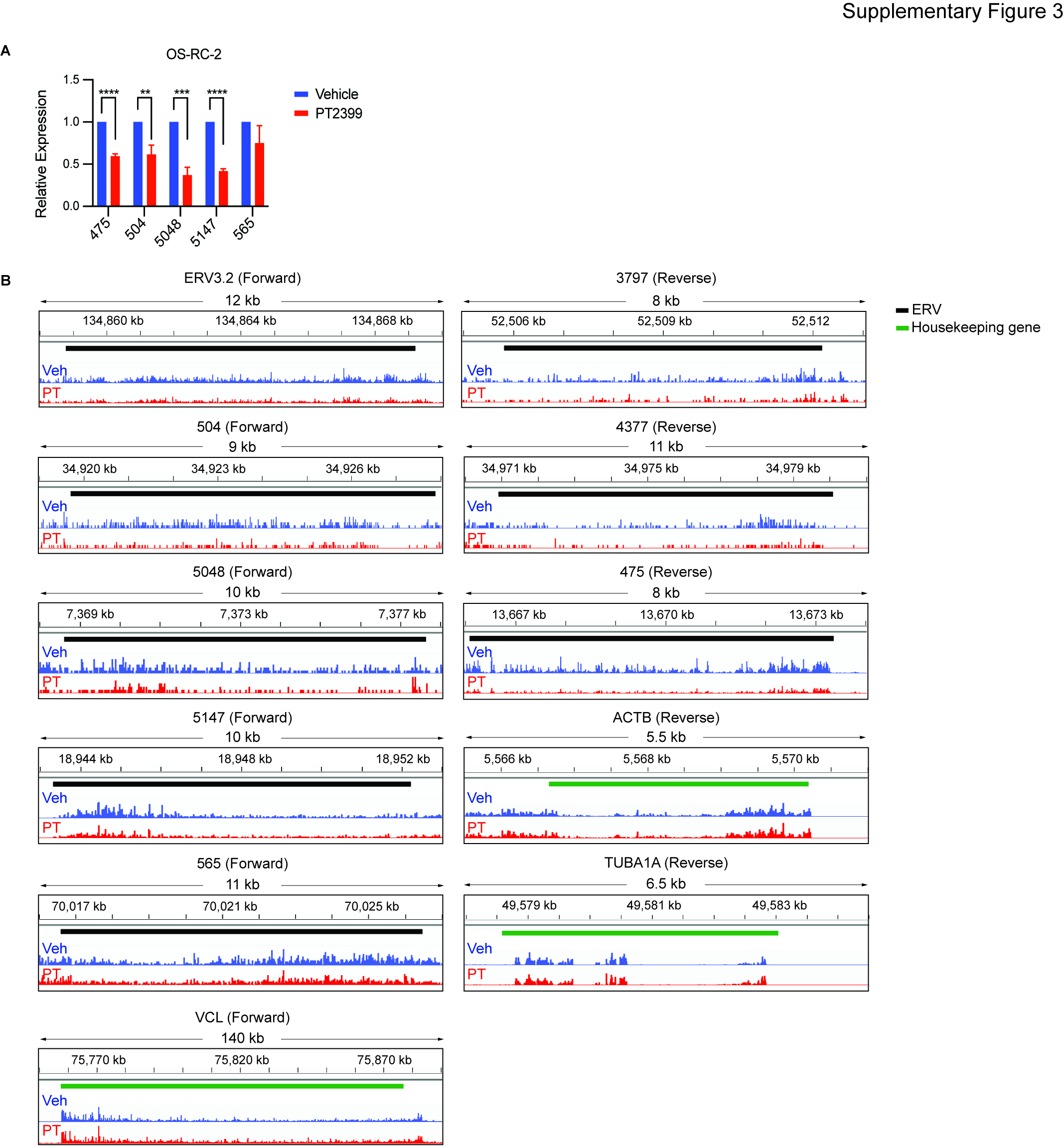

Supplement: 3 — Figure S3. ERVs that are Direct HIF2 Transcriptional Targets in OS-RC-2 Cells, Related to Figure 3 (A) ERV levels in OS-RC-2 cells treated with either 2 μM PT2399 or vehicle for 72 hours. ERV RNA levels were normalized to ACTB RNA levels and then to the vehicle condition. (N=3) (B) Representative nascent RNA tracks in OS-RC-2 cells that were treated with 2 μM PT2399 (PT) or vehicle (Veh) for 2 hours. All 8 listed ERVs scored as HIF2-responsive in the PRO-seq analysis. The housekeeping genes ACTB, VCL and TUBA1A were included as controls. [file NIHMS2060984-supplement-3.tif]

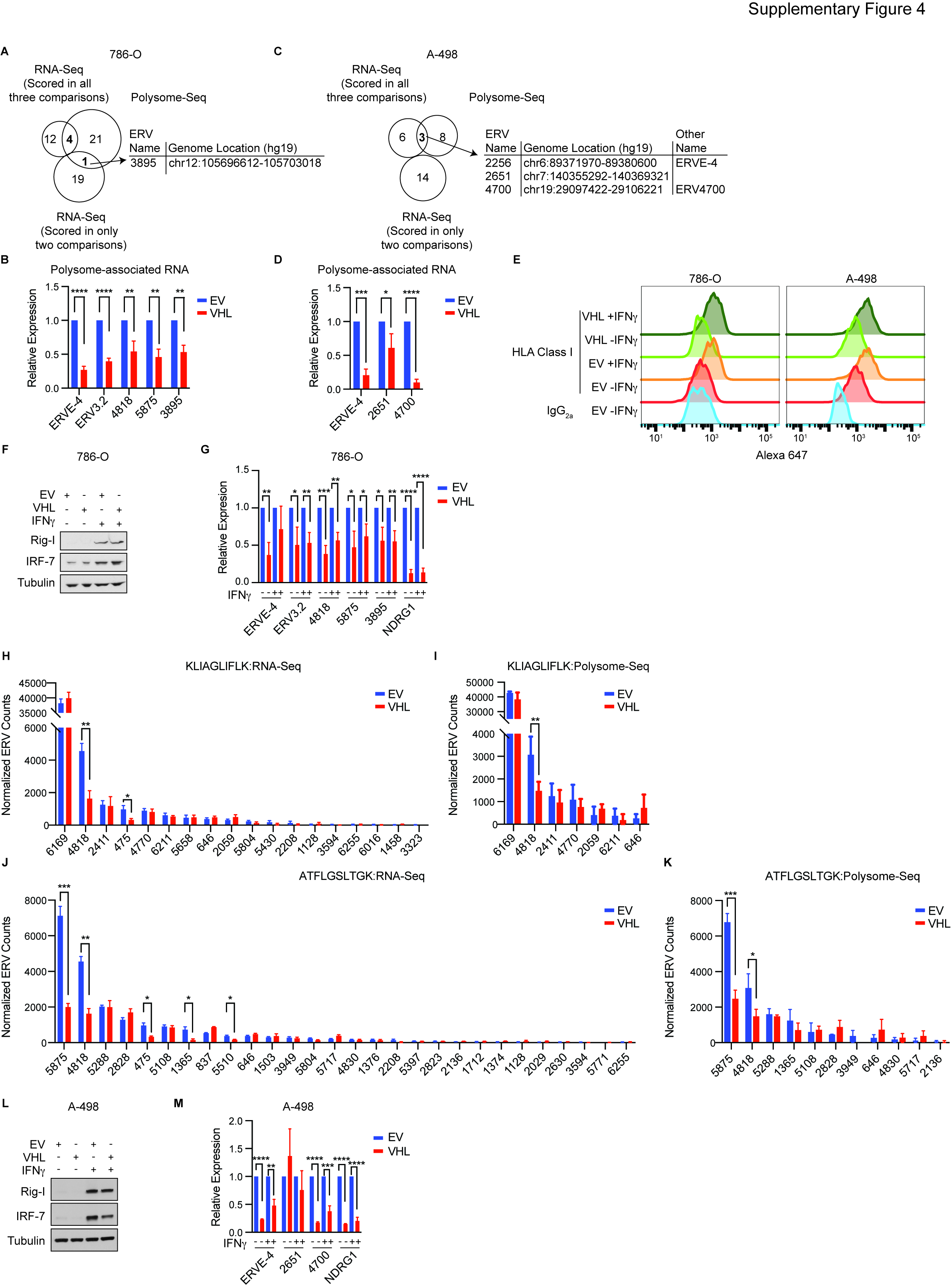

Supplement: 4 [file NIHMS2060984-supplement-4.tif]

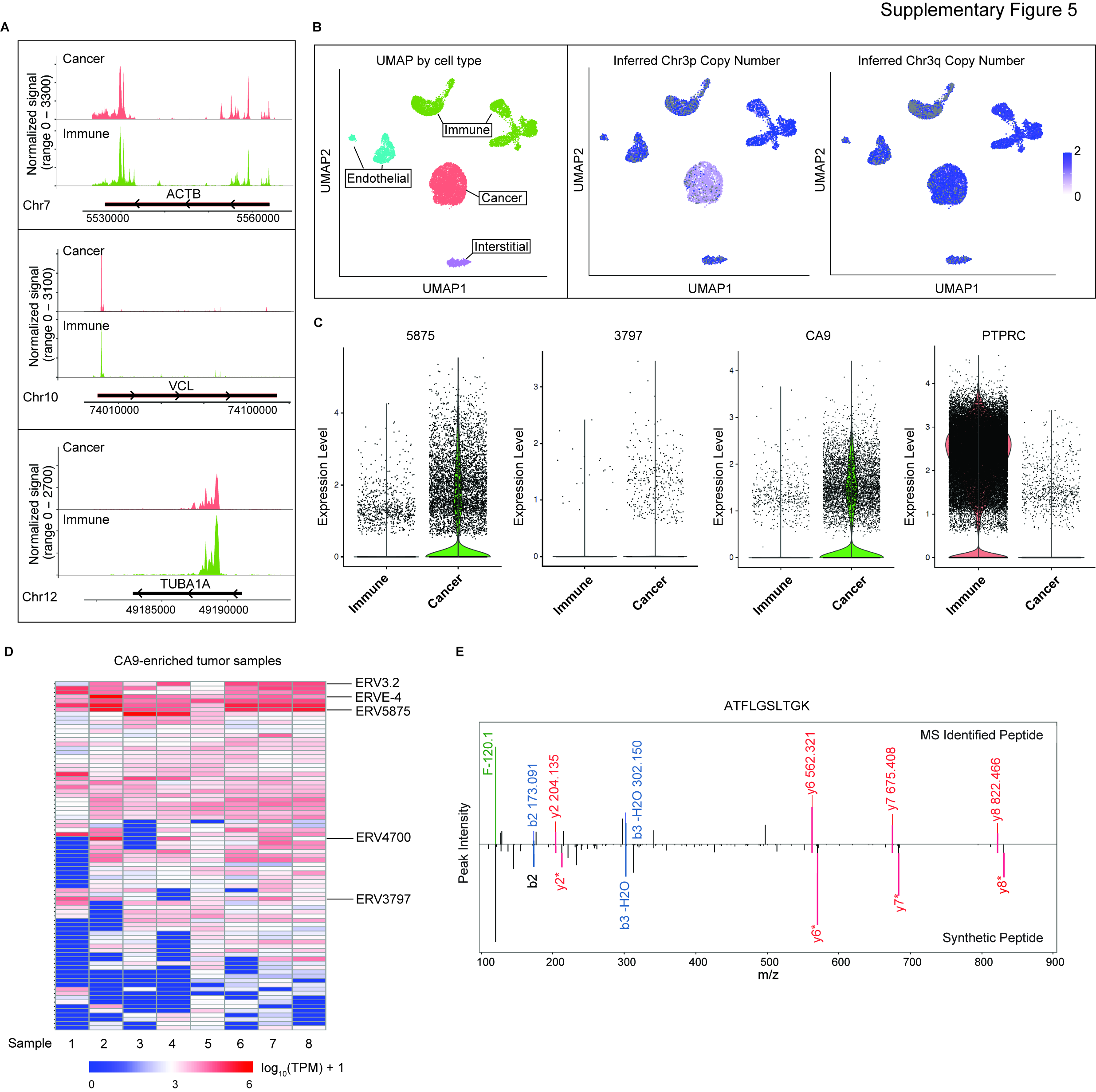

Supplement: 5 — Figure S5. Human HIF2-responsive ERVs are Largely Derived from Cancer Cells, related to Figure 5; MS/MS spectra for ERV-derived ATFLGSLTGK peptide, related to Figure 6 (A) Coverage plot showing scATAC-seq tracks at the housekeeping genes ACTB, VCL and TUBA1A ± 3 kb. (B) UMAP for scATAC-seq data from Yu et al60. In left panel, cell type inferred based on chromatin accessibility at promoters of cell type specific genes. In the middle and right panels, chromosome 3p and 3q copy number inferred using CopyscAT. (C) Violin plots depicting RNA levels for the indicated ERVs and genes in Cancer or Immune cells based on scRNA-seq data from Yu et al60. (D) Heatmap showing the abundance of the 81 HIF2-responsive ERVs based on Log10(TPM, transcripts per million)+1 of the RNA-seq data from CA9-enriched tumor samples derived from patients enrolled in the NCT02950766 clinical trial. Samples are labeled as “1–8”, the detailed information for each tumor sample is listed in Table S5. (E) MS/MS spectra comparing HLA-bound ERV-derived peptide ATFLGSLTGK from tumor immunopeptidome of RCC patient 110 (1 out of 3 patient with alleles HLA-A*03:01 or -A*11:01) (upper) and the corresponding synthetic peptide bearing a heavy lysine (Lys8, lower). The tumor spectrum has a Spectrum Mill sequence coverage metric, backbone cleavage score (BCS) of 4 that is below the automated threshold of 5 used for confident identification. However, comparison to the synthetic peptide is a clear match, comparable to the match shown in Figure 4F for the peptide detected in the immunopeptidome of the 786-O cell line. [file NIHMS2060984-supplement-5.tif]

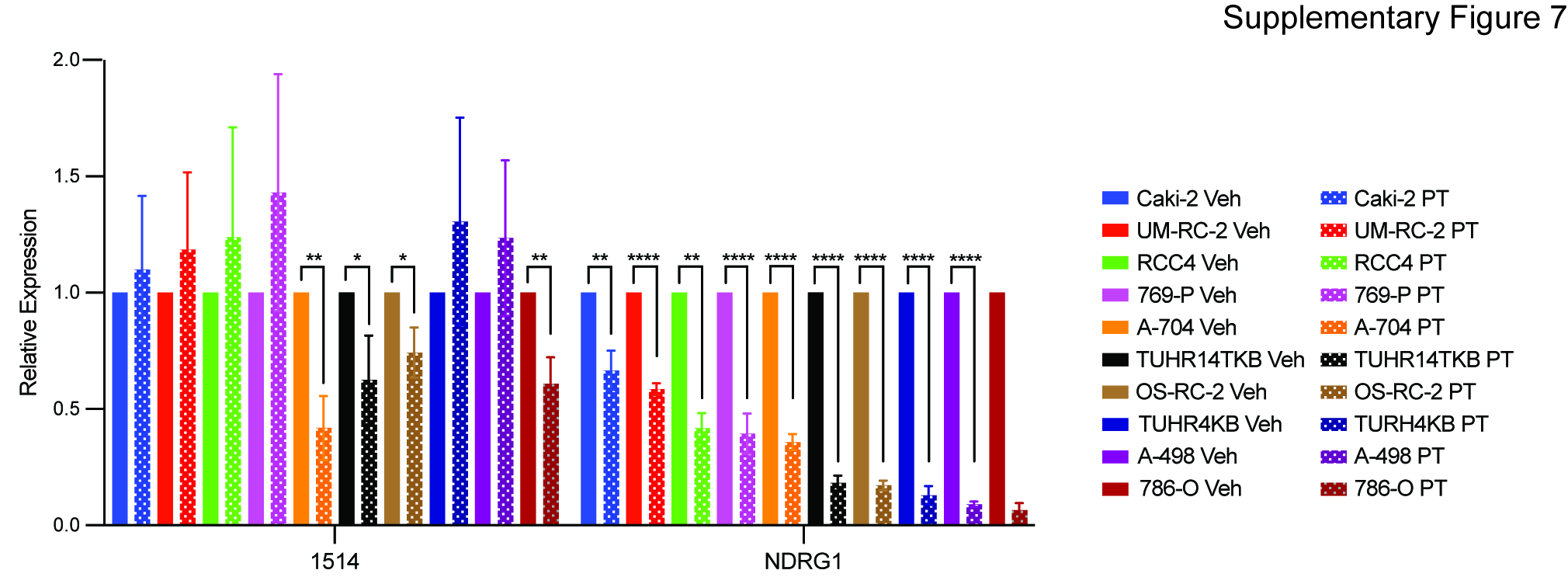

Supplement: 6 — Figure S7. ERV1514 is HIF2-responsive in 4/10 ccRCC cell lines, related to Figure 7 ERV1514 RNA level was examined by RT-qPCR in 10 ccRCC cell lines. The cell lines were treated with either 2 μM PT2399 or vehicle for 72 hours. NDRG1 mRNA levels were included as controls. ERV RNA levels were normalized to ACTB RNA levels and then to the vehicle condition. For each cell line N=2–4. [file NIHMS2060984-supplement-6.tif]

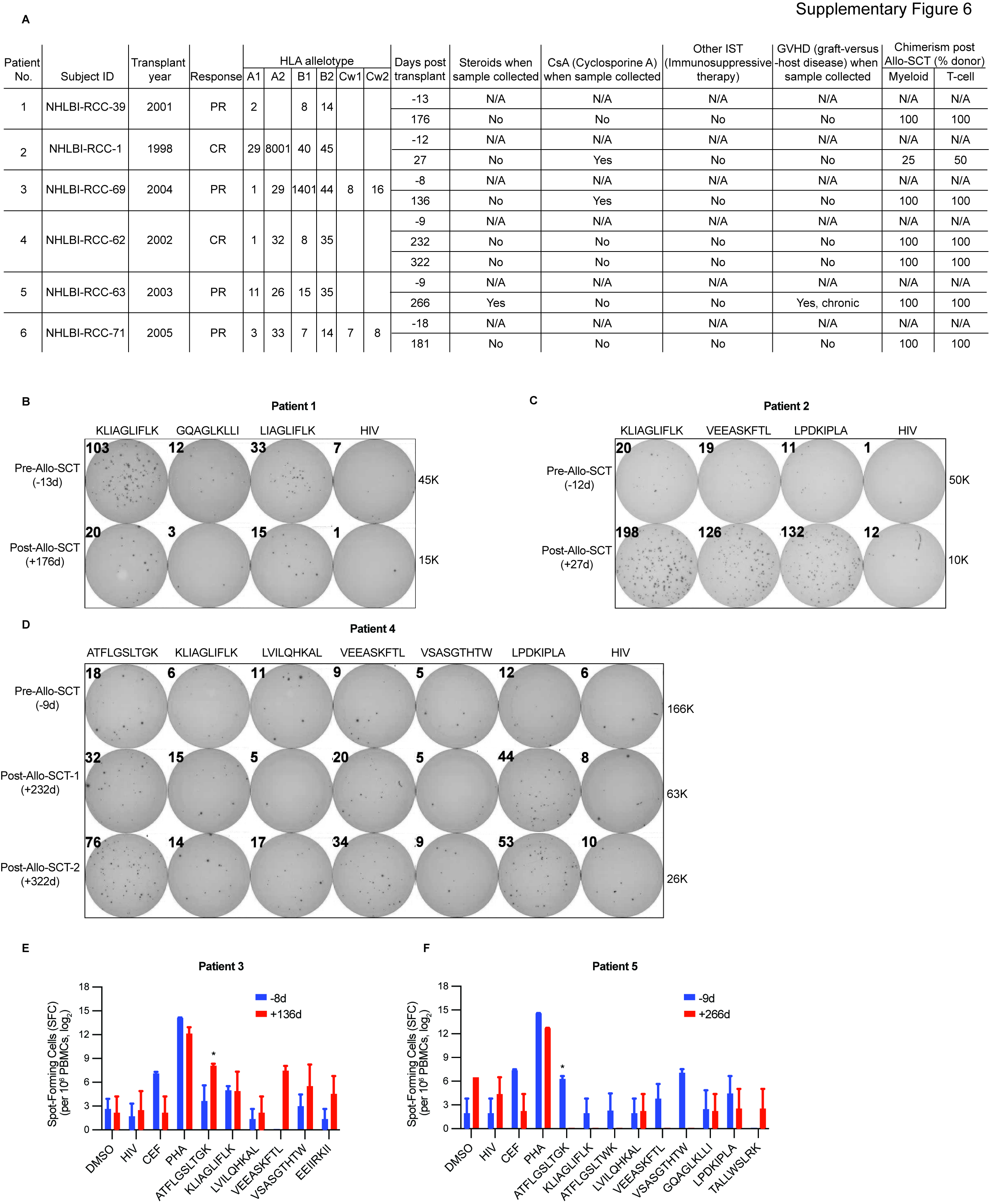

Supplement: 12 — Figure S6. ERV-derived Peptides Recognized by T cells from Human Kidney Cancer Patients, related to Figure 7 (A) Clinical information for RCC patients. N/A = not applicable. (B-F) Representative ELISpot images (B-D) and ELISpot quantification (spot count) (E-F) of IFNγ secreting PBMCs derived from allo-SCT RCC patients after stimulation with the indicated ERV-derived peptides. DMSO and an HIV peptide served as negative controls. CEF peptides and PHA served as positive controls. (B-D) Cell number plated in each well was listed on the right side of the images for patient 1, 2 and 4. Cell number varied because of differences in viable cell number after thawing. (E-F) Significance was determined by comparing the response to indicated peptides and to the HIV peptide for the corresponding patient. (N=3) [file NIHMS2060984-supplement-12.tif]
